# Iron triggers *Tv*PI4P5K proteostasis and Arf-mediated cell membrane trafficking to regulate PIP_2_ signaling crucial for multiple pathogenic activities of the parasitic protozoan *Trichomonas vaginalis*

**DOI:** 10.1128/mbio.01864-24

**Published:** 2024-12-23

**Authors:** Kuan-Yi Wu, Yen-Ju Chen, Shu-Fan Lin, Hong-Ming Hsu

**Affiliations:** 1 Department of Tropical Medicine and Parasitology, College of Medicine, National Taiwan University, Taipei, Taiwan; University of California Los Angeles, Los Angeles, California, USA

**Keywords:** iron, PIP_2_, lysosomal degradation, ADP ribosylation factor, plasma membrane trafficking, actin cytoskeleton, pathogenicity, *Trichomonas vaginalis*

## Abstract

*Trichomonas vaginalis* is the etiologic agent of trichomoniasis, one of the most common non-viral sexually transmitted infections globally. Our previous work reported the role of phosphatidylinositol 4,5-bisphosphates (PIP_2_) signaling in the actin-dependent pathogenicity of *T. vaginalis*. This study further demonstrated that iron transiently regulated *T. vaginalis* phosphatidylinositol-4-phosphate 5-kinase (*Tv*PI4P5K) proteostasis and its complex formation with an active ADP ribosylation factor *Tv*Arf220, facilitating co-trafficking to the plasma membrane, crucial for PIP_2_ production. In dominant-active HA-*Tv*Arf220 Q71L mutant, *Tv*PI4P5K plasma membrane trafficking, PIP_2_ production, and intracellular calcium levels were increased, while these processes were inhibited in dominant-negative T31N mutant or those by Brefeldin A (BFA) treatment. Additionally, PIP_2_ replenishment reversed these inhibitions in the T31N mutant, suggesting the critical role of *Tv*Arf220 activation in PIP_2_-calcium signaling. Also, T31N mutant and BFA treatment impaired actin dynamics and cytoskeleton-dependent processes in *T. vaginalis*, further linking the role of *Tv*Arf220 to PIP_2_-calcium-dependent actin dynamics. Beyond cytoadherence, during host-parasite interactions, *Tv*Arf220 influenced both contact-dependent and -independent cytotoxicity, as well as phagocytotic capacity, contributing to the cytopathogenesis of human vaginal epithelial cells. Our findings underscore the key upstream regulation mechanisms of the PIP_2_ signaling, orchestrating the interplay between *Tv*Arf220-PIP_2_-calcium signaling and downstream actin cytoskeleton-driven pathogenicity in *T. vaginalis*.

**IMPORTANCE:**

*Trichomonas vaginalis* actin cytoskeleton-centric pathogenicity is regulated by the phosphatidylinositol 4,5-bisphosphates (PIP_2_)-triggered calcium signaling cascade in response to environmental iron, though the detailed mechanism by which iron modulates PIP_2_ signaling remains unclear. Our findings reveal that iron rapidly induces *T. vaginalis* phosphatidylinositol-4-phosphate 5-kinase (*Tv*PI4P5K) translation followed by its degradation, while simultaneously activating *Tv*Arf220 binding, which facilitates *Tv*PI4P5K localization to the plasma membrane for PIP_2_ production. In contrast to the *Tv*Arf220 Q71L mutant, the reduced PIP_2_ production, intracellular calcium, actin assembly, morphogenesis, and cytoadherence in the dominant-negative T31N mutant were recovered by PIP_2_ supplementation, indicating the essential role of *Tv*Arf220 in PIP_2_-dependent calcium signaling. Additionally, the contact-dependent or -independent cytotoxicity, along with the phagocytosis, was impaired in the *Tv*PI4P5K- or *Tv*Arf220-deficient parasites, as well as in those treated with BAPTA or Latrunculin B. These findings highlight that *Tv*Arf220-mediated PIP_2_-calcium signaling cascade regulates actin cytoskeleton and cytopathogenicity of *T. vaginalis*. This study uncovers a novel pathogenic mechanism and suggests potential therapeutic targets for parasite control.

## INTRODUCTION

*Trichomonas vaginalis* is a parasitic protozoan that causes trichomoniasis, a non-viral sexually transmitted disease with approximately 150 million new cases annually (1). The parasite colonizes the host urogenital tract, causing epithelial inflammation associated with human immunodeficiency virus transmission (2) and urogenital carcinogenesis (3, 4). *T. vaginalis* adheres to host epithelia through surface adhesion molecules (5–10) and saccharide moieties (11, 12), while secreting multiple virulence factors that mediate parasitic cytoadherence, cytotoxicity, degradation of immunoglobulins, or host cell apoptosis (9, 13–15). Cysteine peptidase secretion via a lysosome-dependent pathway has been implicated in *T. vaginalis* cytopathic effects (16). By lysing host cells and phagocytosing debris, *T. vaginalis* disrupts epithelial barrier, causing permeability dysregulation (17, 18).

Iron is a critical nutrient for *T. vaginalis* growth and plays a key role in epigenetically (19) or transcriptionally (20) regulating the expression of virulence genes, involved in metabolism (19, 21), host attachment (19, 22), and proteolytic degradation (23, 24), facilitating immune evasion and cytopathic effects (23–25). Additionally, iron influences the morphological transformation of *T. vaginalis* by modulating actin dynamics, further contributing to the parasite’s pathogenicity (26). Iron also regulates *T. vaginalis* phosphatidylinositol-4-phosphate 5-kinase (*Tv*PI4P5K) expression, enhances phosphatidylinositol 4,5-bisphosphates (PIP_2_) production, and intracellular calcium levels, thereby coordinating actin cytoskeleton dynamics with various cytopathic effects (27).

In higher eukaryotes, the actin cytoskeleton diversely regulates cell morphology, secretion, protein trafficking, adhesion, locomotion, and phagocytosis (28–30). In *T. vaginalis,* flagellate-amoeboid transition and cytoadherence are mediated by actin dynamics and are inhibited by actin polymerization blocker, highlighting the role of the actin cytoskeleton in host colonization (26, 31). Depletion of PIP_2_ or intracellular calcium reduces actin dynamics, impairing amoeboid morphogenesis, cytoadherence, and contact-dependent cytotoxicity, all influenced by environmental iron (27). However, the mechanisms by which iron modulates the PIP_2_ pathway to coordinate actin dynamics and cytopathogenicity of *T. vaginalis* remain to be elucidated.

PI(4,5)P_2_ is a critical lipid that functions as a second messenger or cofactor in signaling pathways, cytoskeleton organization, membrane trafficking, cell polarity, and regulation of ion channels and transporters (32, 33). Located on the inner plasma membrane, PIP_2_ modulates the actin cytoskeleton, with intracellular PIP_2_ levels positively correlating with F-actin formation (34). It promotes nucleation of actin polymerization on the plasma membrane by dissociating the profilin-G-actin complex (35) and stabilizes actin filaments by blocking actin-severing proteins like gelsolin and cofilin (36, 37). PIP_2_ also facilitates actin assembly by removing CapZ from the filament barbed ends to allow G-actin integration (38), and recruits proteins necessary for actin branching and linking cytoskeleton to the membrane (39–41). It organizes the membrane architecture and tension pivotal to cellular adhesion (42). PIP_2_ hydrolysis by phospholipase C (PLC) produces inositol diacylglycerol (DAG) and inositol 1,4,5-trisphosphate (IP_3_), which evoke calcium-activated gelsolin and cofilin severing activity, depolymerizing actin filament (43). The crosstalk of PIP_2_ signaling and cytoskeleton remodeling varies across cell types.

In *T. vaginalis,* a *Tv*PI4P5K generates plasma membrane PIP_2_, essential for calcium signaling and pathogenicity (27). PIP_2_ hydrolysis via the PLC pathway induces extracellular calcium influx, activating actin dynamics (27). Although iron is known to regulate *Tv*PI4P5K expression and plasma membrane localization, the underlying mechanism remains unclear. In higher eukaryotes, PIP_2_/PI4P5K localization is regulated by Golgi membrane trafficking, linking PIP_2_ signaling to Rab GTPase-mediated transport (44). Arf6, in PC-12 cells, modulates PIP_2_ synthesis and PIP5K plasma membrane trafficking, essential for Ca^2+^-dependent dense-core vesicle exocytosis (45). ARF6 activation also drives actin assembly (46), membrane protrusion and invagination (47, 48), pinocytosis (49), and endocytosis via clathrin-dependent (50–52) or -independent (53–55) pathways. This study explores how iron regulates *Tv*PI4P5K expression and its plasma membrane trafficking via active Arf, which initiates upstream PIP_2_ signaling and triggers *T. vaginalis* pathogenicity.

## RESULTS

The intricate crosstalk between the cytoskeleton and plasma membrane signaling drives cell morphogenesis. Our recent findings reported that *T. vaginalis* morphogenesis and cytoadherence are driven by the actin cytoskeleton (31) and regulated through a PIP_2_-calcium signaling cascade (27), highlighting the role of iron coordinating PIP_2_ and cytoskeleton regulation to parasite virulence underlying host-parasite interactions.

### Iron transiently triggers PIP_2_ signaling in *T. vaginalis*

**Fig 1:**
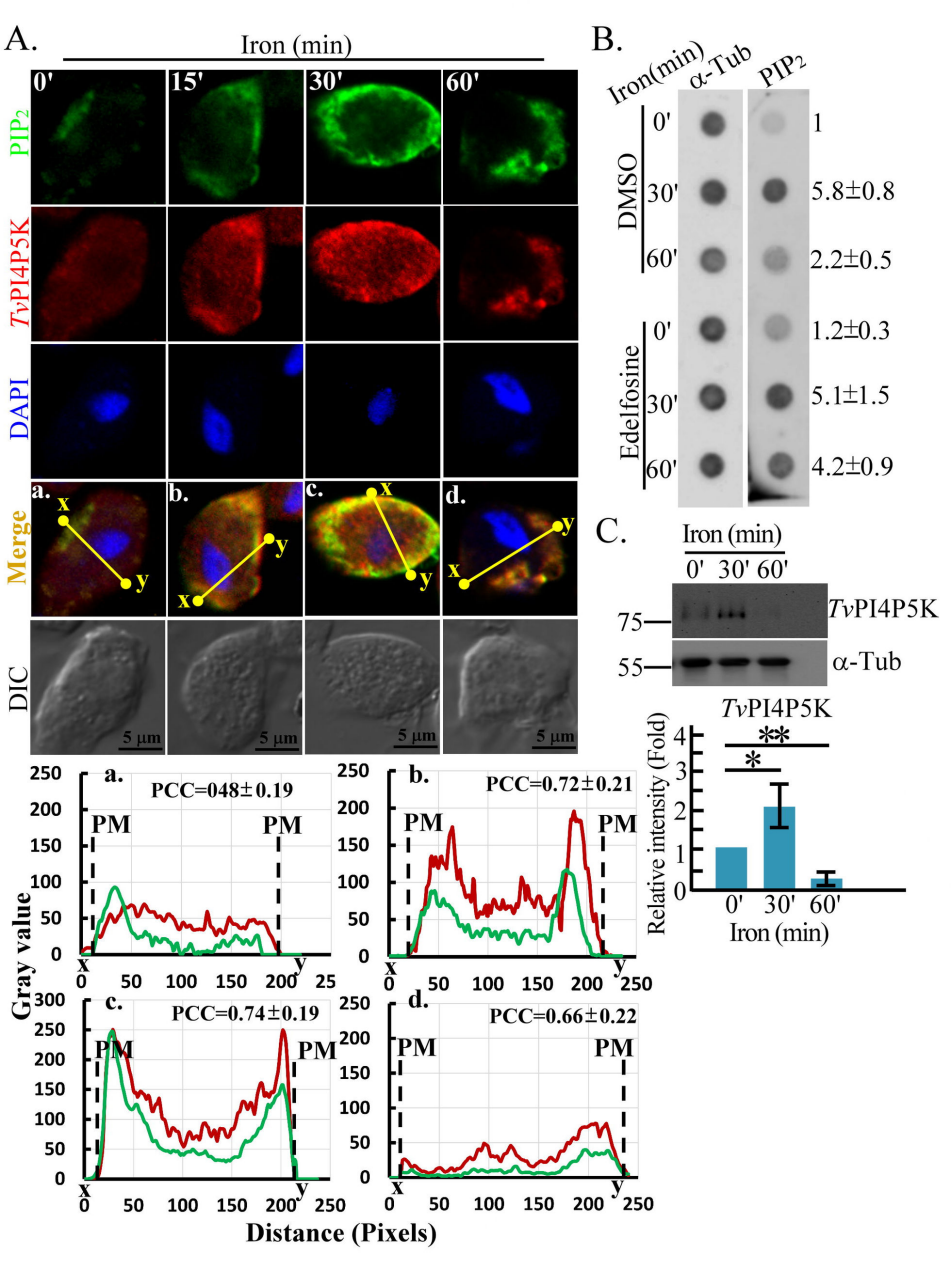
Iron transiently triggers PIP_2_ signaling in *T. vaginalis*. (**A**) TH17 iron-depleted flagellate trophozoites were replenished with iron and then fixed at indicated time points for PIP_2_ and *Tv*PI4P5K detection using IFA. Observations were conducted on over 150 trophozoites across five microscopic fields. The signal intensities between the x and y sites of the yellow lines in the representative micrographs were analyzed by ImageJ as shown in the corresponding plots (a–d). The Pearson correlation coefficient (PCC) values were measured from 30 trophozoites of three independent microscopic fields. The averaged PCC values from three biological repeats were presented in the corresponding plots (a–d) (*n* = 3, mean ± SD). PM indicates the plasma membrane boundary. (**B**) The protein lysates from iron-depleted trophozoites pretreated with DMSO and edelfosine before and after iron repletion at indicated time points were subjected to a dot blot for PIP_2_ and α-tubulin detection. The relative intensity of the PIP_2_ signal normalized to α-tubulin from three biological repeats is shown in the right of the panel (*n* = 3, mean ± SD). (**C**) The total lysates extracted from the iron-depleted trophozoites at specific times post-iron repletion were subjected to western blotting detecting *Tv*PI4P5K or α-tubulin. The relative intensity of the *Tv*PI4P5K signal normalized to α-tubulin from three biological repeats was quantified as shown in the bar graph (*n* = 3, mean ± SD) and statistically analyzed by Student’s *t*-tests with *P* < 0.05 (*) and *P* < 0.01 (**), and ns, no significance. DMSO, Dimethyl sulfoxide. IFA, Immunofluorescence assay. DAPI, 4′,6-diamidino-2-phenylindole.

*Tv*PI4P5K expression is associated with environmental iron levels (27). To further test the transient effects of iron on the PIP_2_ dynamic, we monitored PIP_2_ expression in *T. vaginalis* before and after short-term iron repletion. After iron repletion, cell membrane PIP_2_ signal increased, plateaued at 30 min with an overall intensity over fivefold higher than baseline, and returned to basal levels by 60 min (Fig. 1A; Fig. S1). Upon 30-min iron repletion, the maximal *Tv*PI4P5K signal concentrated to the plasma membrane, partially colocalizing with PIP_2_ (Fig. 1A; Fig. S1). Dot blot assay showed that iron-induced PIP_2_ fluctuations within 60-min iron repletion were inhibited by the phospholipase C inhibitor edelfosine (Fig. 1B), indicating the role of PLC in iron-regulated PIP_2_ hydrolysis. Iron transiently increased *Tv*PI4P5K expression (Fig. 1C) in parallel with PIP_2_ production (Fig. 1B), implying a correlation between iron-induced PIP_2_ levels and *Tv*PI4P5K expression.

### Iron regulates *Tv*PI4P5K translation and lysosomal degradation

**Fig 2:**
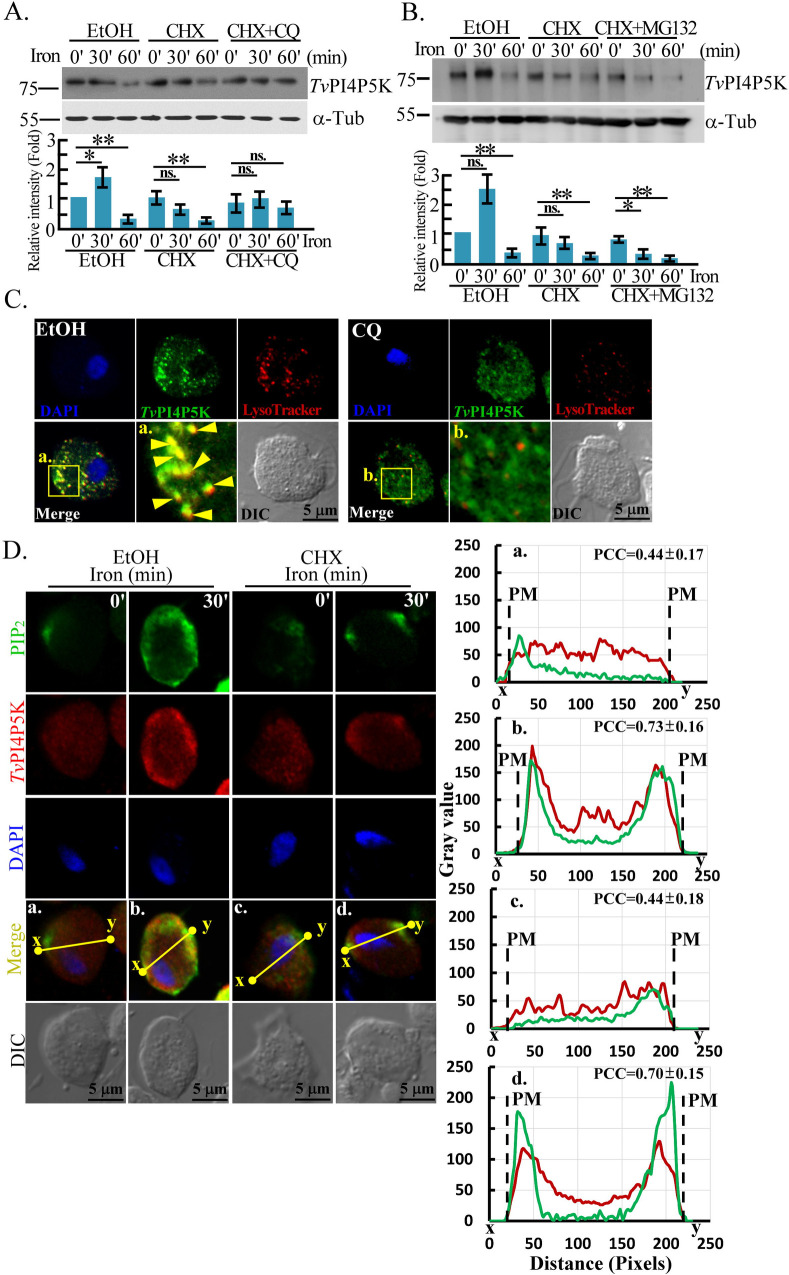
Iron regulates *Tv*PI4P5K translation and degradation. The iron-depleted trophozoites pretreated with cycloheximide (CHX) in the presence of chloroquine (CQ) (**A**) or MG-132 (**B**) were replenished with iron. The total lysates (TL) were subjected to western blotting using anti-*Tv*PI4P5K or α-tubulin antibodies. The relative signal intensities were quantified from three biological repeats as shown in the corresponding bar graphs (*n* = 3, mean ± SD) and statistically analyzed by Student’s *t*-tests, with *P* < 0.05 (*) and *P* < 0.01 (**), and ns, no significance. (**C**) The iron-depleted parasites pretreated with CQ were replenished with iron for 45 min for double-staining of LysoTracker dye and anti-*Tv*PI4P5K antibody. The boxed region in the micrograph is magnified, as shown in (**A**) and (**B**). The yellow arrowheads indicate the superimposed signal. (**D**) Iron-depleted TH17 trophozoites pretreated with CHX were challenged with iron. The trophozoites were fixed at specific times for IFA double-staining using anti-PIP_2_ and anti-*Tv*PI4P5K antibodies. The signal intensities between the x and y sites on the yellow lines of the representative micrographs measured by ImageJ were shown in the corresponding plots (a–d). The Pearson correlation coefficient (PCC) values were measured from 30 trophozoites of three independent microscopic fields. The PCC values averaged from three biological repeats were shown in the plots (a–d) (*n* = 3, mean ± SD). PM indicates the plasma membrane boundary. IFA, Immunofluorescence assay. DAPI, 4′,6-diamidino-2-phenylindole.

A previous report showed that iron does not affect *tvpi4p5k* gene transcription (27). To explore whether iron regulates *Tv*PI4P5K expression post-transcriptionally, we pretreated parasites with cycloheximide to block protein synthesis. Iron-induced *Tv*PI4P5K expression plateaued at 30 min and declined after 60 min of iron repletion (Fig. 2A), suggesting that iron modulates *Tv*PI4P5K translation and proteolytic turnover. Post 60 min of iron repletion, chloroquine (Fig. 2A), but not MG-132 (Fig. 2B), prevented the decay of *Tv*PI4P5K signal and further accumulated its iron-induced levels (Fig. S2A). Chloroquine treatment also reduced lysosome signal intensity and its colocalization with *Tv*PI4P5K, indicating lysosomal degradation of *Tv*PI4P5K (Fig. 2C). Notably, despite chloroquine repressing iron-triggered *Tv*PI4P5K expression, its plasma membrane localization remained detectable upon iron challenge (Fig. 2D; Fig. S2B), speculating that iron also regulates *Tv*PI4P5K membrane trafficking.

### Iron induces *Tv*PI4P5K membrane trafficking through *Tv*Arf220 activation

**Fig 3:**
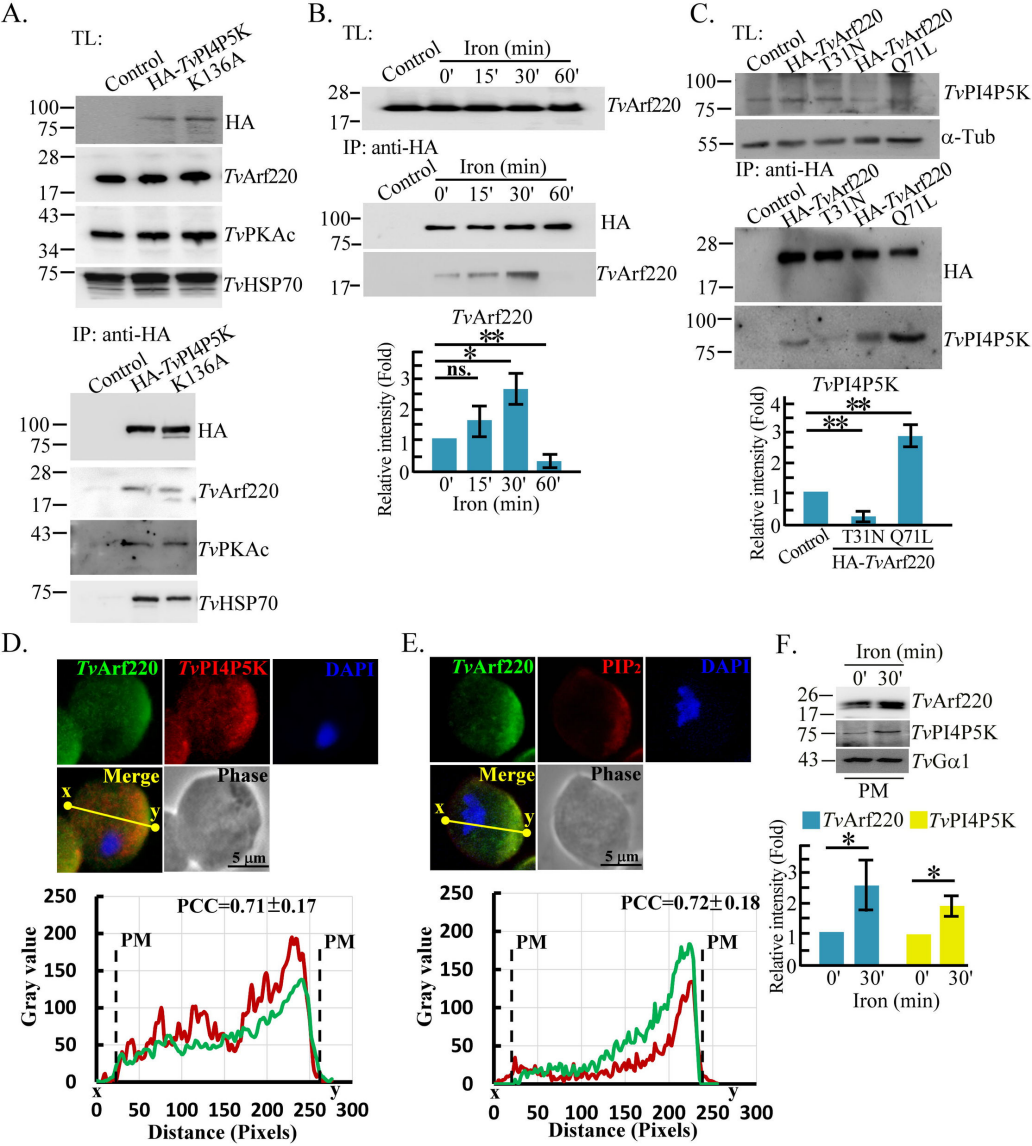
*Tv*Arf220 regulates *Tv*PI4P5K membrane trafficking. (**A**) The total protein lysates (TL) from the non-transgenic trophozoites and transfectants overexpressing HA-*Tv*PI4P5K Wt and K136A mutant were immunoprecipitated (IP) with anti-HA antibody for western blotting. (**B**) The iron-depleted non-transgenic control and transfectants overexpressing HA-*Tv*PI4P5K Wt were replenished with iron at indicated times. The TL from the distinct parasites were IP with an anti-HA antibody for western blotting. (**C**) The TL from the non-transfectant or HA-*Tv*Arf220 Wt, T31N, and Q71L transfectants were IP with an anti-HA antibody for western blotting. The parasites were fixed for IFA using anti-*Tv*Arf220 antibody double-stained with anti-*Tv*PI4P5K (**D**) or anti-PIP_2_ (**E**) antibodies. (**F**) The plasma membrane (PM, no. 1 and 2) fractions enriched by OptiPrep ultracentrifugation from the parasite before or after iron repletion were pooled for western blotting using antibodies as indicated. For (**B**), (**C**), and (**F**), the relative signal intensities of western blotting were averaged from three biological repeats as shown in the bar graphs (*n* = 3, mean ± SD) and statistically analyzed by Student’s *t*-tests, with *P* < 0.05 (*) and *P* < 0.01 (**), and ns, no significance. For (**D**) and (**E**), the signal intensities between sites x and y on the yellow lines in the representative micrographs were analyzed by ImageJ as shown in the corresponding plots. The Pearson correlation coefficient (PCC) values were measured from 30 trophozoites of three independent microscopic fields. The averaged PCC values from three biological repeats were presented in the plots (*n* = 3, mean ± SD). PM indicates the plasma membrane boundary. IFA, Immunofluorescence assay. DAPI, 4′,6-diamidino-2-phenylindole.

In the preliminary IP-proteomic analysis (Fig. S3A), *Tv*PI4P5K formed protein complexes with an ADP ribosylation factor (*Tv*Arf220, TVAGG3_0070080), a protein kinase A catalytic subunit (*Tv*PKAc, TVAGG3_1058180), and heat shock protein 70 (*Tv*HSP70, TVAGG3_0300820) in a kinase activity-independent manner (Fig. 3A). *Tv*Arf220, mouse Arf1 (*m*Arf1, P84078), and Arf6 (*m*Arf6, P62331) shared over 70% sequence identity and consensus sequences in the Switch and P-loop domains, whose conformations depend on the nature of the guanosine nucleotide (Fig. S3B) (56). Based on the dominant-negative T27A, without GTP-binding activity, and the constitutively active Q67L, without GTPase activity, of *m*Arf6, we generated the vectors expressing the wild-type (Wt), T31N, and Q71L of HA-*Tv*Arf220 for functional assays (Fig. S3C) (45). Western blotting detected a ~20 kDa endogenous *Tv*Arf220 signal in the *T. vaginalis* total lysate (Fig. S3D). Although *Tv*Arf220 expression was unchanged in total lysates, it transiently interacted with HA-*Tv*PI4P5K following 30-min iron repletion (Fig. 3B). The expression vector can overexpress HA-*Tv*Arf220 and its mutants at levels 3.5-fold higher than the endogenous ones (Fig. S3E). When HA-*Tv*Arf220 and mutants were equally expressed, *Tv*PI4P5K co-immunoprecipitated with HA-*Tv*Arf220 was enhanced in Q71L but reduced in T31A mutant (Fig. 3C). A GST-GGA3-PBD-based pull-down assay revealed that GTP-bound form of *Tv*Arf220 was transiently detectable after 30-min iron repletion (Fig. S3F), suggesting that iron may switch *Tv*Arf220 to its active GTP-bound form required for interacting *Tv*PI4P5K. *TvArf220 was mainly localized in the cytoplasm with partial enrichment around the plasma membrane*, where positively colocalizing with *Tv*PI4P5K (Fig. 3D; Fig. S4A) and PIP_2_ (Fig. 3E; Fig. S4B). In plasma membrane fractions enriched by OptiPrep ultracentrifugation (Fig. S4C), we observed increased *Tv*Arf220 and *Tv*PI4P5K signal intensities after 30 min of iron stimulation (Fig. 3F), where *Tv*Gα1 levels remained constant.

Overexpression of HA-*Tv*Arf220 Wt and the Q71L, but not T31N mutant, enhanced PIP_2_ signal around the plasma membrane, with a positive correlation of their membrane localization (Fig. S4D). This suggests that *Tv*Arf220 activation is crucial for *Tv*PI4P5K membrane trafficking and PIP_2_ production. Remarkably, the Q71L mutation elevated PIP_2_ signals not only on the plasma membrane but also in the cytoplasm, suggesting that constitutive Q71L activation perturbs *Tv*PI4P5K localization, resulting in PIP_2_ dislocation (Fig. S4D). In summary, iron evokes *Tv*Arf220 activation to bind *Tv*PI4P5K, promoting their co-trafficking to the plasma membrane.

### *Tv*Arf220 regulates *Tv*PI4P5K plasma membrane trafficking for PIP_2_ production

**Fig 4:**
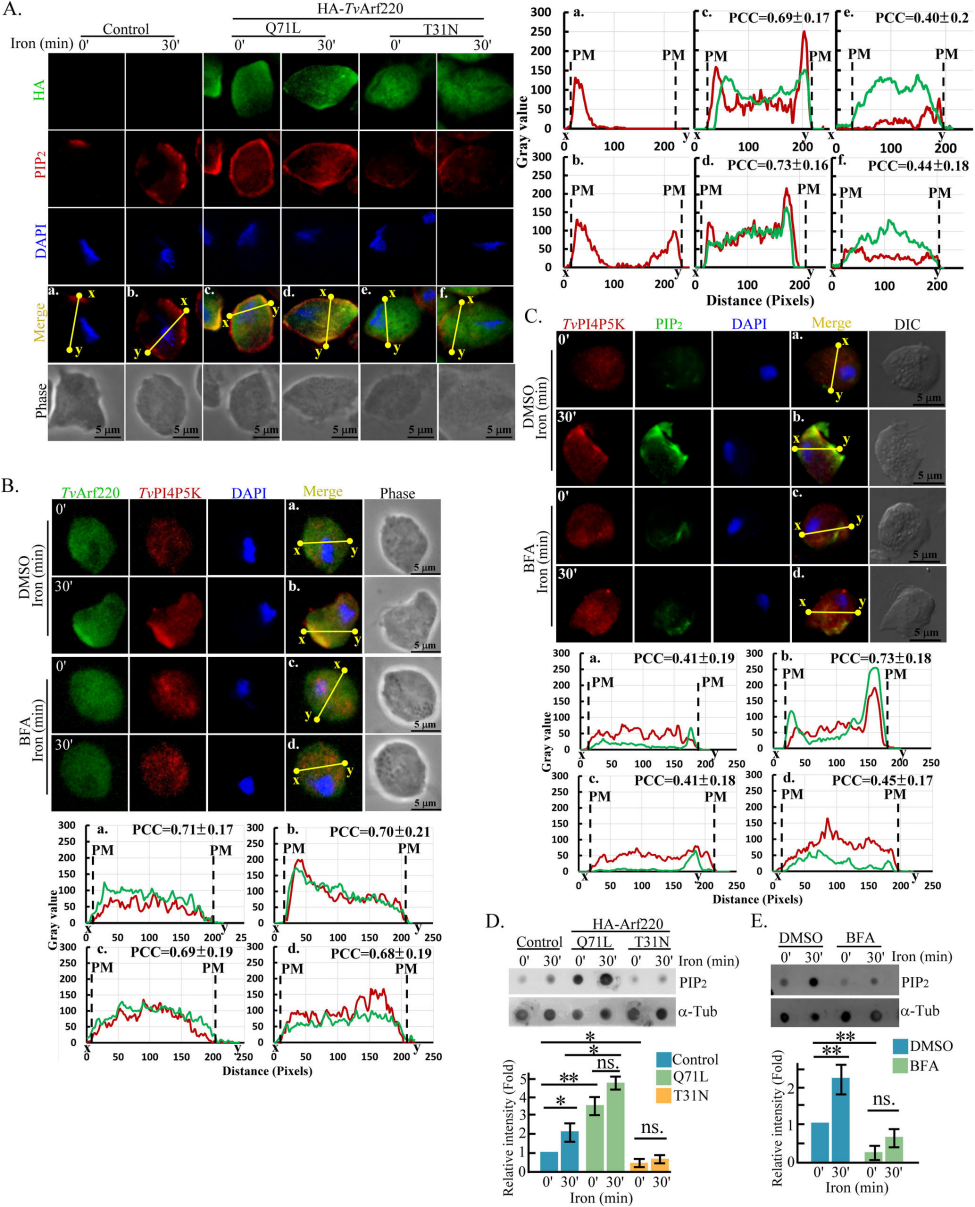
*Tv*Arf220 regulates *Tv*PI4P5K plasma membrane trafficking for PIP_2_ production. (**A**) The iron-depleted non-transfectant and HA-*Tv*Arf220 transfectants overexpressing Q71L or T31N mutants before (0′) and after 30 min (30′) of iron repletion were fixed for IFA double-staining with anti-HA and anti-PIP_2_ antibodies. (**B**) The iron-depleted parasites pretreated with DMSO or BFA were challenged with iron for 30 min (30′). The samples were fixed for IFA double-staining with anti-*Tv*PI4P5K and anti-*Tv*Arf220 (**B**) or anti-PIP_2_ (**C**) antibodies. The signal intensities on the yellow lines between the x and y sites in the representative micrographs were analyzed by ImageJ as shown in the corresponding plots. The Pearson correlation coefficient (PCC) values measured from 30 trophozoites of three independent microscopic fields. The averaged PCC values from three biological repeats were shown in the corresponding plots (*n* = 3, mean ± SD). PM indicates the plasma membrane boundary. The total lysates under conditions of (**A**) or (**B**) were subjected to a dot blot assay for PIP_2_ and α-tubulin detection as shown in (**D**) and (**E**), respectively. The relative signal intensities are averaged from three biological repeats as shown in the bar graphs (*n* = 3, mean ± SD) and statistically analyzed by Student’s *t*-tests, with *P* < 0.05 (*) and *P* < 0.01 (**), and ns, no significance. DMSO, Dimethyl sulfoxide. IFA, Immunofluorescence assay. DAPI, 4′,6-diamidino-2-phenylindole.

Dynamic monitoring of iron effects on *Tv*Arf220 regulation revealed that plasma membrane PIP_2_ in non-transgenic parasites was activated within 30 min of iron repletion. In HA-*Tv*Arf220 transfectants, the Q71L mutant elevated basal PIP_2_ levels in iron-depleted trophozoites, while the T31N mutant abolished the iron-induced PIP_2_ production following iron exposure (Fig. 4A; Fig. S5A). Regardless of iron presence, the Q71L signal was localized mainly in the cytoplasm but partial colocalizing with PIP_2_ around the plasma membrane, while the T31N signal only distributed in the cytoplasm (Fig. 4A). Arf inhibitor Brefeldin A (BFA) did not significantly affect cytoplasmic distribution of *Tv*Arf220 and *Tv*PI4P5K but inhibited their iron-induced co-trafficking to the plasma membrane (Fig. 4B; Fig. S5B) and simultaneously reduced the plasma membrane PIP_2_ levels (Fig. 4C; Fig. S5C). Also, the basal and iron-regulated total PIP_2_ levels were increased in the Q71L mutant but decreased in the T31N mutant (Fig. 4D) or BFA-treated parasites (Fig. 4E). BFA exposure did not affect endogenous *Tv*Arf220 and *Tv*PI4P5K levels (Fig. S5D) or parasite viability (Fig. S5E). In summary, iron likely activates *Tv*Arf220 to direct *Tv*PI4P5K transport to the cell membrane for PIP_2_ production.

### *Tv*Arf220 regulates *T. vaginalis* intracellular calcium via the PIP_2_ pathway

**Fig 5:**
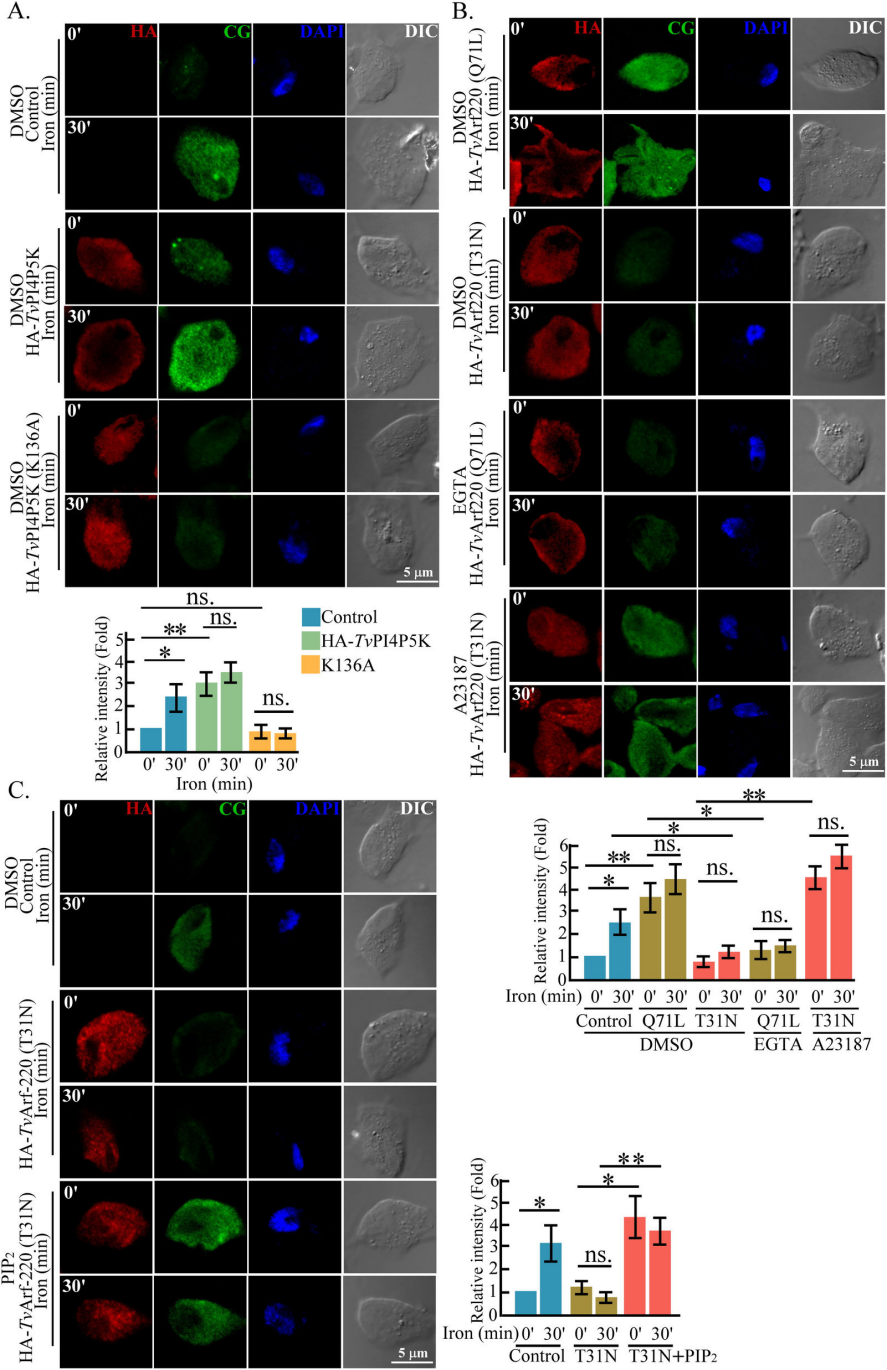
PIP_2_ signaling regulates *T. vaginalis* intracellular calcium. (**A**) The non-transgenic control and HA-*Tv*PI4P5K Wt or K136A transfectants preloaded with CG fluorescence dye were fixed at specific times post-iron repletion for IFA. (**B**) The CG-preloaded non-transgenic trophozoites and HA-*Tv*Arf220 Q71L or T31N transfectants with EGTA or A23187 treatment were replete of iron for IFA. (**C**) The CG-prelabeled non-transfectant and HA-*Tv*Arf220 T31N transfectant without or with PIP_2_ supplementation were replete with iron and fixed for IFA. The relative intensity of the CG signal was quantified from 150 trophozoites in five microscopic fields. The averages from three biological replicates were shown in the bar graph (*n* = 3, mean ± SD) and statistically analyzed by Student’s *t*-tests, with *P* < 0.05 (*), *P* < 0.01 (**), and ns, no significance. DMSO, Dimethyl sulfoxide. IFA, Immunofluorescence assay. DAPI, 4′,6-diamidino-2-phenylindole.

*Tv*PI4P5K has been shown to regulate calcium levels in *T. vaginalis* (27). To validate whether *Tv*Arf220 mediates PIP_2_ production and affects intracellular calcium levels, we utilized the calcium indicator Calcium Green (CG) for fluorescence detection. In HA-*Tv*PI4P5K Wt transfectant, basal CG signal was elevated to levels comparable to those induced by iron, whereas the elevation was restrained in the K136A mutant, indicating a role of PIP_2_ in calcium regulation (Fig. 5A). Compared to the non-transgenic control (Fig. 5A), basal CG intensity was enhanced in HA-*Tv*Arf220 Q71L mutant, when iron-activated CG signals were diminished in T31N mutant. Also, the Q71L-enhanced CG elevation was attenuated by the extracellular chelator EGTA, while the T31N-diminished CG signals were reversed by calcium ionophore A23187 (Fig. 5B). While EGTA or A23187 treatment had no impact on parasite vitality (27), these results demonstrated that *Tv*Arf220 activation controls extracellular calcium influx. Although iron-induced CG signals were disrupted in the T31N mutant, they were restored upon PIP_2_ recovery in the parasites (Fig. 5C; Fig. S6). These findings validate that iron regulates PIP_2_-calcium signaling in *T. vaginalis* through *Tv*Arf220 activation.

### PIP_2_ signaling modulates actin organization in *T. vaginalis*

**Fig 6:**
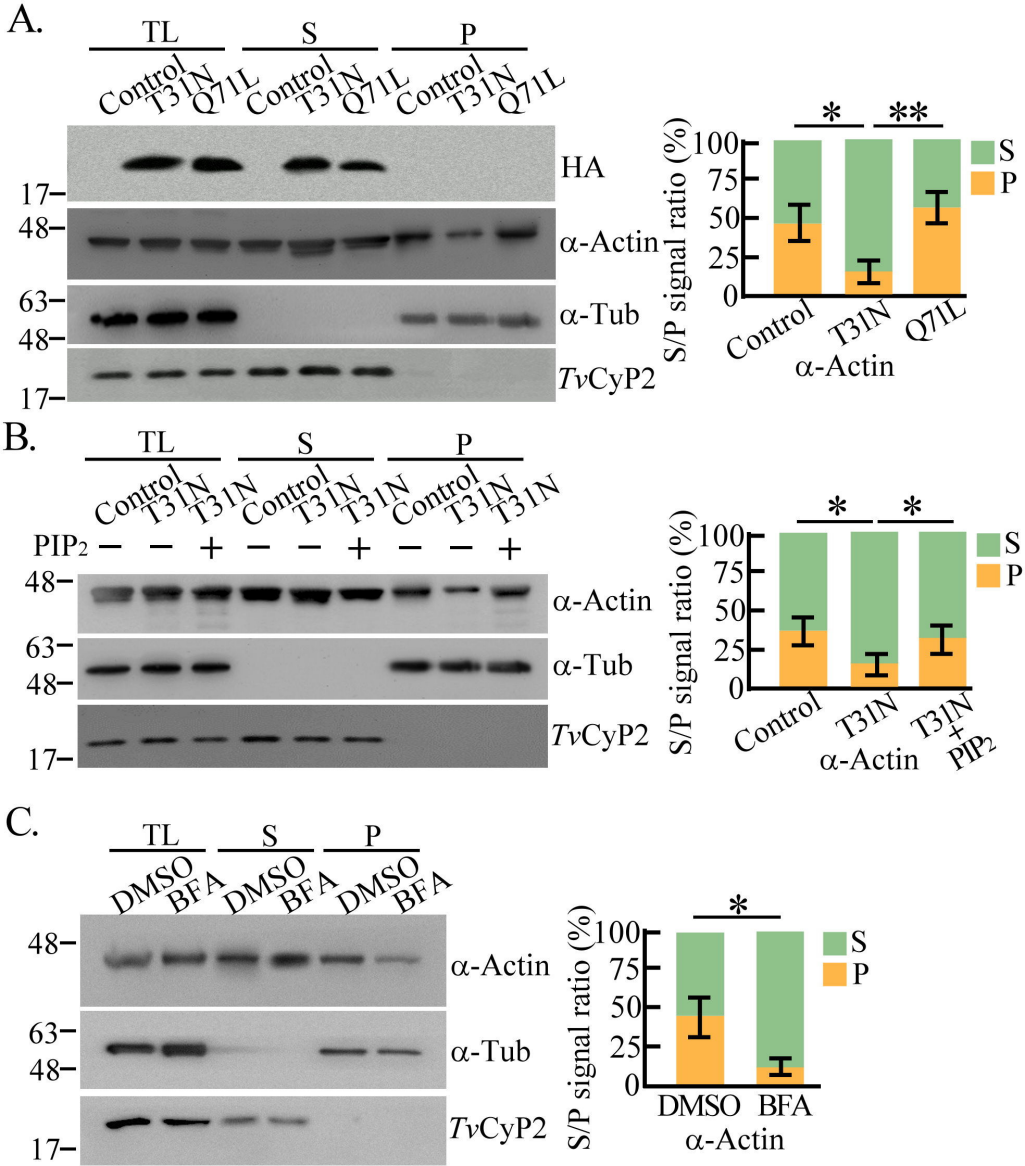
*Tv*Arf220-mediated PIP_2_ signaling modulates actin organization in *T. vaginalis*. The total lysates from the non-transgenic TH17 trophozoites and those with HA-*Tv*Arf220 T31N or Q71L overexpression (**A**), PIP_2_ supplement (**B**), or BFA treatment (**C**) were fractionated into G-actin containing supernatant (S) and F-actin containing pellet (P) fractions for western blotting. The assays were performed in triplicate. The ratio of the α-actin signal in the supernatant versus pellet fraction was quantified, as shown in the bar graphs. (*n* = 3, mean ± SD) and statistically analyzed by Student’s *t*-tests with *P* < 0.05 (*), *P* < 0.01 (**), and ns, no significant difference. DMSO, Dimethyl sulfoxide.

We investigated whether *Tv*Arf220 regulates PIP_2_ signaling to modulate the actin cytoskeleton dynamics. The transfectants overexpressing HA-*Tv*Arf220 T31N and Q71L were fractionated into the supernatant (G-actin) and pellet (F-actin) fractions to assess actin polymerization. Compared to the non-transgenic controls, the F-actin levels were unchanged in HA-*Tv*Arf220 Q71L but decreased in the T31N mutant (Fig. 6A), highlighting *Tv*Arf220 activation in actin assembly. However, T31N-reduced F-actin polymerization was recovered upon PIP_2_ recovery (Fig. 6B). Furthermore, actin assembly was also inhibited by BFA treatment (Fig. 6C), underscoring the critical role of *Tv*Arf220-mediated PIP_2_-calcium signaling in actin dynamics.

### *Tv*Arf220 involves actin-based cytoskeleton activities

**Fig 7:**
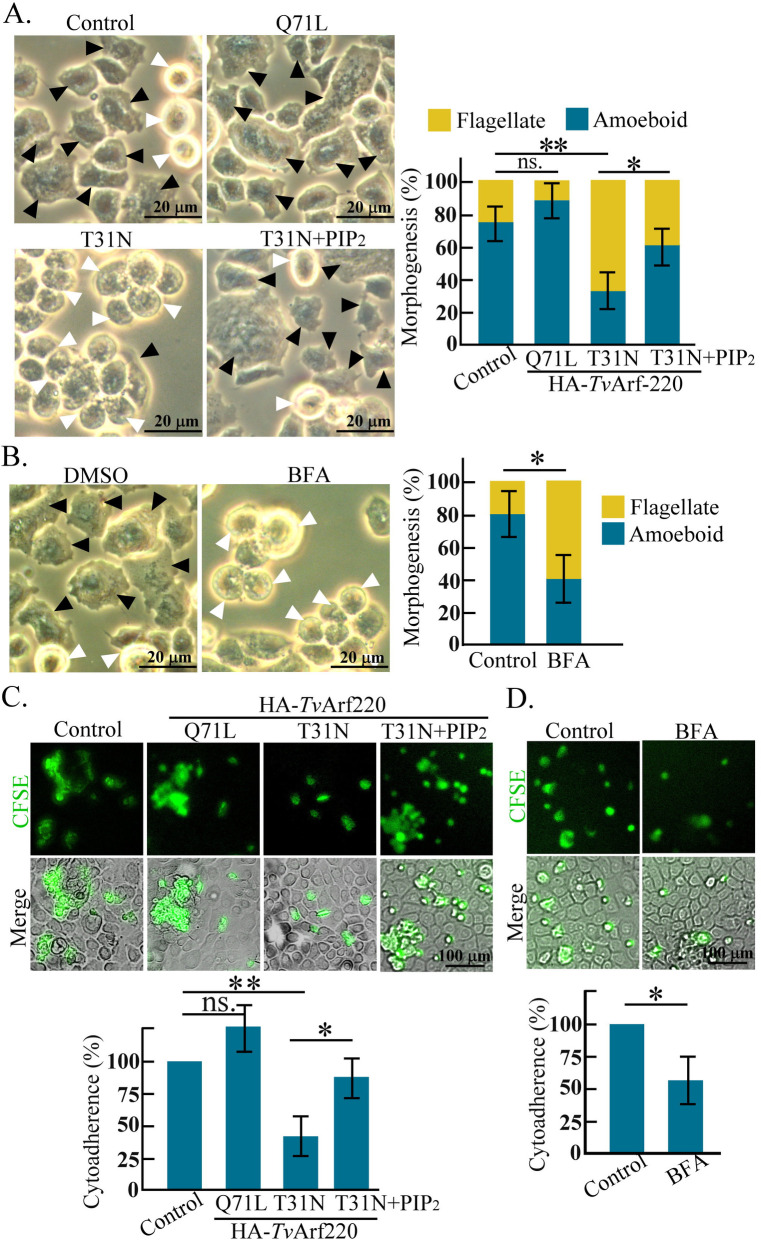
Involvement of TvArf220 in actin-based morphogenesis and cytoadherence. TH17 non-transfectant or transfectants overexpressing HA-*Tv*Arf220 Q71L and T31N or supplemented with PIP_2_ (**A**), and those treated with DMSO and BFA (**B**) were subjected to morphogenesis (A and B) or cytoadherence (C and D) assays. In the morphogenesis assay, parasite morphology was observed by phase contrast microscopy post-cultivating flagellate trophozoites in a T25 flask for 1 h. The proportion of amoeboid (black arrowheads) versus flagellate (white arrowheads) trophozoites was measured from 300 trophozoites of five independent microscopic fields, as shown in the bar graph. For the cytoadherence assay, the CFSE-labeled parasites were co-cultured with *h*VECs monolayer for 1 h. After washing the unbound trophozoites, the sample was fixed and observed by confocal microscopy. The relative CFSE intensity versus the negative control (100%) was measured to evaluate cytoadherence activity, as shown in the bar graph. The assays were performed in triplicate (*n* = 3, mean ± SD) and statistically analyzed by Student’s *t*-tests with *P* < 0.05 (*), *P* < 0.01 (**), and ns, no significant difference. DMSO, Dimethyl sulfoxide.

Amoeboid morphogenesis, essential for *T. vaginalis* cytoadherence, is regulated by actin cytoskeleton (26, 31). In HA-*Tv*Arf220 transfectants, the amoeboid transition was unchanged in the Q71L mutant but reduced in the T31N mutant (Fig. 7A) and the BFA-treated parasites (Fig. 7B). Notably, PIP_2_ supplementation restored T31N-impaired morphogenesis (Fig. 7A) and cytoadherence (Fig. 7C), while BFA treatment significantly weakened cytoadherence (Fig. 7D). These findings show that *Tv*Arf220 activation modulates actin-centric morphogenesis and cytoadherence through the PIP_2_ signal.

### *Tv*Arf220-mediated PIP_2_ signaling regulates *T. vaginalis* cytotoxic activity

**Fig 8:**
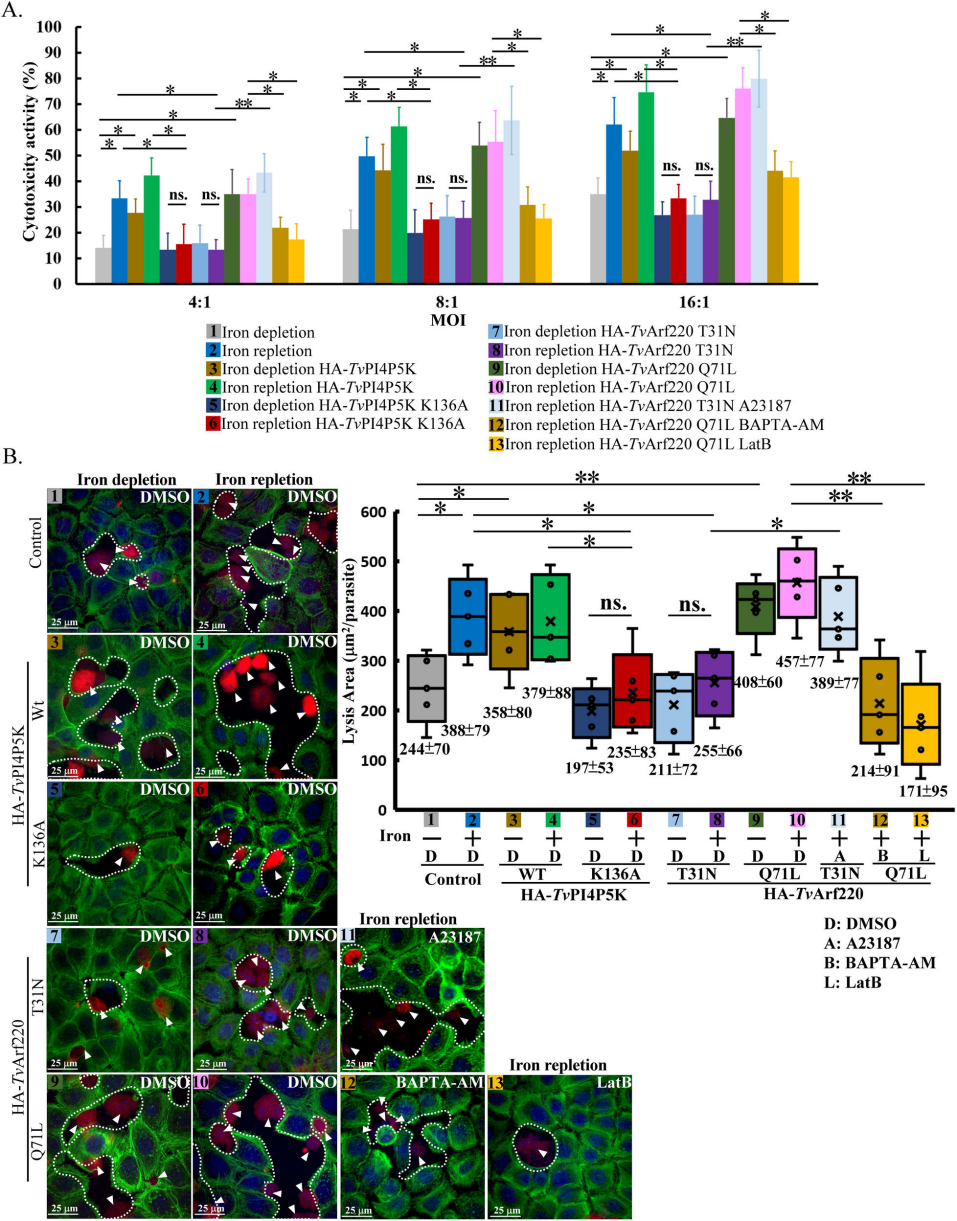
*Tv*Arf220-associated *T. vaginalis* cytopathogenic function. The TH17 non-transgenic trophozoites with or without iron depletion and the transfectants overexpressing HA-*Tv*PI4P5K Wt and K136A or HA-*Tv*Arf220 T31N and Q71L were either pretreated with A23187, BAPTA-AM, or LatB. (**A**) The parasites were co-cultured with *h*VECs at a different MOI for 4 h in the LDH cytotoxicity assay. Relative cytotoxicity (%) was measured in three biological repeats, as shown in the bar graph (*n* = 3, mean ± SD). (**B**) The CMRA-labeled parasites were co-cultured with *h*VECs (MOI = 4) for 1 h and then double-stained with FITC-phalloidin, and the nuclei were stained with DAPI. The white dashed line marks the area lysed by the parasite on the *h*VECs monolayer. The average lysis area per trophozoite was measured in three biological repeats, as shown in the Box-whisker plot (*n* = 3, mean ± SD). The grouped data were statistically analyzed by Student’s *t*-tests with the *P* < 0.05 (*), *P* < 0.01 (**), and ns, no significant difference.FITC, Fluorescein isothiocyanate. DMSO, Dimethyl sulfoxide. DAPI, 4′,6-diamidino-2-phenylindole.

*T. vaginalis* lyses human cells through contact-dependent disruption or soluble factors (9, 17, 18). To clarify the role of *Tv*Arf220 in PIP_2_-dependent cytotoxicity, *T. vaginalis* with or without iron, was inoculated onto human vaginal epithelial cells (*h*VECs) at a different multiplicity of infection (MOI) for lactate dehydrogenase (LDH) cytotoxicity assay (Fig. 8A). Cytotoxicity increased with MOI but was lower in the iron-depleted parasites, supporting iron’s contribution to cytotoxicity (Fig. 8A). In iron-replete parasites, cytotoxicity was modestly elevated in HA-*Tv*PI4P5K Wt or HA-*Tv*Arf220 Q71L, but significantly reduced in K136A or T31N mutants, resembling iron-depleted non-transgenic controls. Conversely, HA-*Tv*PI4P5K Wt and HA-*Tv*Arf220 Q71L increased cytotoxicity in iron-depleted parasites, implying *Tv*Arf220’s involvement in PIP_2_-modulated cytotoxic activity. Moreover, *Tv*Arf220 Q71L-enhanced cytotoxicity was inhibited by the intracellular calcium chelator BAPTA-AM or actin assembly inhibitor Latrunculin B (LatB), while the reduced cytotoxicity in *Tv*Arf220 T31N mutant was reactivated by the calcium ionophore A23187, indicating that *Tv*Arf220-PIP_2_-calcium signaling regulates actin cytoskeleton-dependent cytotoxicity in *T. vaginalis*. Notably, following a 4-h incubation of parasites in 5% CO_2_, over 85% viability was maintained (Fig. S7A), with low LDH activity detected in the medium supernatant (Fig. S7B), excluding LDH leakage from parasites during cytotoxicity assay.

Contact-dependent cytolysis was further assessed by measuring lysis area in *h*VECs (Fig. 8B). Iron-replete parasites showed a larger lysis area than iron-depleted ones, reaffirming iron’s pivotal role in cytolysis. HA-*Tv*PI4P5K Wt and HA-*Tv*Arf220 Q71L enhanced cytolysis in iron-depleted parasites, while K136A and T31N mutants reduced cytolysis in the iron-replete parasites. BAPTA-AM and LatB reduced cytolysis driven by HA-*Tv*Arf220 Q71L, and A23187 rescued cytolysis inhibited in the T31N mutant. It suggests the crucial role of the *Tv*Arf220-mediated PIP_2_-calcium cascade in actin dynamics and cytolysis, contributing to cytotoxicity.

### *Tv*Arf220-mediated PIP_2_ signaling modulates *T. vaginalis* contact-independent cytotoxicity

**Fig 9:**
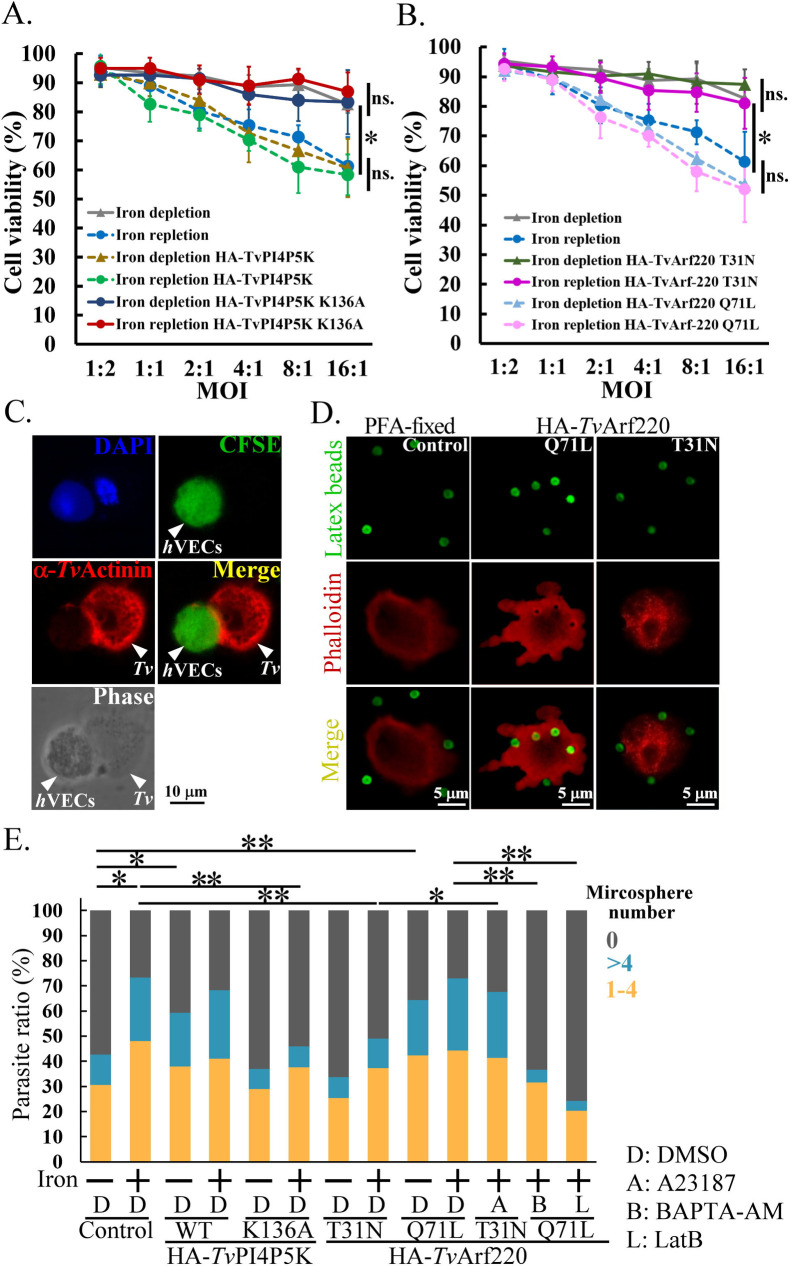
The role of *Tv*Arf220 in phagocytosis and factor release. The viabilities of *h*VECs co-cultured with the iron-depleted or iron-replete HA-*Tv*PI4P5K (**A**) or HA-*Tv*Arf220 (**B**) transfectants in transwell microplate at a different MOI for 4 h were monitored by the 3-(4,5-dimethylthiazol-2-yl)-2,5-diphenyltetrazolium bromide assay. The data were collected from three biological repeats. The relative host cell viability (%) was shown in the graphs (*n* = 3, mean ± SD) and statistically analyzed by Student’s *t*-tests, with *P* < 0.05 (*) and *P* < 0.01 (**), and ns, no significance. (**C**) The TH17 trophozoites were co-cultured with CFSE-prelabeled *h*VECs on a coverslip for 2 h and fixed for IFA staining with anti-*Tv*Actinin antibody. (**D**) Human IgG opsonized microspheres were inoculated into TH17 cultures pre-fixed with formaldehyde (PFA) or those overexpressing HA-*Tv*Arf220 Q71L and T31N mutants for 2 h and fixed for double-staining with TRITC-phalloidin and FITC-conjugated goat anti-human IgG antibody. The assays were performed in triplicate to measure the ratios of parasites containing internalized microspheres (*n* = 3, mean ± SD), as shown in (**E**).FITC, Fluorescein isothiocyanate. DMSO, Dimethyl sulfoxide. IFA, Immunofluorescence assay. DAPI, 4′,6-diamidino-2-phenylindole.

When monitoring contact-independent cytotoxicity in a transwell co-culture system, iron-replete parasites significantly caused more *h*VEC damage than iron-depleted ones. In contrast to iron-replete transfectants of HA-*Tv*PI4P5K Wt and HA-*Tv*Arf220 Q71L, *h*VECs exposed to HA-*Tv*PI4P5K K136A, HA-*Tv*Arf220 and T31N (Fig. 9A and B), or pretreated with edelfosine, BAPTA-AM, and LatB exhibited higher viability, similar to those exposed to iron-depleted parasites (Fig. S8A). Notably, reduced cytotoxicity in the T31N mutant was rescued by PIP_2_ replenishment (Fig. S8B). It suggests that PIP_2_ signaling modulates the contact-independent and -dependent cytotoxicity, although the role of secreted virulence factors remains to be explored. Intriguingly, scanning electron microscopy revealed less presence of extracellular vesicles (0.2 μm–1 μm) in the T31N mutant, and they reappeared after PIP_2_ replenishment, resembling levels in the non-transgenic control (Fig. S8C). These findings suggest that *Tv*Arf220 mediates vesicle secretion via PIP_2_ pathway. Thus, iron may provoke extracellular vesicle secretion in *h*VECs contact-independent cytotoxicity through *Tv*Arf220-mediated PIP_2_ signaling.

### *Tv*Arf220-mediated PIP_2_ signaling regulates *T. vaginalis* phagocytosis

*T. vaginalis* phagocytoses bacteria, yeast, and multiple human cells (17, 57, 58), such as *h*VECs, causing cell death (Fig. 9C). To quantify its phagocytic activity, fluorescein-labeled microspheres internalization was assessed. In contrast to the formaldehyde-fixed parasites without microsphere uptake, live parasites showed variable phagocytosis. In contrast to the HA-*Tv*Arf220 T31N mutant, the Q71L mutant showed more significant microsphere internalization, with a stronger phalloidin signal at the plasma membrane and microsphere periphery (Fig. 9D). Meanwhile, optimal phagocytosis of *T. vaginalis* required iron (Fig. 9E). Notably, HA-*Tv*PI4P5K Wt and HA-*Tv*Arf220 Q71L overexpression enhanced phagocytosis of iron-depleted parasites, whereas HA-*Tv*PI4P5K K136A and HA-*Tv*Arf220 T31N reduced that in iron-replete parasites. The data show that *Tv*Arf220-mediated PIP_2_ signaling correlates with *T. vaginalis* phagocytosis in an iron-dependent manner (Fig. 9E). Additionally, A23187 restored phagocytosis in T31N mutant under iron-replete conditions, while BAPTA-AM suppressed Q71L-mediated phagocytosis, underscoring the significance of intracellular calcium in *Tv*Arf220-mediated phagocytosis. LatB suppressed Q71L-driven phagocytosis (Fig. 9E) without affecting parasite viability (26), indicating the role of actin dynamics in *Tv*Arf220-dependent phagocytosis. In this parasite, iron likely triggers PIP_2_-dependent calcium signal via *Tv*Arf220 to coordinate actin dynamics and cytopathic effects to kill host cells.

### Conclusion

**Fig 10:**
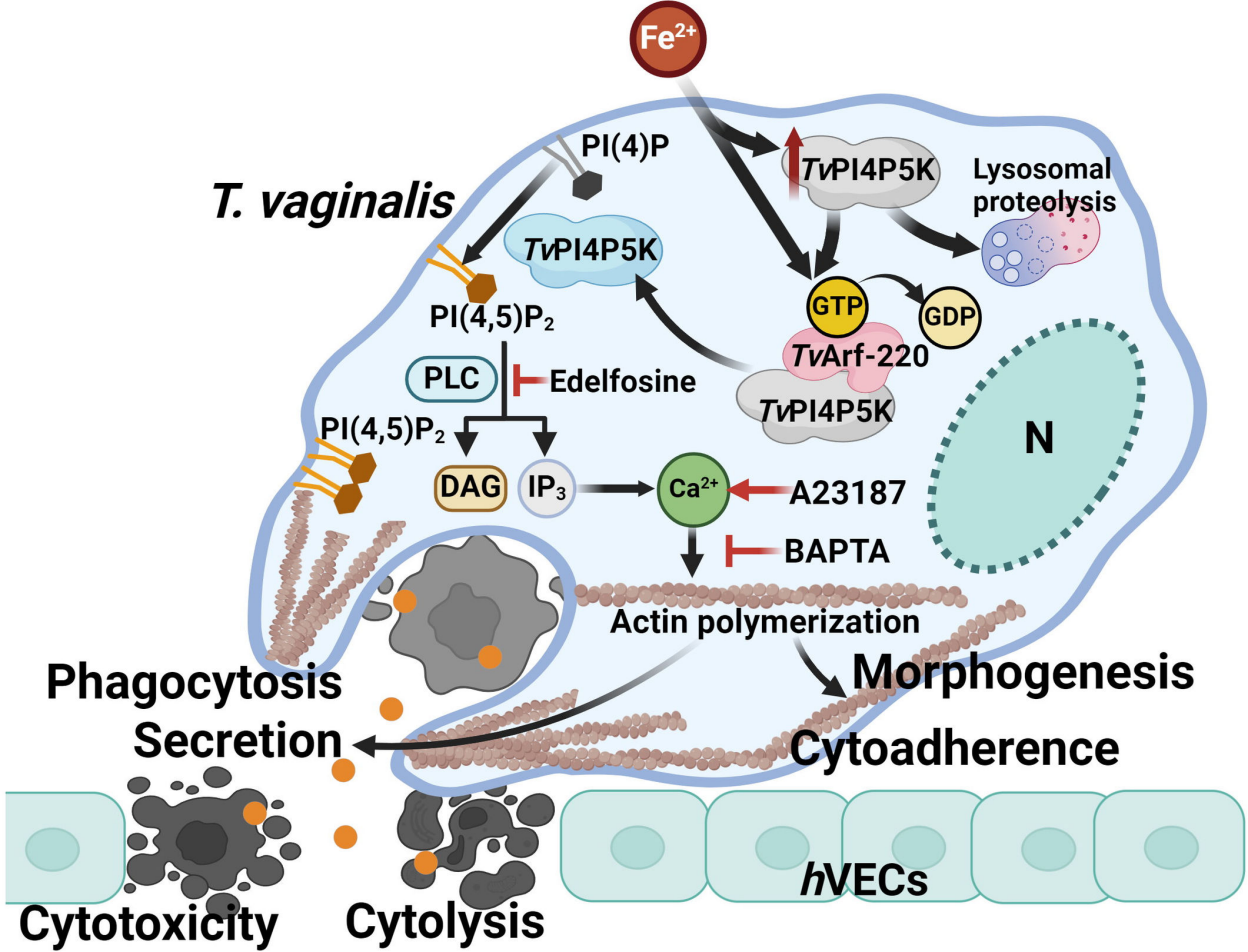
*Tv*Arf220-mediated PIP_2_ signaling in *T. vaginalis* pathogenesis. Environmental iron transiently induces *Tv*PI4P5K overexpression followed by lysosomal proteolysis and triggers the complex formation with activated *Tv*Arf220. This complex co-trafficks to the plasma membrane, where *Tv*PI4P5K phosphorylates PI(4)P into PI(4,5)P_2_, driving PLC-dependent hydrolysis, a key process required for intracellular calcium increase and subsequent actin remodeling. Actin dynamics, pivotal to parasite morphogenesis and cytoadherence, are further regulated by iron-induced PIP_2_ signaling and calcium cascades, mediated through *Tv*Arf220-directed *Tv*PI4P5K membrane transportation. These processes contribute to host cell contact-dependent cytolysis and phagocytosis or the release of cytotoxic factors against *h*VECs. The precise roles of plasma membrane PIP_2_ or intracellular calcium in parasite cytopathogenicity remain to be elucidated. In conclusion, the intricate crosstalk between *Tv*Arf220-PIP_2_-calcium signaling and cytoskeleton activities orchestrates the fully functional cytopathic effects of *T. vaginalis*. (The illustration was created with BioRender.com)

Iron modulates *T. vaginalis Tv*PI4P5K proteostasis via balancing its translation and lysosomal degradation, while also activating *Tv*Arf220 to form a complex with *Tv*PI4P5K, facilitating their co-trafficking to the plasma membrane. These processes jointly orchestrate PIP_2_ levels in this parasite. Hydrolysis of PIP_2_ via PLC induces extracellular calcium influx to activate cytoskeleton remodeling, driving amoeboid morphogenesis, cytoadherence, and cytotoxicity through contact-dependent cytolysis, phagocytosis, or secretion of virulence factors, to damage host cells. Intriguingly, PIP_2_-calcium signaling and driven cytoskeleton activities inhibited in *Tv*Arf220-deficient mutant can be rescued upon PIP_2_ recovery or calcium ionophore activation, reaffirming the critical upstream role of *Tv*Arf220 in coordinating PIP_2_-calcium signaling and actin-mediated cytopathic effects. These findings unravel new regulatory mechanisms in signal transduction of *T. vaginalis* and offer potential therapeutic strategies for parasite control (Fig. 10).

## DISCUSSION

### Iron regulation and signal transduction

Iron is an essential factor for organisms but can be toxic in excess. However, the tolerance of *T. vaginalis* exhibits a notably high tolerance to iron, suggesting the evolution of a distinct pathway enabling adaption to elevated iron levels. Cellular mechanisms that regulate iron homeostasis from prokaryotes to eukaryotes primarily focus on transcriptional and translational regulation (59, 60). In higher eukaryotes, iron homeostasis is modulated by the interaction between iron-responsive elements (IRE) within mRNA and iron-regulatory proteins (IRPs), which regulate translation efficiency in response to iron levels via post-translational mechanisms (59).

In *T. vaginalis,* iron triggers Cyclic adenosine monophosphate (cAMP)-dependent *Tv*PKAc activation, leading to the phosphorylation and nuclear impart of transcription factor (20). Intriguingly, *Tv*PI4P5K also formed a complex with *Tv*PKAc, but their function remains unknown. In yeast, iron-responsive transcriptional control is coupled with PKA and Mitogen-activated protein kinase (MAPK) signaling pathways, influencing cell growth and division (61). In bovine sperm, PKA activation elicits PIP_2_ production via Phosphoinositide 3-kinase (PI3K)-associated PI4P5K activation, promoting phospholipase D activity and actin assembly (62). Notably, edelfosine treatment consistently sustained iron-induced PIP_2_ levels at the plasma membrane (Fig. 1B), implicating PLC-dependent pathways in PIP_2_ regulation (27). Further investigation is required to clarify the interplay between these pathways.

Despite the overexpression of HA-*Tv*PI4P5K enhancing intracellular PIP_2_ levels, it failed to activate actin assembly, morphogenesis, or cytoadherence beyond basal levels in control parasites, implying that the limited PIP_2_ pool at the plasma membrane was adequate for downstream regulation in *T. vaginalis* (27). Further PIP_2_ replenishment restored cytoskeleton activities only to basal levels, indicating that the parasite’s PIP_2_ levels are tightly regulated to ensure optimal downstream reactions.

### IRE/IRP system in *T. vaginalis*

IREs are mRNA stem and loop structures that mediate post-translational control via IRPs. Classical IREs, with specific structures that bind IRPs, either block translation or stabilize associated mRNAs (63). In *T. vaginalis*, atypical IRE-like sequences with activities binding human IRPs and IRP-like proteins have been identified in the 5′ Untranslated region (UTR) of *tvcp4* (64) and 3′ UTR of *tvcp12* (65)*,* suggesting the presence of an IRE/IRP regulatory system in this primitive protozoan. The *tvpi4p5k* mRNA level remains constant in the parasites regardless of the presence of iron, and an IRE-like sequence with a UUG motif is predicted in the 5′UTR of *tvpi4p5k* mRNA (Fig. S9) (66, 67). Although cycloheximide blocks iron-induced *Tv*PI4P5K expression, whether iron regulates *Tv*PI4P5K translation via an IRE-like mechanism remains unclear.

### Plasma membrane PIP_2_

In mammalian cells, PIP_2_ localized within lipid rafts modulates distinct cellular responses (68). In *Entamoeba histolytica*, cholesterol evokes PIP_2_ accumulation in uroid lipid rafts at the trailing edge of polarized trophozoites, increasing intracellular calcium and motility (69). In T cells, plasma membrane PIP_2_ recruits activated ezrin-radixin-moesin (ERM) binding, spatially regulating actin polymerization. While deactivated ERM dissociated from the plasma membrane, its fluidity is altered to reshape the membrane structure necessary for adhesion (70).

In higher eukaryotes, PIP_2_ is cleaved by PLC into IP_3_ and DAG (71), where IP_3_ mediates intracellular calcium release (72), and DAG activates PKC (73), which in turn governs F-actin formation (74) while IP_3_ controls intracellular calcium dynamics (75). During *T. vaginalis* morphogenesis*,* PLC-dependent PIP_2_ hydrolysis mediates calcium-dependent actin dynamics (27) and membrane reorganization. Unlike calcium-dependent actin serving in higher eukaryotes (43), elevated intracellular calcium in amoeboid *T. vaginalis* trophozoites promotes F-actin assembly, driving cytoskeletal changes. This suggests divergent calcium regulation in actin dynamics of *T. vaginalis*. Calcium signaling is also essential for cell motility, invasion, and egress in apicomplexan parasites (76, 77); it underscores its vital role in *T. vaginalis* virulence.

### *Tv*Arf220 regulates *Tv*PI4P5K cell membrane trafficking

Previous studies have reported that *m*Arf6 localizes to endosomes and the plasma membrane, facilitating protein trafficking between these compartments (47, 48). *Tv*Arf220 (TVAGG3_0070080), sharing over 70% sequence identity with mouse *m*Arf6, likely retains a similar function (Fig. S3B). Specially, T31N and Q71L mutations in *Tv*Arf220 affected *Tv*PI4P5K membrane trafficking and PIP_2_ dynamics, suggesting that GTP-GDP exchange and GTPase activation are functional in *Tv*Arf220 regulation. In iron-depleted parasites, discrete cytoplasmic puncta of *Tv*Arf220 and *Tv*PI4P5K imply that their localization to vesicle-like compartments, though whether vesicle fusion precedes co-trafficking to the plasma membrane remains to be elucidated. Overexpression of Q71L increased *Tv*PI4P5K and PIP_2_ levels at the plasma membrane, with a slight rise in cytoplasmic PIP_2_, implying that dysregulated *Tv*Arf220 activity alters PIP_2_ distribution (Fig. S4D). Intriguingly, PIP_2_ replenishment in the T31N mutant recovered calcium levels and actin-related activities (Fig. 5C, 6B, 7A and C; Fig. S8B and C), speculating that *Tv*Arf220 activation is upstream of PIP_2_ signaling.

The switch I and II regions of Arf proteins form a structural core for interacting with cellular counterparts. While these regions are conserved, subtle conformational differences may arise due to variations in flanking amino acids (56). We speculate that *Tv*Arf220’s conformation may differ from *m*Arf1 or *m*Arf6, potentially interacting with distinct Guanine nucleotide exchange factors (GEFs) or GTPase-activating proteins (GAPs). Unlike *m*Arf6, which primarily localizes to the plasma membrane, *m*Arf1-GDP resides mostly in the cytoplasm and associates with plasma membrane via its N terminal region (56), a sequence longer in *Tv*Arf220 than in *m*Arf6. Whether *Tv*Arf220’s N-terminal sequence bridges its membrane localization remains to be determined.

### *Tv*PI4P5K turnover

The balance between protein synthesis and degradation is vital for cellular homeostasis, with the ubiquitin-proteasome and lysosomal pathways being the primary mechanisms mediating protein turnover (78). Our previous work demonstrated that iron-triggered ubiquitination is required for Myb3 nuclear translocation (20) and proteasomal degradation, with the latter process being prolonged by MG-132, supporting the presence of ubiquitination system in *T. vaginalis*. The decline in TvPI4P5K level post 60 min of iron repletion was stabilized by chloroquine but not by MG-132 (Fig. 2A and B, and S2A), suggesting that *Tv*PI4P5K turnover is manipulated via the lysosomal degradation pathway. In heart cells, chaperone-mediated autophagy involves the heat shock cognate protein 70 complex recognizing and translocating substrates to the lysosome through LAMP-2A (78). Coincidently, we identified *Tv*HSP70 in the *Tv*PI4P5K protein complex (Fig. S3A), raising the possibility of its role in *Tv*PI4P5K lysosomal proteostasis, a hypothesis currently under investigation.

### *Tv*Arf220-PIP_2_ signaling on cytotoxicity

*T. vaginalis* utilizes actin-based phagocytosis to damage and kill host epithelium cells (17, 57). High concentration of F-actin surrounded phagocytosed microspheres (Fig. 9D), and inhibiting F-actin with LatB or in *Tv*Arf220 T31N mutant significantly impaired parasite phagocytosis, underscoring the role of *Tv*Arf220 in actin-driven phagocytosis (Fig. 9E). Host uptake of parasite-secreted proteins (15, 24, 79) or extracellular vesicles (80–82) may also impact host-parasite interactions. Arf proteins and PIP_2_ can regulate exocytosis through distinct pathways (83, 84). In PIP_2_-depleted *Tv*Arf220 T31N mutant, the reduced contact-independent cytotoxic activity and extracellular vesicles can be restored by reloading PIP_2_ into the parasite (Fig. S8B and C). This supports that *Tv*Arf220 may mediate secretion indirectly via activating PIP_2_ signaling.

In conclusion, iron globally modulates *T. vaginalis* cytotoxic activities via *Tv*Arf220-PIP_2_-calcium signaling. Our findings may explain the exacerbation of trichomoniasis symptoms during menstruation and unveil a novel mechanism of *T. vaginalis* pathogenicity, offering a potential therapeutic target.

## MATERIALS AND METHODS

### Cell culture

*T. vaginalis* incubated in Trypticase-Yeast Extract-Iron (TYI) medium with 10% bovine serum at 37°C was defined as the normal-iron culture condition. The trophozoites cultured in TYI growth medium supplemented with 50 µM of 2,2′-dipyridyl or 250 µM of ferrous ammonium sulfate were iron-depleted or iron-repleted culture conditions, respectively (20). For the drug challenge, the parasites were cultured in a medium containing 10 µM of edelfosine (TargetMol), 20 µM of BAPTA-AM (Abcam), 20 µM of A23187 (Abcam), 5 µg/mL of Brefeldin A (TargetMol), or 120 µM of chloroquine at 37°C for 90 min. *h*VECs were cultured in keratinocyte serum-free medium (Gibco) at 37°C with 5% CO_2_.

### Plasmid construct

To construct the plasmid overexpressing HA-*Tv*PI4P5K and its kinase-deficient K136A mutant in *T. vaginalis*, the pFLP-HA-*Tv*PI4P5K and pFLP-HA-*Tv*PI4P5K (K136A) plasmids were constructed as described previously (27, 85). The target gene was driven by the promoter (−786 to +11, Data S1) of fibronectin-like protein-1 (FLP, TVAGG3_0826220) (86), and the selective *neo* gene was driven by the β-tubulin (TUB) proximal promoter (Fig. S3C).

The full-length *Tv*Arf220 (TVAGG3_0070080) DNA sequence was synthesized by a customized gene service (GenScript), as the template DNA for subsequent PCR. To construct the plasmid overexpressing *Tv*Arf220, the DNA fragment amplified from the synthesized template DNA by the primer pair *Tv*Arf220-BamHI-5′ and *Tv*Arf220-XhoI-3′ (Table 1) was subcloned into the BamHI/XhoI-restricted pFLP-HA-*Tv*PI4P5K (27) or pET28-His-*Tv*FACPα (26) vectors to obtain the pFLP-HA-*Tv*Arf220 or pET28-His-*Tv*Arf220 plasmids, respectively. T31N and Q71L were introduced into the pFLP-HA-*Tv*Arf220 plasmid using a site-directed mutagenesis kit (Toyobo) and the primer pairs *Tv*Arf220 (T31N)-5´/*Tv*Arf220 (T31N)-3´ and *Tv*Arf220 (Q71L)-5´/*Tv*Arf220 (Q71L)-3´ (Table 1) to obtain the pFLP-HA-*Tv*Arf220 (T31N) and pFLP-HA-*Tv*Arf220 (Q71L) plasmids, respectively (Fig. S3C).

**TABLE 1:** The oligonucleotide primers used in this study^*a*^

Primer	Sequence (5´ to 3´)
pFLP-HA-*Tv*PI4P5K plasmid	
*Tv*PI4P5K-BamHI-5´	AAGGATCCATGTCTCGCTCCGAATATAGTGA
*Tv*PI4P5K-XhoI-3´	AACTCGAGTTACTCTTGATCTTCAGATTTTG
pFLP-HA-*Tv*PI4P5K (K136A) plasmid	
*Tv*PI4P5K (K136A)-5´	*GCT*ACTCAAACGAAAGATGAAATGAAA
*Tv*PI4P5K (K136A)-3´	AAT AACATATCGACCGTCCCAAGT
pFLP-HA-*Tv*Arf220 plasmid	
*Tv*Arf220-BamHI-5´	AAGGATCC ATG GGT CTC TTA TTC AGT GAA ACA
*Tv*Arf220-XhoI-3´	AACTCGAG TTA GAA GTG CTG GTT GAT CTG ATC
pFLP-HA-*Tv*Arf220 (T31N) plasmid	
*Tv*Arf220 (T31N)-5´	*AAC* ACA GTC CTT TAT AAG CTT AAG CTT
*Tv*Arf220 (T31N)-3´	CTT GCC TGC AGC ATC GAG ACC GAG
pFLP-HA-*Tv*Arf220 (Q71L) plasmid	
*Tv*Arf220 (Q71L)-5´	*CTT* GAC AGA ATC CGT GCT CTC TGG CGC
*Tv*Arf220 (Q71L)-3´	GCC ACC GAC ATC CCA AAC ATT CAT

^*a*^ The sequences of the restriction enzymes are underlined and mutations are italicized.

The plasmid DNA was electroporated into *T. vaginalis* trophozoites by a DNA delivery system (BTX). The transgenic trophozoites were selected by paromomycin in a start concentration of 100 µg/mL to obtain various stable transfectant clones.

### Recombinant protein production

The plasmid was transformed into *Escherichia coli* (BL21) for recombinant protein production as previously described (20, 26, 85). Briefly, *E. coli* culture at OD_260_ 0.6 was induced with 1 mM Isopropyl β-D-1-thiogalactopyranoside (IPTG) and then incubated at 30°C for 3 h. The bacteria were recovered by 3,000 × *g* centrifugation and washed once with Phosphate buffered saline (PBS), followed by sonication. The bacterial lysate was centrifuged at 15,000 × *g* at 4°C for 15 min to remove cell debris and insoluble proteins before purification using a 1 mL Ni-NTA column as suggested by the supplier (Qiagen).

### Antibody production

The recombinant full-length His-*Tv*Arf220 protein was produced and purified according to a standard protocol as described earlier (20, 26, 85). The use of the purified His-*Tv*Arf220 protein to produce antibodies is a customized service provided by the manufacturer (Genetex, CA, USA). The anti-*Tv*Arf220 antibody specificity was tested by western blotting, as shown in Fig. S3D.

### Calcium Green intracellular calcium detection

The parasites were incubated in PBS with 1% Bovine serum albumin (BSA) and 5 µM Calcium Green (Invitrogen) at 37°C for 20 min. After washing with 1 mL PBS to remove the excess dye, the parasites were fixed with 4% formaldehyde and attached to the poly-L-lysine-coated glass slide. After washing unattached parasites with PBS, the sample was air-dried and mounted in an anti-fade medium with DAPI before imaging by confocal microscopy (Zeiss, LSM-780) with an excitation wavelength at 506 nm and an emission wavelength at 531 nm. The relative signal intensity averaged from 150 trophozoites in five independent microscopic fields was measured by ImageJ v.1.53k software (National Institutes of Health).

### Immunofluorescence assay

For PIP_2_ detection, the parasite trophozoites were fixed with 4% formaldehyde and permeabilized with 0.5% saponin in Tris-buffered saline (TBS). After triple washes with TBS, the samples were incubated in TBS with 10% goat serum at 37°C for 20 min. For the other conventional antibody detection, the fixed trophozoites were permeabilized with 0.2% Triton X-100, omitting the goat serum blocking procedure. After three washes with TBS, the sample was reacted with primary mouse anti-PIP_2_ (1:400, Echelon Biosciences), rat anti-*Tv*Arf220 (1:500), and rabbit anti-*Tv*PI4P5K (1:400) (27) antibodies diluted in TBS containing 1% BSA at 4°C overnight. Next, the sample was washed three times with TBS and incubated with goat anti-mouse IgM or IgG secondary antibodies conjugated with FITC or Cy3 (1:200, Jackson ImmunoResearch) in TBS containing 1% BSA at 37°C for 1 h. The sample was washed three times with TBS and then air-dried at room temperature for 20 min. To determine colocalization of lysosome and *Tv*PI4P5K puncta, the trophozoites incubated in the medium containing 200 nM LysoTracker Red DND-99 (ThermoFisher Scientific) for 30 min were fixed for co-staining with anti-*Tv*PI4P5K antibody (1:800). The sample mounted in an anti-fade medium containing DAPI (Vector Labs) was observed by a confocal microscope (Objective: Plan-Apochromat 100/1.40 Oil Ph3, LSM-780, Zeiss). The fluorescent signal was captured in a single Z-slice to construct the final image. Our observations were conducted on over 150 trophozoites across five independent microscopic fields.

### Colocalization assay

The cellular distribution of fluorescence signal from a single Z-slice confocal image was analyzed by the plot profile function in ImageJ v.1.53k software (National Institutes of Health) and analyzed using Microsoft Excel 2019 to calculate the Pearson correlation coefficient (PCC). The PCC was measured from 30 trophozoites of three independent microscopic fields, with averages calculated from three biological replicates. Pearson’s value of +1 indicates a positive correlation, −1 indicates a negative correlation, and 0 indicates no correlation. Alternatively, the confocal images were converted to 2.5D view images by ZEN software (Zeiss), converting the dual-color signal intensities in the two-dimensional image into a height map represented by the extension in Z-direction to highlight the colocalized signal around the plasma membrane.

### Dot blot assay

Approximately 1 × 10^7^ trophozoites were vigorously vortexed in 200 µL of lysis buffer (1% Triton X-100, 100 µg/mL TLCK, 1× protease inhibitor cocktail, 1× phosphatase inhibitor cocktail, 5 mM EDTA in TBS) at 4°C for 20 min to prepare the total protein lysate. The protein lysates were serially diluted and blotted on the nitrocellulose (NC) membrane (ThermoFisher Scientific) in a 96-well microfiltration apparatus (BIO-RAD). The NC membrane was incubated in blocking buffer (5% nonfat milk in TBS with 0.1% Tween-20) with gentle agitation at 37°C for 1 h. After triple washes with TBS containing 0.1% Tween-20 (TBST), the membrane was incubated with the primary mouse anti-PIP_2_ (1:4,000, Echelon Biosciences) or mouse anti-α-tubulin (1:5,000, Sigma Aldrich, DM-1A) antibodies at 4°C overnight. After washing three times with TBST, the membrane was incubated with Horseradish peroxidase (HRP)-conjugated goat anti-mouse IgM or IgG secondary antibodies at 37°C for 1 h. The signal was detected by an enhanced chemiluminescence substrate (ThermoFisher Scientific) and imaged using a UVP imaging system (Analytik Jena Company).

### Western blotting

The denatured protein sample was separated by sodium dodecyl sulfate polyacrylamide gel electrophoresis (SDS-PAGE) in a 12% gel and transferred onto the polyvinylidene difluoride membrane by a tank blotting system (BIO-RAD). The membrane was incubated in the blocking buffer (5% nonfat milk in TBST) at 37°C for 1 h and then incubated with primary antibodies, mouse anti-HA (1:1,000, Sigma-Aldrich), rabbit anti-*Tv*PI4P5K (1:3,000) (27), mouse anti-α-actin (1:10,000, Genetex), rat anti-*Tv*HSP70 (1:20,000), rat anti-*Tv*Arf220 (1:3,000), rat anti-*Tv*PKAc (1:1,000) (20), mouse anti-*Tv*CyP2 (1:2,000) (85), rat anti-*Tv*Gα1 (1:1,000) (85), and mouse anti-α-tubulin (1:10,000, Sigma-Aldrich) diluted in blocking buffer, at 4°C overnight. After three washes with TBST, the membrane was incubated with HRP-conjugated goat anti-mouse, -rat, or -rabbit IgG secondary antibody (1:5,000, Jackson ImmunoResearch) in the blocking buffer at 37°C for 1 h. For Fig. 3C, the signals for *Tv*PI4P5K in immunoprecipitants and α-tubulin in total lysates were detected using a membrane transferred from two separate gels. The upper gel, resolving immunoprecipitants, preserved the 63 ~ 170 kDa range for *Tv*PI4P5K detection, while the lower gel, resolving total lysates, retained the region 43 ~ 63 kDa for α-tubulin detection. The membrane was sequentially incubated with the mouse anti-α-tubulin, goat anti-mouse-IgG-HRP, rabbit anti-*Tv*PI4P5K, and goat anti-rabbit IgG antibodies at earlier described dilutions. Signals were detected by enhanced chemiluminescence substrate (ThermoFisher Scientific) and captured on a UVP imaging system (Analytik Jena Company).

### Immunoprecipitation

Approximately 3 × 10^7^ trophozoites were lysed in 1 mL of lysis buffer (1% Triton X-100, 100 µg/mL TLCK, 1× Protease inhibitor cocktail, 1× phosphatase inhibitor cocktail, 5 mM EDTA in TBS) with a vigorous vortex at 4°C for 30 min and centrifuged at 23,000 × *g* to recover the soluble protein lysate. Twenty microliters of agarose beads conjugated with anti-HA antibody (Sigma-Aldrich) was added to 1 mL soluble lysate (1 mg/mL) for incubation overnight at 4°C. After washing the beads three times with lysis buffer, the beads were recovered by 2,000 × *g* centrifugation and denatured in 1× SDS sample buffer for western blotting. The IgG heavy chain was detected as the antibody loading control for immunoprecipitation.

### Mass spectrometry

The protein sample denatured in 1× SDS sample buffer was separated by SDS PAGE in a 10% gel, and the protein bands were visualized by SYPRO Ruby Protein Gel Stain (ThermoFisher Scientific). The exercised gel pieces were sequentially denatured in 20 mM Dithiothreitol (DTT) at 55°C for 30 min, alkylated in 55 mM iodoacetamide at room temperature in the dark for 1 h, and digested in gel with trypsin versus protein in a ratio of 1:100 at 37°C overnight. The tryptic peptides were extracted from the gel for mass spectrometry identification as previously described (20).

### *In vivo* G-actin/ F-actin fractionation

A commercial *in vivo* assay biochemical kit (Cytoskeleton Inc.) was used to fractionate G- and F-actin, according to the operating manual, with minor modifications. Approximately 3 × 10^7^ trophozoites were incubated in cell lysis buffer (Cytoskeleton Inc) with vigorous agitation at 4°C for 30 min and homogenized using a 23-gauge needle on a 5 mL syringe. Next, the total lysate was centrifuged at 1,000 × *g* to remove the cell debris, followed by ultracentrifugation at 100,000 × *g* for 1 h to recover the insoluble F-actin and associated proteins in the pellet from soluble G-actin in the supernatant. In western blotting, α-tubulin and *Tv*CyP2 were detected as purity markers for the supernatant and pellet fractions, respectively. The α-actin signal intensity in the supernatant and pellet fractions were first normalized to *Tv*CyP2 and α-tubulin, respectively, and then the α-actin signal ratio between supernatant and pellet fractions was calculated to evaluate actin assembly.

### Morphogenesis analysis

Trophozoites were cultured in a T25 flask at 37°C for 1 h, and the parasite morphology was observed by a phase-contrast microscope (Olympus, CKX31). The flagellate trophozoites exhibited a solid spherical morphology with a bright and clear cell edge and a diameter under 10 µm. In contrast, the amoeboid forms, typically exceeding 10 µm in diameter, appeared irregular or flattened, round disk-like shapes with fine cell edges upon adhesion to the glass surface. The proportions of flagellate and amoeboid forms were quantified by counting 300 trophozoites across five random microscopic fields.

### Cytoadherence assay

The CFSE (ThermoFisher Scientific, C34554)-labeled parasites were co-cultured with *h*VECs at MOI of 2 in a minimal thin layer of culture medium for 1 h under the atmosphere with 5% CO_2_. After washing away the unbound trophozoites with pre-warmed TYI medium, the specimen was fixed with 4% formaldehyde, and the CFSE signal was detected by inverted fluorescence microscopy (CFSE Ex/Em = 492/517) (Axiovert 200M, Zeiss). For each assay, the signal intensities quantified by ImageJ v.1.53k software (National Institutes of Health) in five independent microscopic fields were averaged to evaluate the relative parasite cytoadherence of conditional samples. The signal intensity from the control parasites was defined as the basal level of 100%.

### Cytolysis by fluorescence microscopy

The Orange-CMRA (ThermoFisher Scientific, C34551)-labeled parasites and *h*VECs were co-cultured at MOI of 4 on a coverslip placed in a culture microplate. The microplate was centrifuged at 200 × *g* for 5 min to sediment trophozoites for contact with the *h*VECs monolayer and then incubated for 1 h under the atmosphere with 5% CO_2_. The specimens were fixed with 4% formaldehyde, then permeabilized with PBS containing 0.2% Triton X-100 and stained with FITC-conjugated phalloidin (1:1,000, Abcam, ab235137) at room temperature for 1 h. The samples were washed three times with PBS and air-dried for 20 min. The coverslips were mounted in an anti-fade medium with DAPI and inverted on a glass slide for observation by confocal microscopy (FITC Ex/Em = 492/518, Orange-CMRA Ex/Em = 548/576) (LSM700, Zeiss). The clear lytic area was measured by AxioVision software (Rel.4.8, Zeiss). For each assay, the lysis area per trophozoite was averaged from five independent microscopic fields to evaluate parasite cytolysis activity.

### LDH cytotoxicity assay

The feasibility of using a co-culture of *T. vaginalis* and host cells for the LDH cytotoxicity assay was confirmed (27). Briefly, the trophozoites were inoculated into a 96-well microplate containing host cells at 90% confluence and incubated under the atmosphere with 5% CO_2_. The medium supernatants collected at 4 h post-infection were centrifuged at 1,000 × *g* to remove intracellular LDH of cells or parasites before cytotoxicity assay (Biochain). One hundred microliters of test sample was mixed with 45 µL of assay mixture for 30 min at room temperature. After adding 50 µL of stop solution, the sample colorimetric signal was detected at OD_490_ by a spectrophotometer (SpectraMax190, Molecular Devices). In each assay, the samples from parasite-free host cells with or without lysis solution treatment were detected as high- and low-level controls, respectively. The cytotoxicity (%) was measured as follows: $\frac{OD490\;\left(test\;sample\right)-OD490\;\left(low-level\;control\right)}{OD490\;\left(high-level\;control\right)-OD490\;\left(low-level\;control\right)}\times 100.$

### 3-(4,5-Dimethylthiazol-2-yl)-2,5-diphenyltetrazolium bromide (MTT) cell viability assay

The host cell viability in a transwell platform was evaluated by an MTT cell viability assay kit (Biochain). The trophozoites at a specific MOI were inoculated into the top inserts with 0.4 µm porous membrane, then placed on the 24-well transwell microplate confluent with *h*VECs monolayer in 320 µL of culture medium. After culturing for 4 h under the atmosphere with 5% CO_2_, 60 µL of reaction reagent was added per well and incubated at 37°C for 4 h, followed by the addition of 320 µL of solubilizer reagent per well for 1 h at room temperature. The colorimetric signal was detected at OD_570_ by a spectrophotometer. For each assay, the medium only was detected as the blank control, and the parasite-free host cell cultures with and without 0.1% saponin were the low-level and high-level controls, respectively. The cell viability (%) was measured as follows: $\frac{OD570\;\left(test\;sample\right)-OD570\;\left(low-level\;control\right)}{OD570\;\left(high-level\;control\right)-OD570\;\left(low-level\;control\right)}\times 100.$

### Phagocytosis assay

To generate IgG-coated polystyrene beads, 0.5 mL of latex beads suspension (10%, 1.1 µm particle size, Sigma-Aldrich) was washed with 1 mL PBS and then incubated with 2 mg/mL human IgG on rotation at 37°C for 1 h. The beads were washed three times with PBS and suspended in 0.5 mL TYI culture medium before the phagocytosis assay (87). To assess phagocytic efficiency, *T. vaginalis* trophozoites were cultured on a polylysine-coated glass slide, and phagocytosis was initiated by adding 5 µL of IgG-opsonized latex beads suspension to 20 µL of parasite culture. The slide was centrifuged at 300 × *g* for 1 min to sediment beads and incubated at 37°C for 2 h under atmosphere with 5% CO_2_. Next, the samples were fixed with 4% followed by 0.1% Triton X-100 permeation at room temperature and double-stained with FITC-conjugated goat anti-human IgG (200×, Jackson ImmunoResearch) and TRITC-conjugated phalloidin (Sigma-Aldrich) at 37°C for 1 h. The images of phagocytosed microspheres were captured by confocal microscopy (LSM-780, Zeiss), and the number of phagocytosed beads was measured in 150 random parasites from three biological repeats to evaluate the phagocytosis efficiency.

### PIP_2_ intracellular delivery

The parasite culture was incubated with a combination of 30 µM diC16 PIP_2_ and 30 µM histone H1 phosphoinositides shuttle carrier at 37°C for 30 min to ensure the intracellular recovery of PIP_2_ (Echelon Biosciences Inc) (88).

### *Tv*Arf220-GTP binding assay

One milligram of *T. vaginalis* soluble lysate was incubated with 10 µg of GST-GGA3-PBD-conjugated on the resin (Cytoskeleton, Inc.) at rotation under 4°C for 2 h. The beads were washed with 1 mL of lysis buffer (1% Triton X-100, 100 µg/mL TLCK, 1× protease inhibitor cocktail, 1× phosphatase inhibitor cocktail, 5 mM EDTA in TBS) twice and then denatured in a 1× SDS sample buffer for SDS-PAGE or western blotting detection.

### Scanning electron microscopy

Samples were prepared as described (89). Briefly, trophozoites were co-incubated with *h*VECs on a coverslip and fixed at intervals with 4% formaldehyde and 2.5% glutaraldehyde in 0.1 M sodium phosphate buffer overnight. After washing twice in sodium phosphate buffer, samples were sequentially dehydrated in an ethanol gradient from 30% to 100%. The samples were immersed in liquid carbon dioxide before critical point drying and gold coating, then observed by an environmental scanning electron microscope (FEI Quanta 200).

### Subcellular fractionation by differential and gradient centrifugation

Organelle fractions were purified from 250 mL of cells using differential and gradient centrifugation with modifications (27, 85). The postnuclear lysate was fractionated into crude membrane fractions (P15 and P100) and the soluble S100 fraction. The P100 pellet was re-suspended in 0.5 mL of TBS by sonication, mixed with 0.1 mL OptiPrep, and layered onto the top of 12%–30% OptiPrep gradient with 2% step-wise increases. After ultracentrifugation at 353,000 × *g* at 4°C for 4 h (Beckman SW60 rotor), the samples were sequentially fractionated into 200 µL aliquots from the top of the gradient for western blotting analysis.

### Trypan blue exclusion assay

The trophozoites were stained with 0.4% trypan blue in PBS to evaluate the viability of the parasites. The percentage of viable cells was calculated from 300 parasites within five independent microscopic fields as follows: $\frac{Number\;of\;total\;trophozoites\;-\;Number\;of\;blue\;trophozoites}{Number\;of\;total\;trophozoites}\times 100.$

### Statistical analysis

The data were analyzed using Microsoft Office Excel 2019 software with Student’s *t*-test. A *P* < 0.05 was considered as a significant difference.

## Data Availability

All relevant data are within the manuscript and its supporting information files.

## SUPPLEMENTAL MATERIAL

Data S1 mbio.01864-24-s0001.pdf FLP promoter sequence -786 to +11.

Data S2 mbio.01864-24-s0002.xlsx Raw data of statistic analysis.

Data S3 mbio.01864-24-s0003.pdf Raw data of gels and blots.

Supplemental figures mbio.01864-24-s0004.docx Fig. S1 to S9.

Supplemental captions mbio.01864-24-s0005.docx Captions for Data S1, S2, and S3.

ASM does not own the copyrights to Supplemental Material that may be linked to, or accessed through, an article. The authors have granted ASM a non-exclusive, world-wide license to publish the Supplemental Material files. Please contact the corresponding author directly for reuse.
