# Supplementary material for: Iron triggers TvPI4P5K proteostasis and Arf-mediated cell membrane trafficking to regulate PIP2 signaling crucial for multiple pathogenic activities of the parasitic protozoan Trichomonas vaginalis
Source: mBio. 2024 Dec 23;16(2):e01864-24. doi: 10.1128/mbio.01864-24 (PMC11796385; doi:10.1128/mbio.01864-24)
Supplement: Data S1 — FLP promoter sequence -786 to +11. [file mbio.01864-24-s0001.pdf]

-786

ATTAATTAAGTTCAATACTCCATTTTATTGGATACATTCTAATTCAGCTGAACTAAATAGAAAATGATT

TGACAATAGATTGGAAACTTTTCATTCCTTATTCAAGAAAAAAAATATTTTTTTTGAAGAAAAATTCAA

TCGAAATTAAAATTTACACTTTTAGTGTGGTCGCATTGTCGCTTCGCTCGAATGCGACCACACTAAAAG

TGTAATTATCTAATTTATGCAAATGAGTCAAAAATTTTACTGTGTATGATTAGATTTCCTTTCAAAAAA

ATTGAATATAACACAAAAAGCTAATGGAAGCGTAAGGCGACTTCTTCCCACAGGCCAGGAATTCAATAT

TGAAACCGAATCCAACACATTTGTTGCAGTTTACTTCAAACAGCAAGGATATACCAGCGTTTATTACAC

CATAATTGAAGATTTCAAATCTACATTTCCCAATAAATTAACGTAGGAACCCAATTTCCACGAGTTAGA

TGCTTAGTAAACTCGTTACCCACTACGAGATATCCTTTCTATTGTTAGAATAAACCATTATTTATCATA

TTTCGAATTCAATTTTTTTGAATGGAAAATAATCGTATGCTATAAAATTTTTCCATTTGCCATGTTACA

TAATTGTTATTTAATGCATCCTTATTCTGATTAATATATTTGGGTTGAAAAAATCGGTTACCGATTGGA

TTGAATTCGTTGATCATGATGGGTAAGGAAATTTTTCAAAAGTGACATATGATCAAAATTTTTGAGAGC

+1

CTTTGAATAAAATAAAATTTTCATATTC**A**CTTTT**ATG**GC

Inr

Inr: Transcription initiator
