# Supplementary material for: Iron triggers TvPI4P5K proteostasis and Arf-mediated cell membrane trafficking to regulate PIP2 signaling crucial for multiple pathogenic activities of the parasitic protozoan Trichomonas vaginalis
Source: mBio. 2024 Dec 23;16(2):e01864-24. doi: 10.1128/mbio.01864-24 (PMC11796385; doi:10.1128/mbio.01864-24)
Supplement: Data S3 — Raw data of gels and blots. [file mbio.01864-24-s0003.pdf]

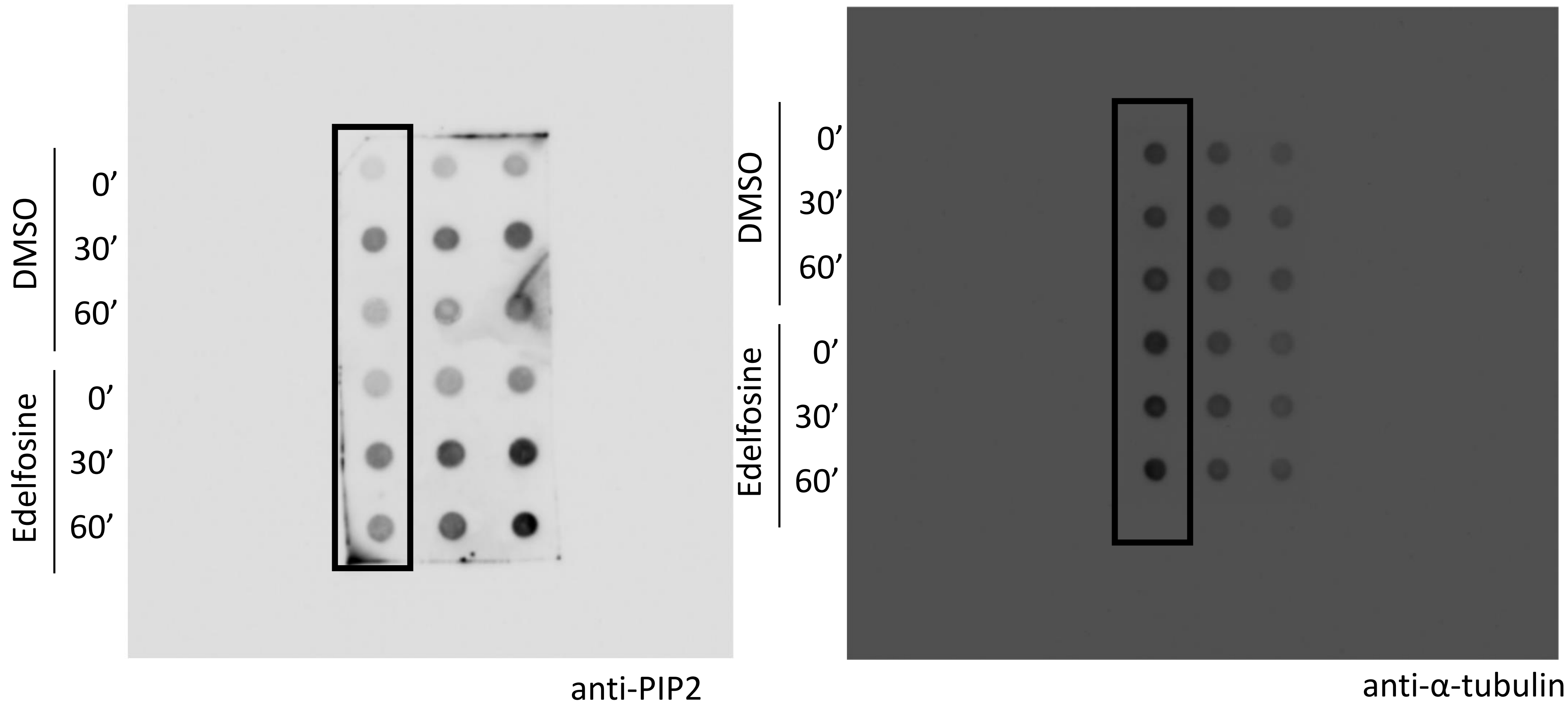

Fig1B. The raw data of dot blot assay. The boxed regions were shown in this article.

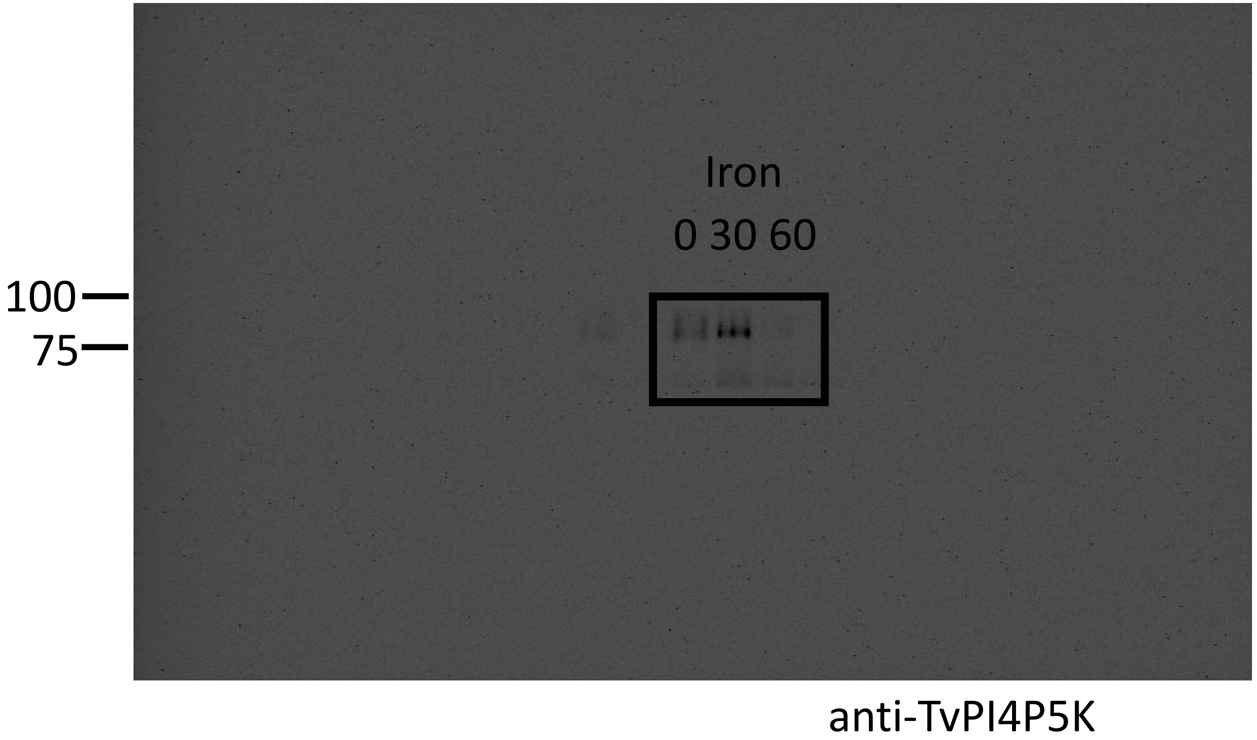

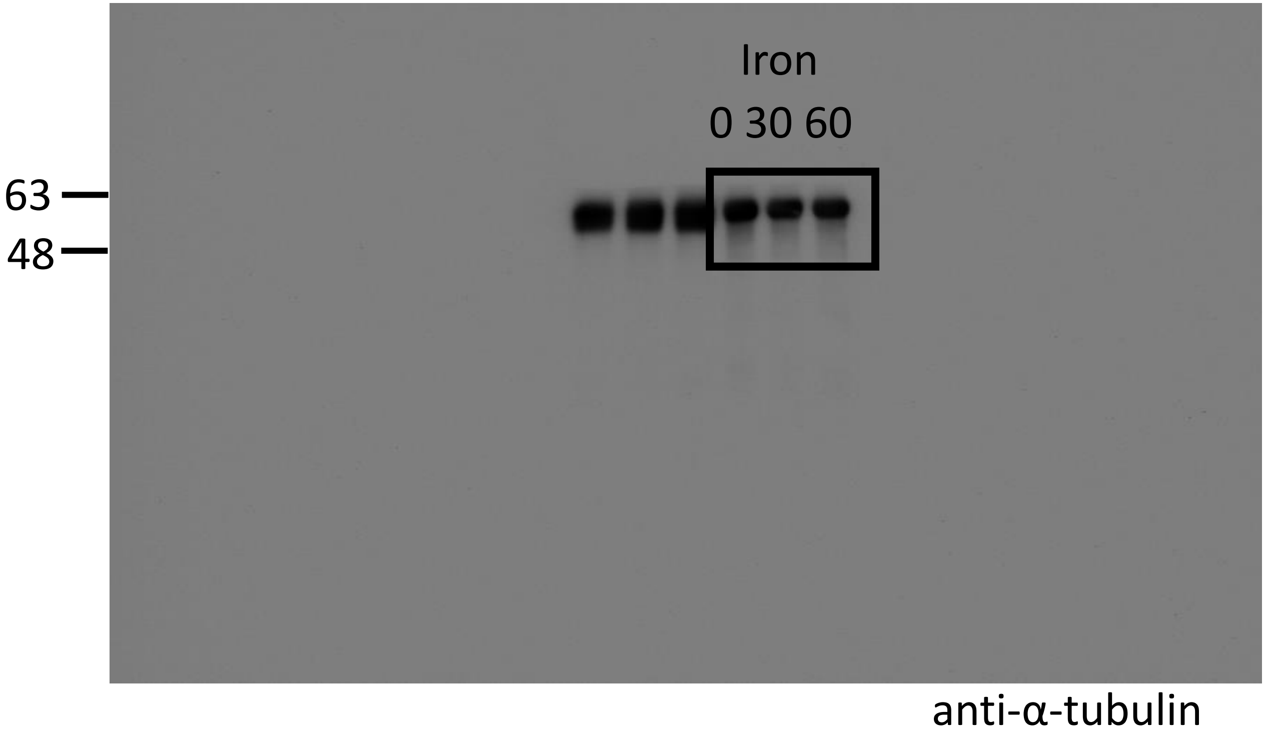

Fig1C. The raw data of western blotting. The boxed regions were shown in this article.

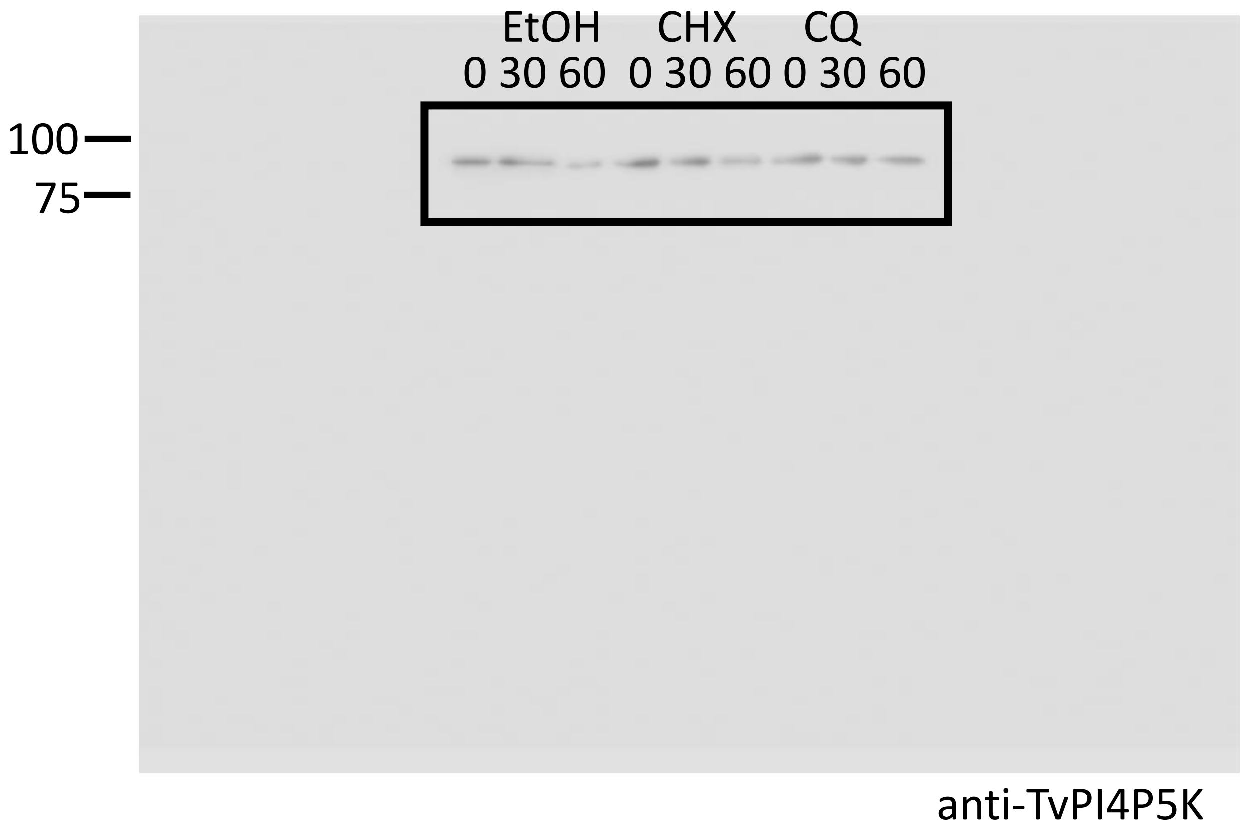

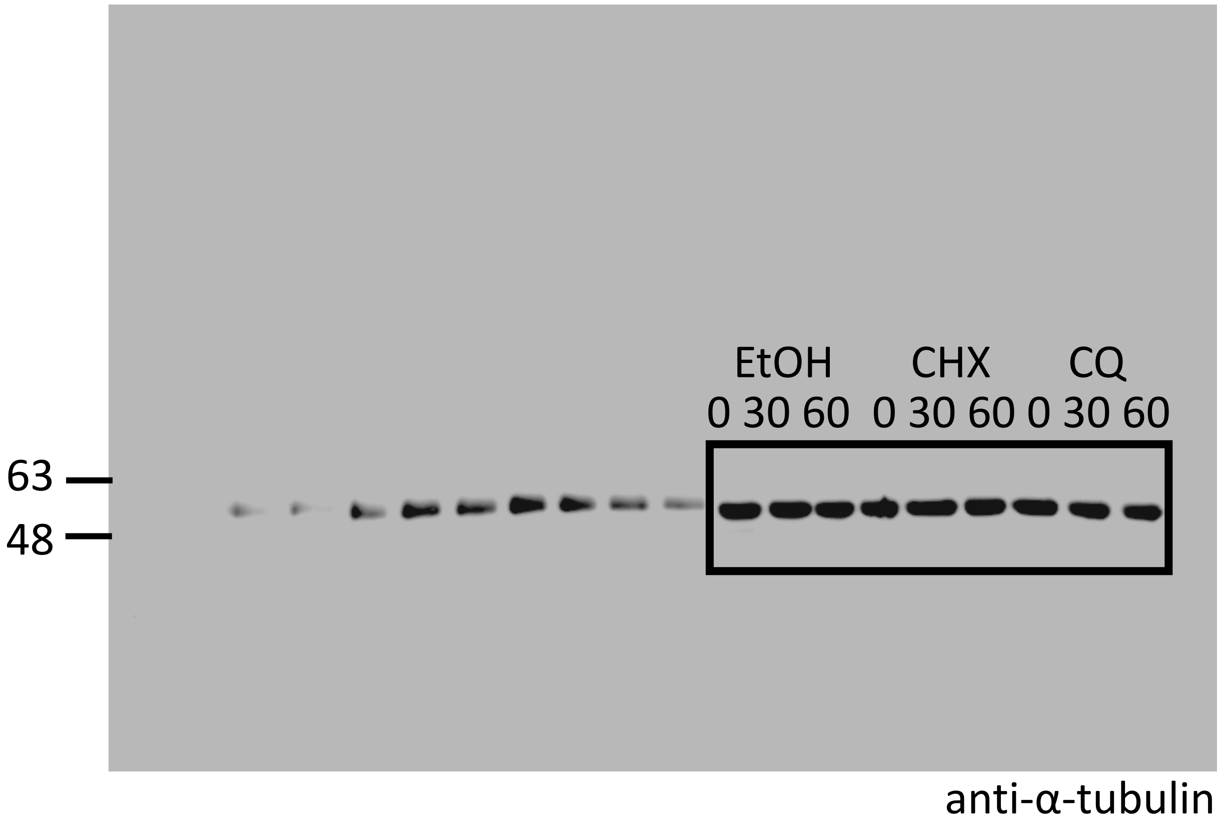

Fig2A. The raw data of western blotting. The boxed regions were shown in this article.

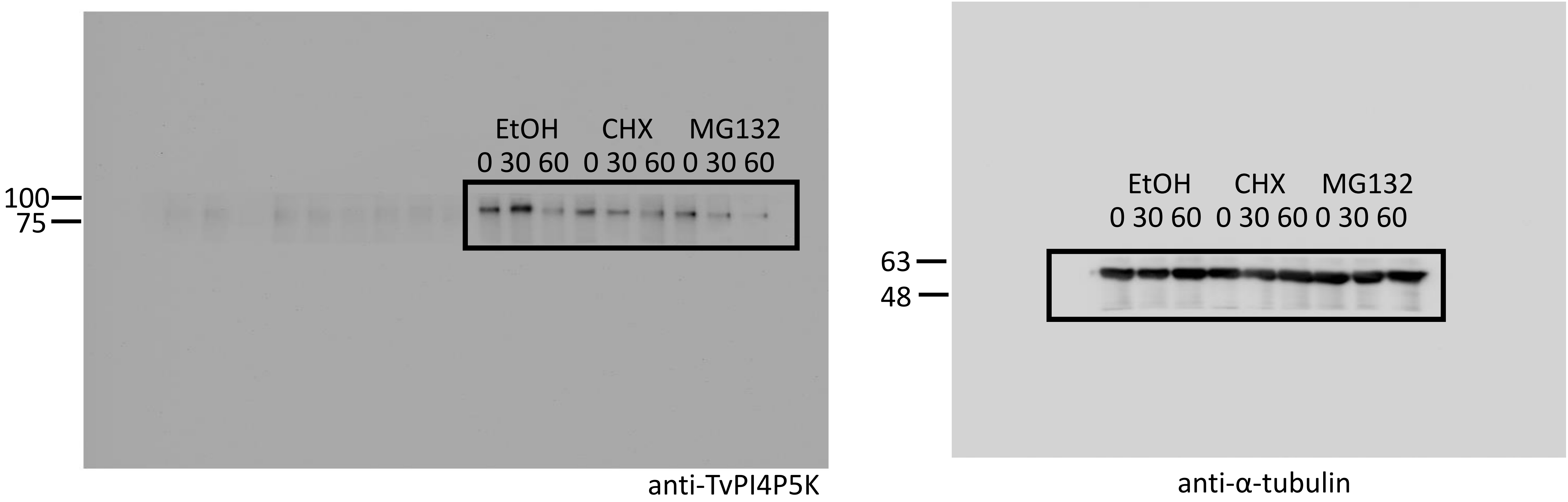

Fig2B. The raw data of western blotting. The boxed regions were shown in the article

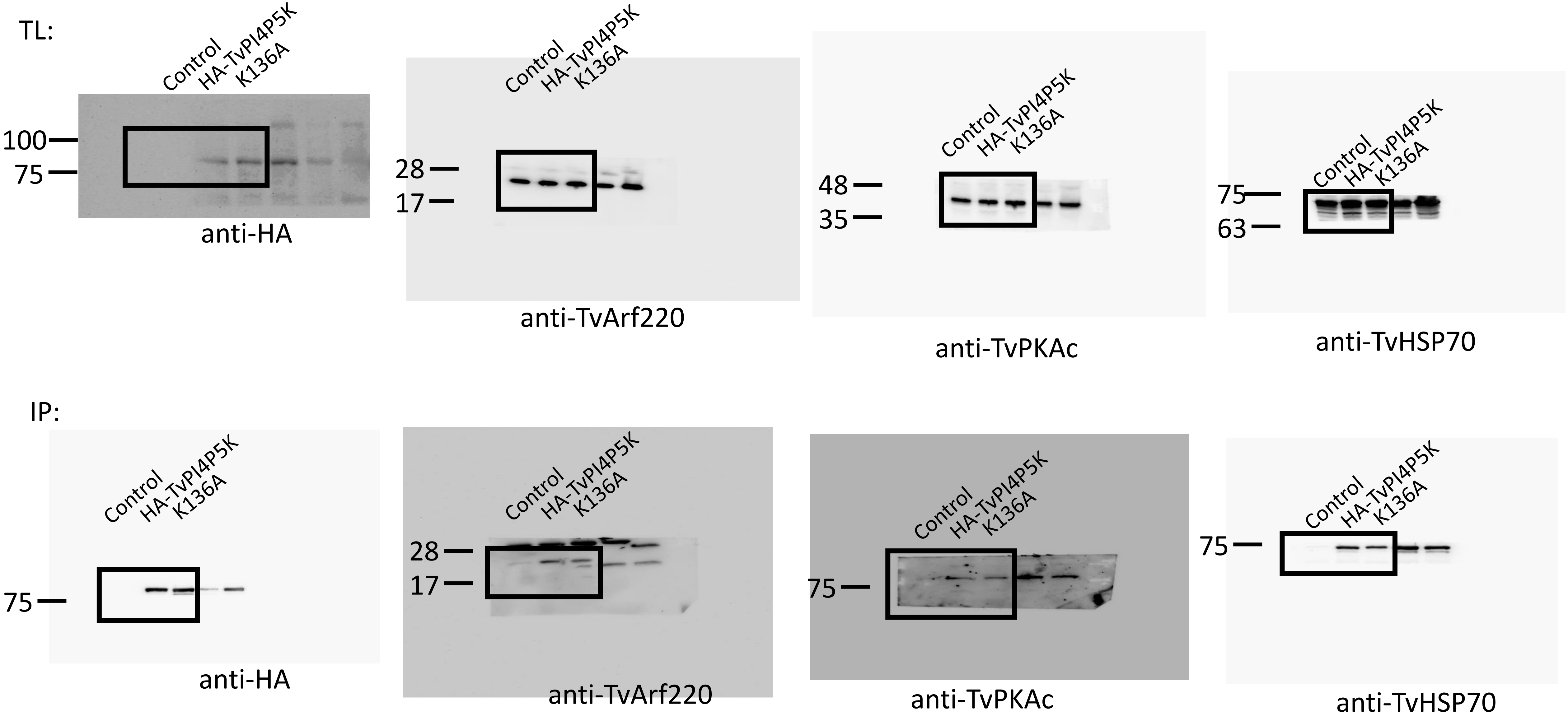

Fig3A. The raw data of western blotting.

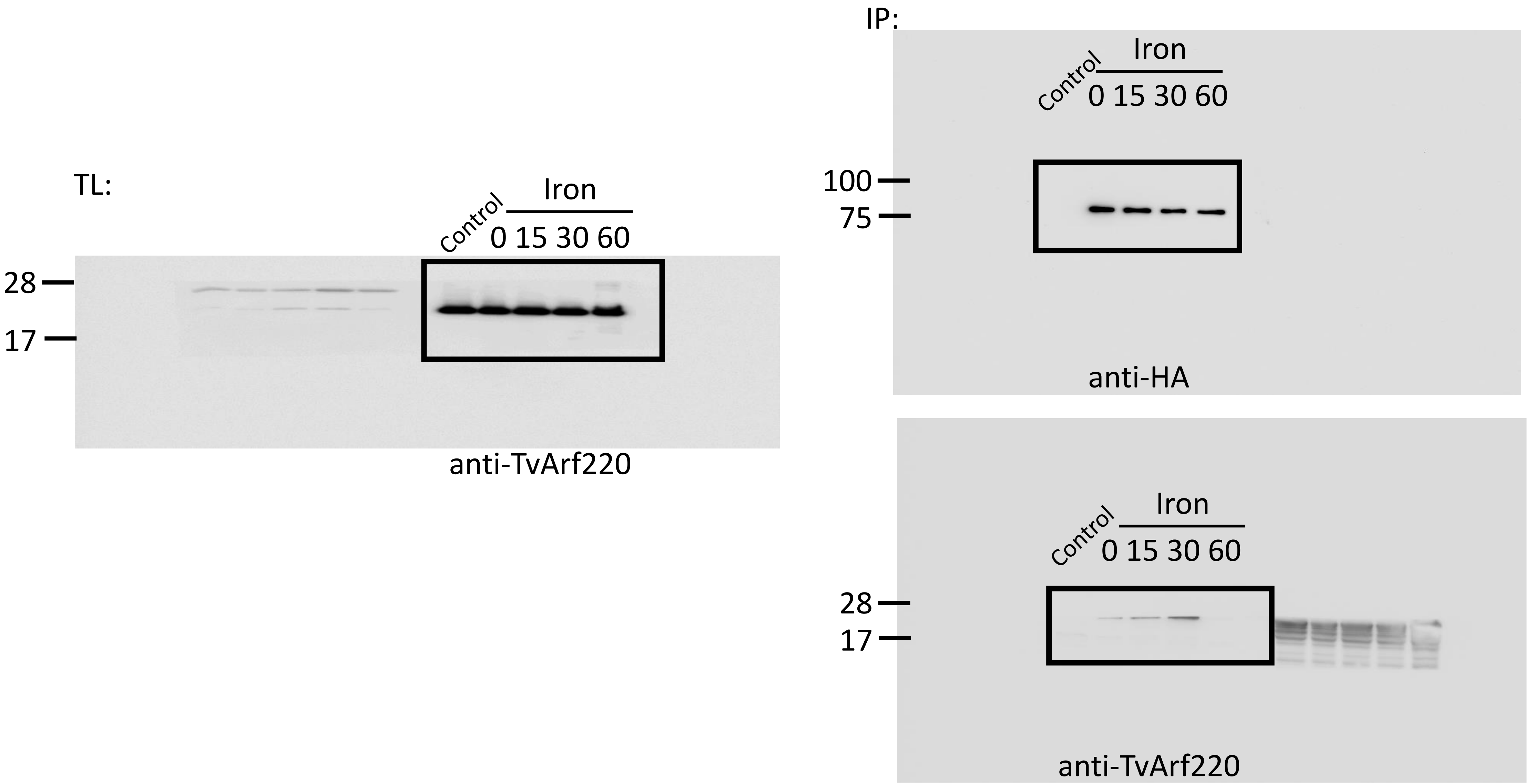

Fig3B. The raw data of western blotting.

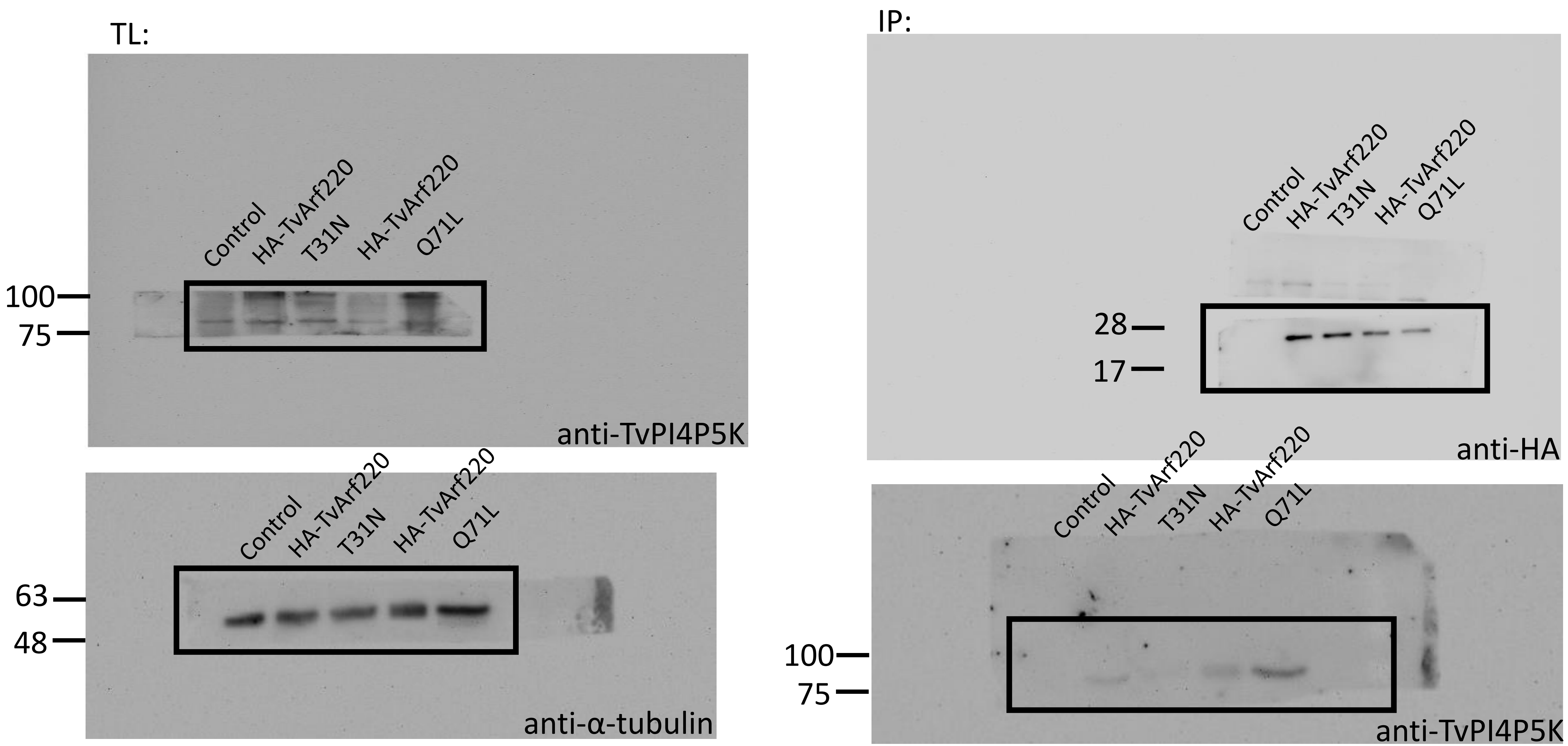

Fig3C. The raw data of western blotting. The boxed regions were shown in this article.

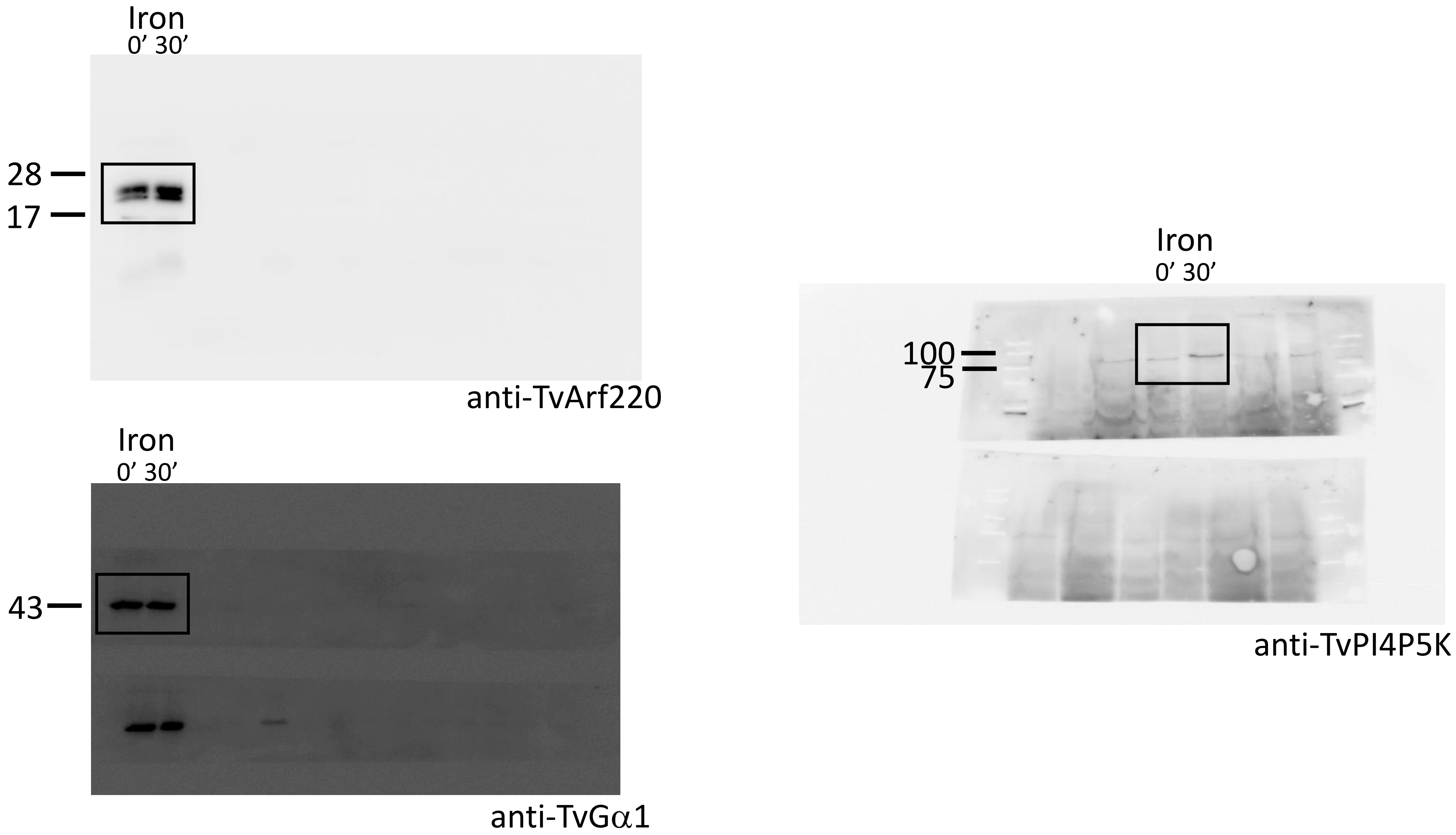

Fig3F. The raw data of western blotting. The boxed regions were shown in this article.

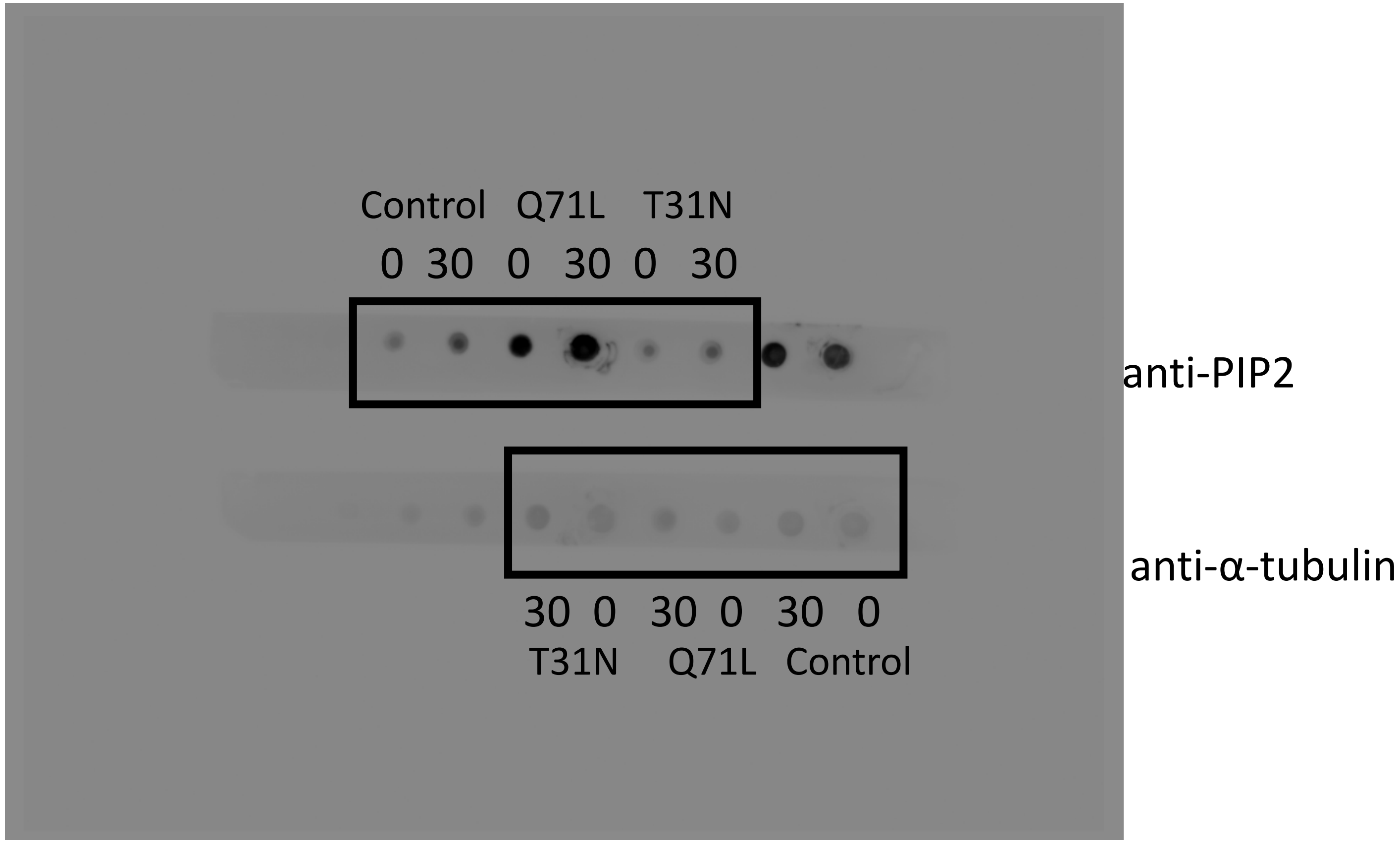

Fig4D. The raw data of bot blot assay. The boxed regions were shown in this article.

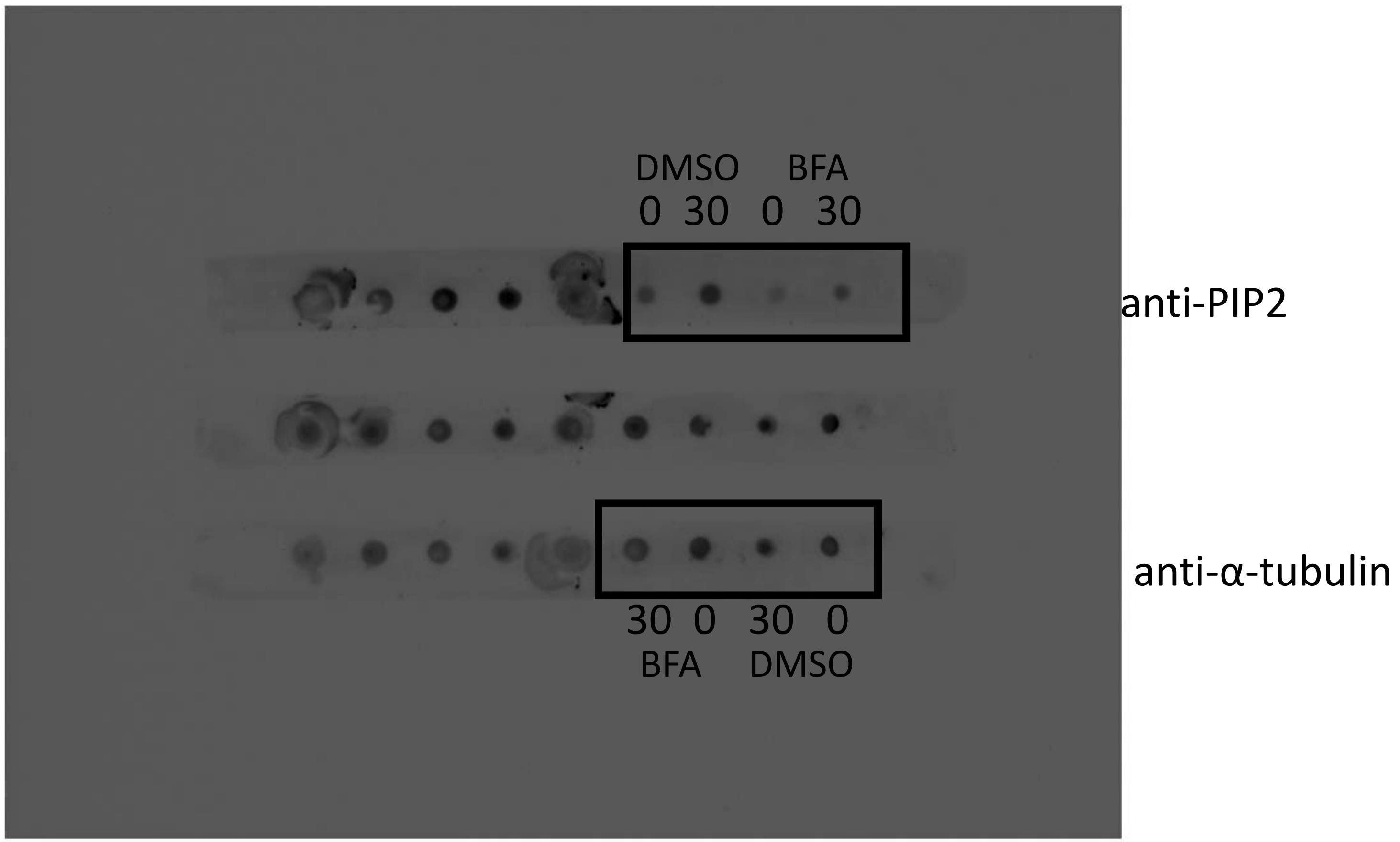

Fig4E. The raw data of dot blot assay. The boxed regions were shown in this article.

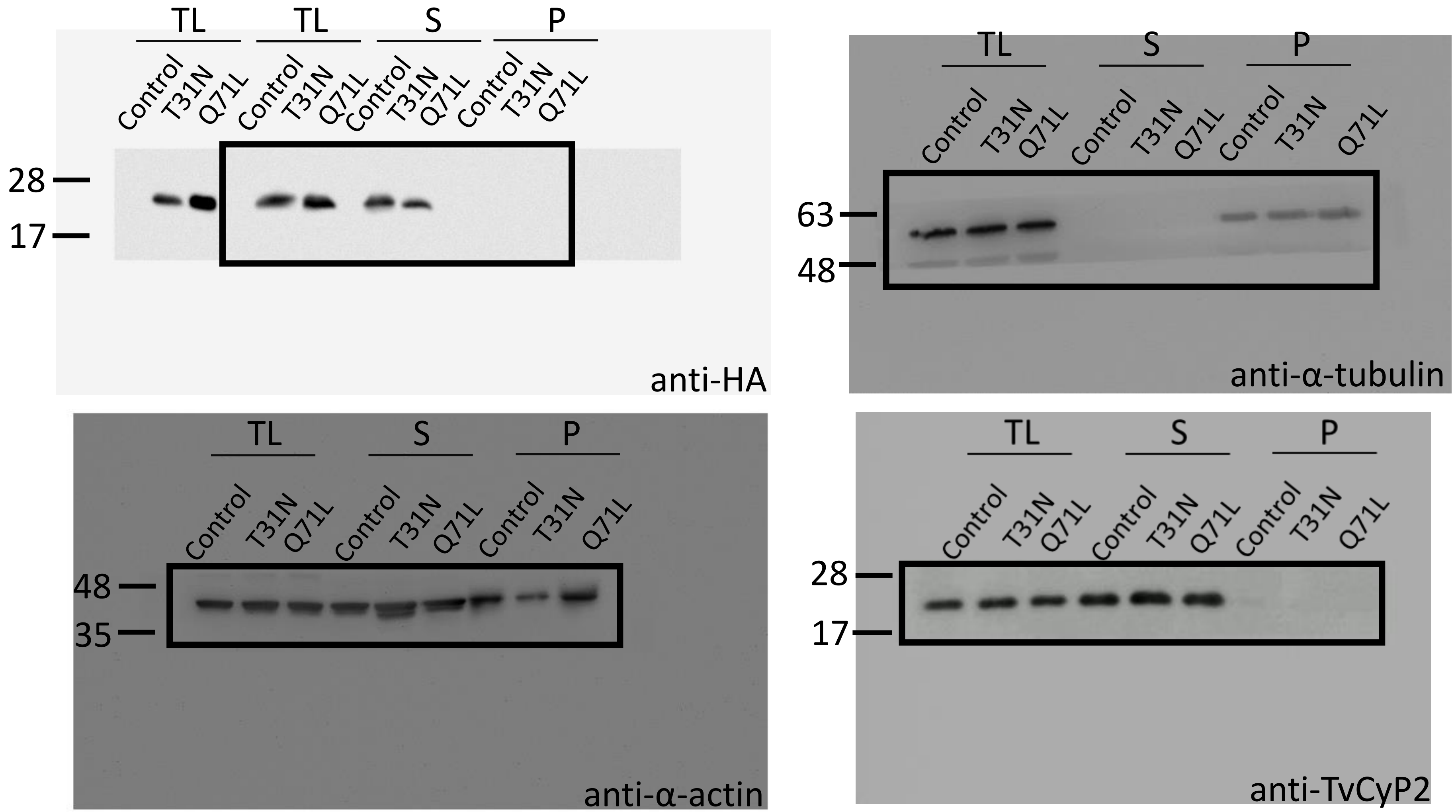

Fig6A. The raw data of western blotting. The boxed regions were shown in this article.

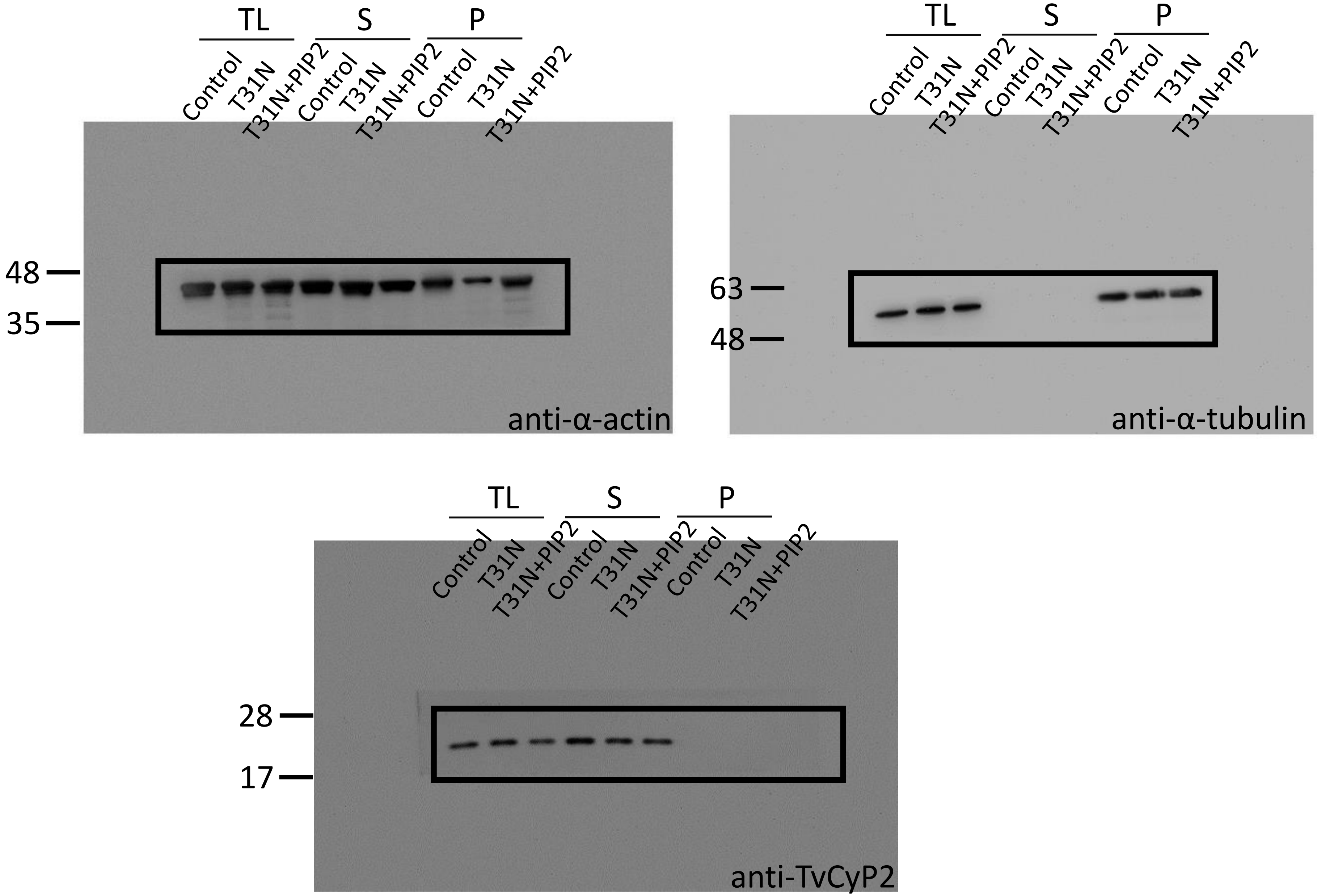

Fig6B. The raw data of western blotting. The boxed regions were shown in this article.

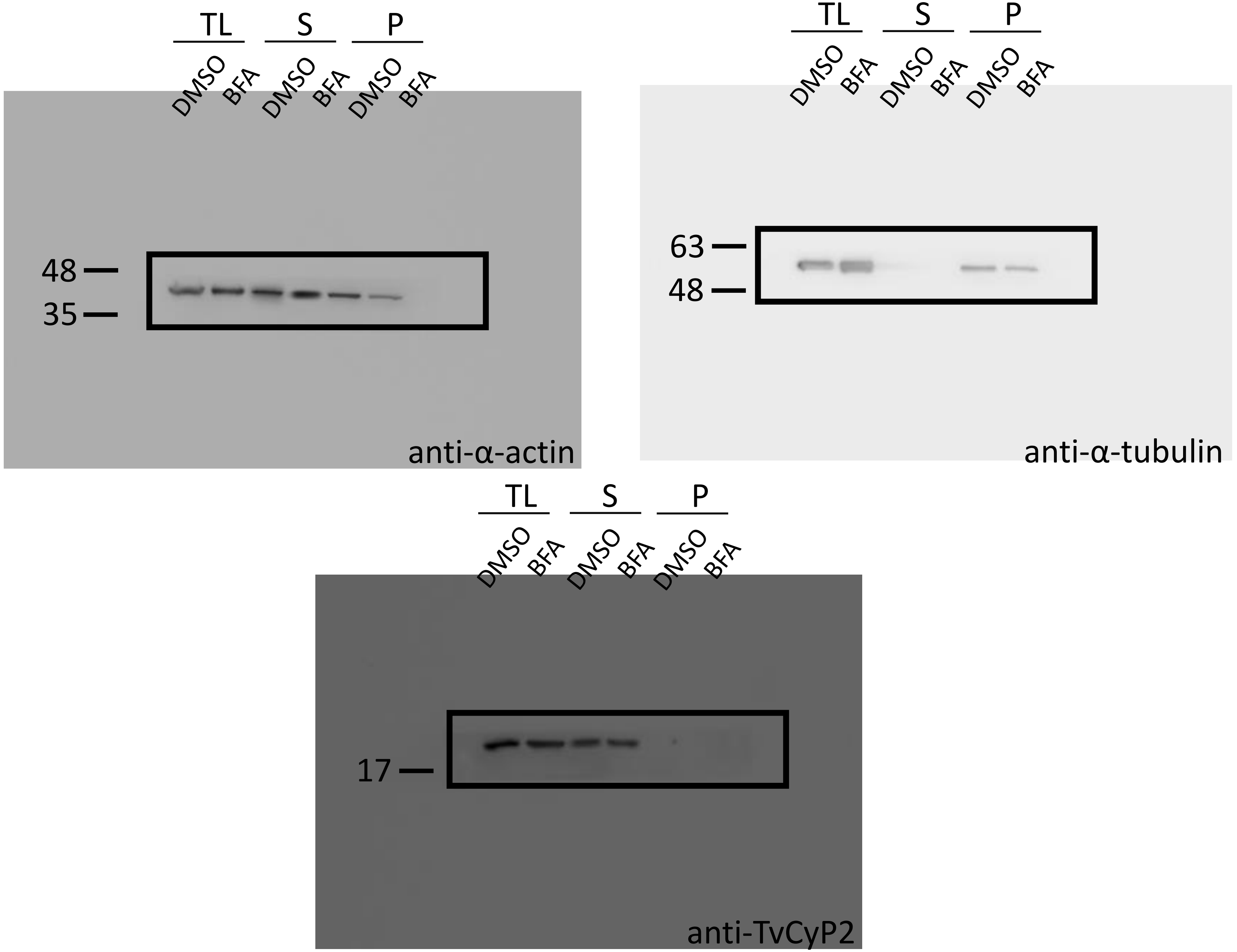

Fig6C. The raw data of western blotting. The boxed regions were shown in this article.

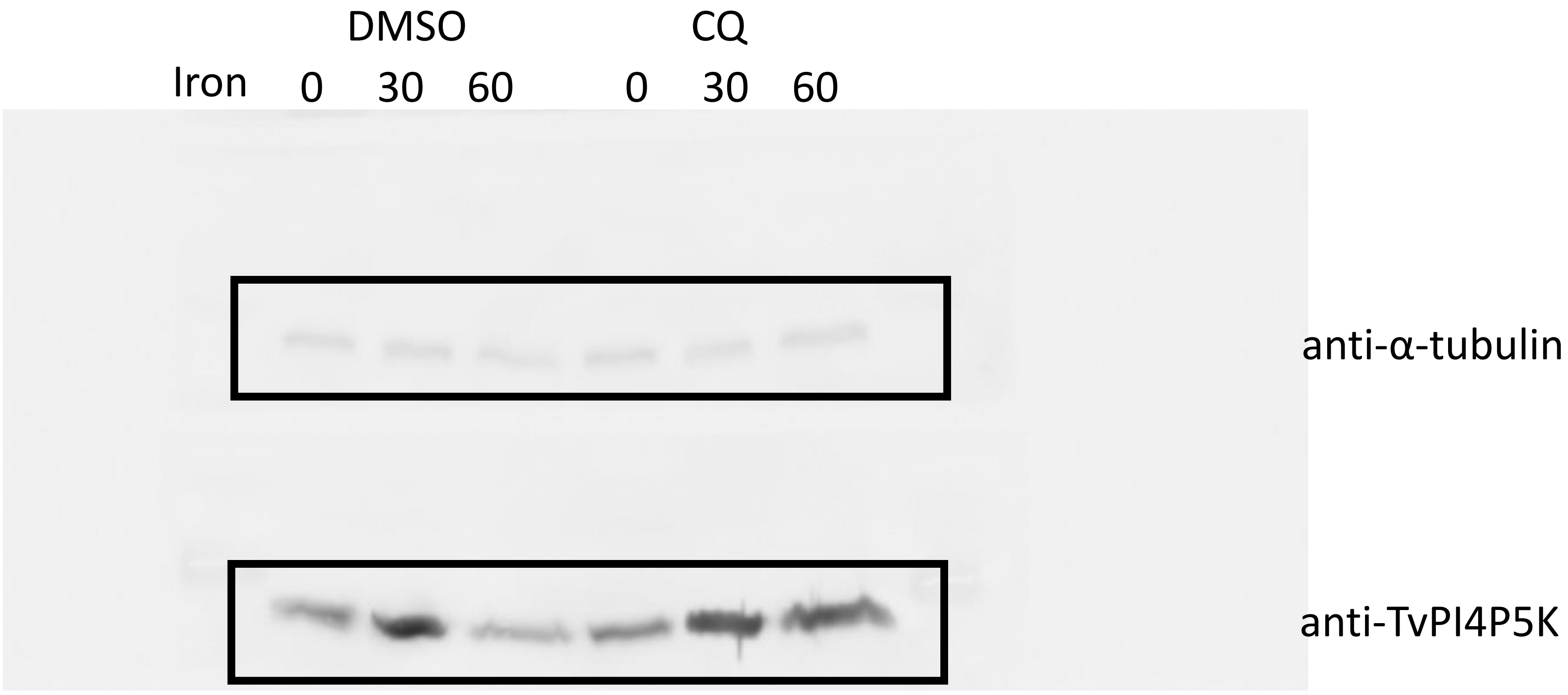

Fig S2A. The raw data of western blotting. The boxed regions were shown in this article.

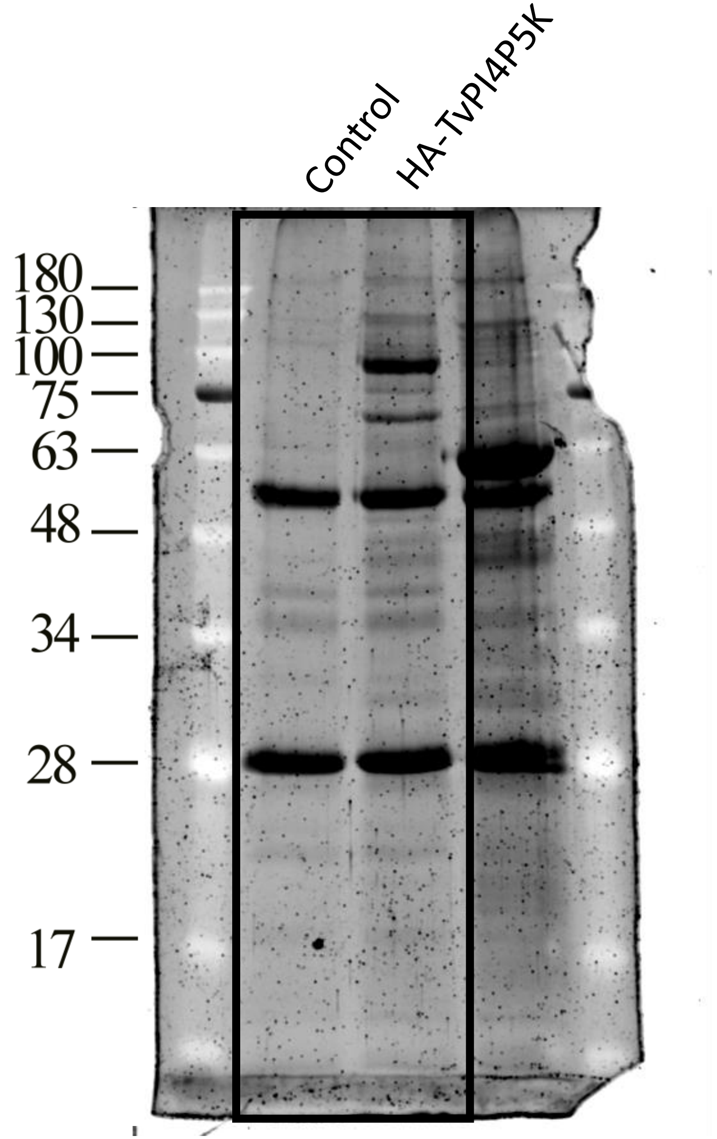

Fig S3A. The raw data of SyproRuby staining . The boxed regions were shown in this article.

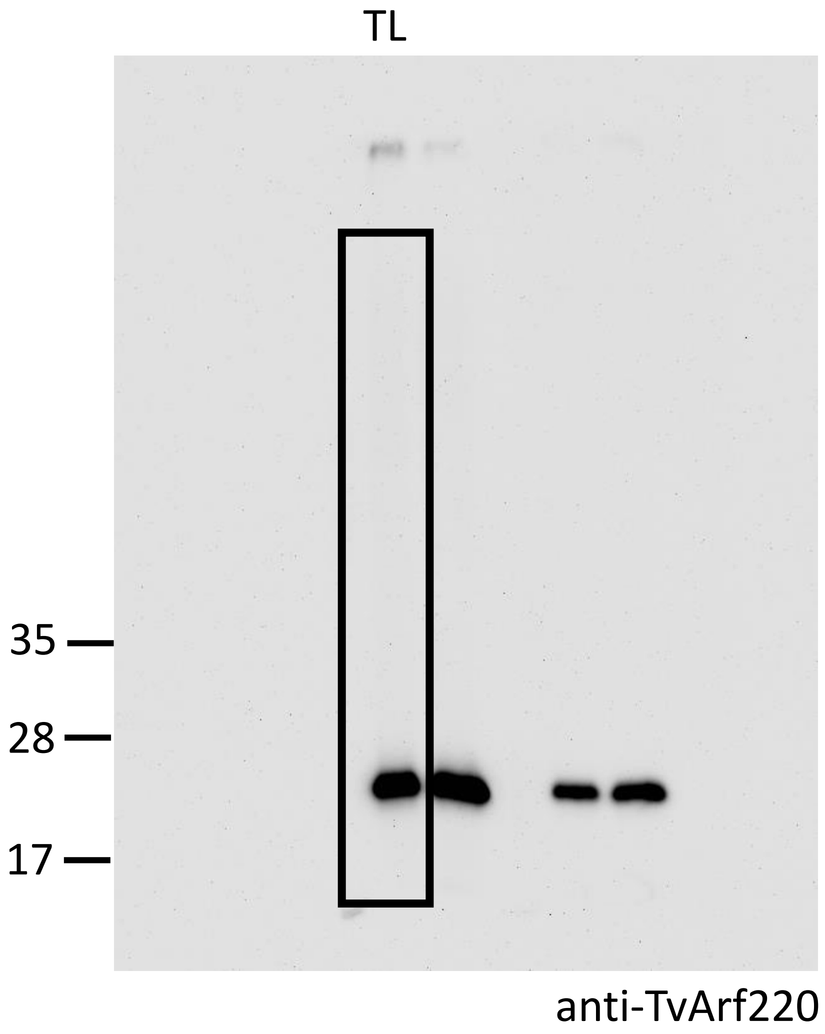

Fig S3D. The raw data of western blotting. The boxed regions were shown in this article.

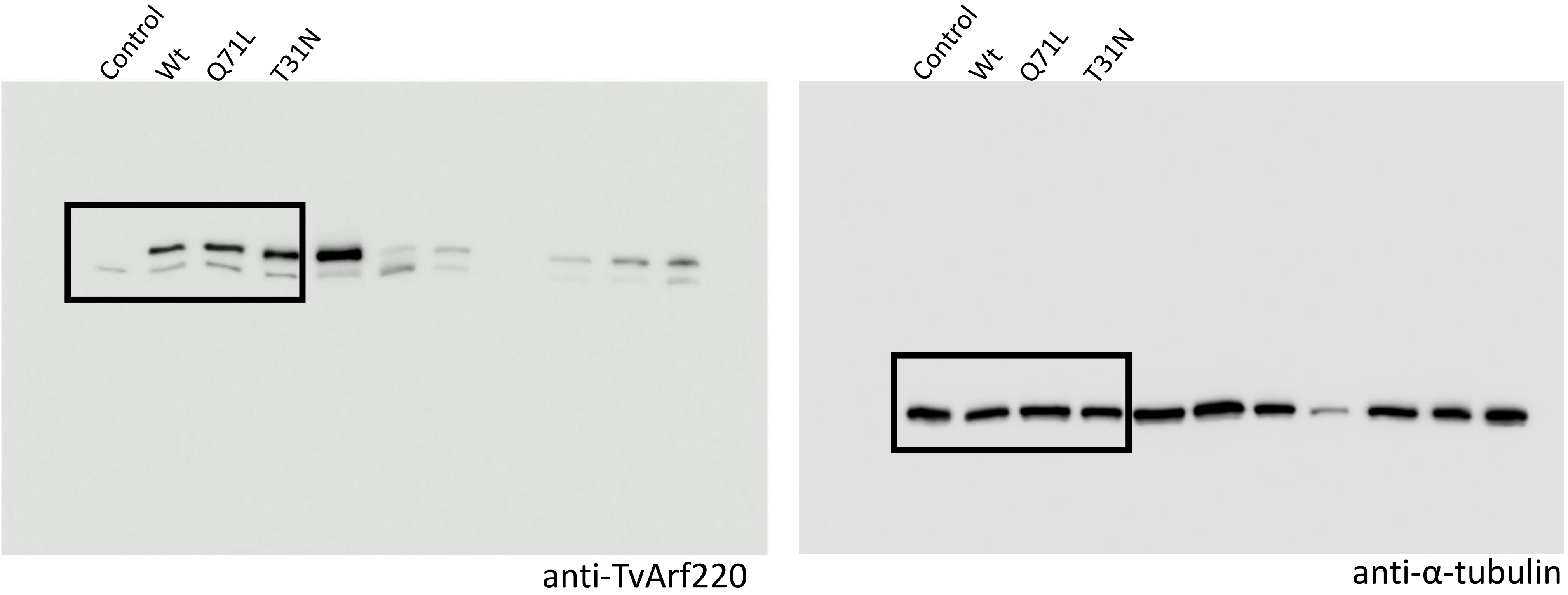

Fig S3E. The raw data of western blotting . The boxed regions were shown in this article.

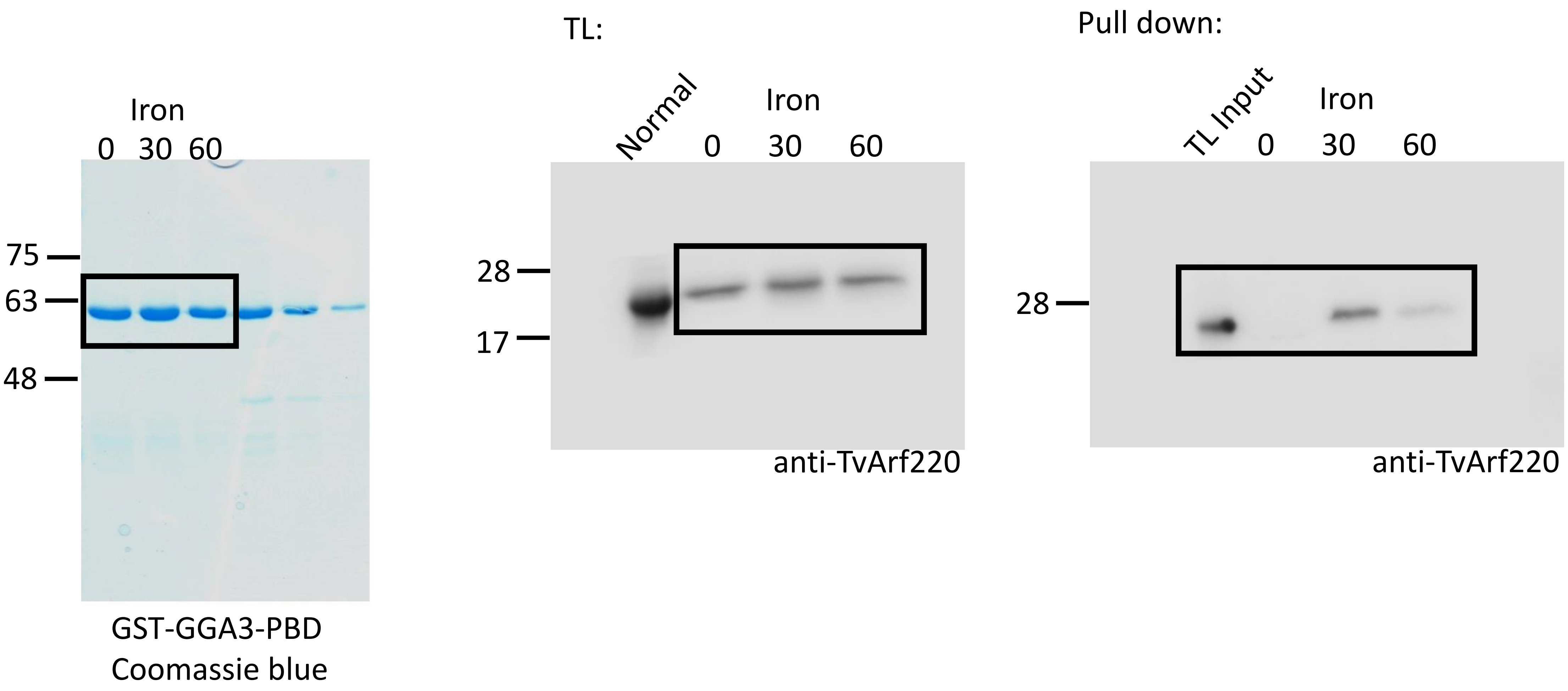

Fig S3F. The raw data of Coomassie blue staining and western blotting . The boxed regions were shown in this article.

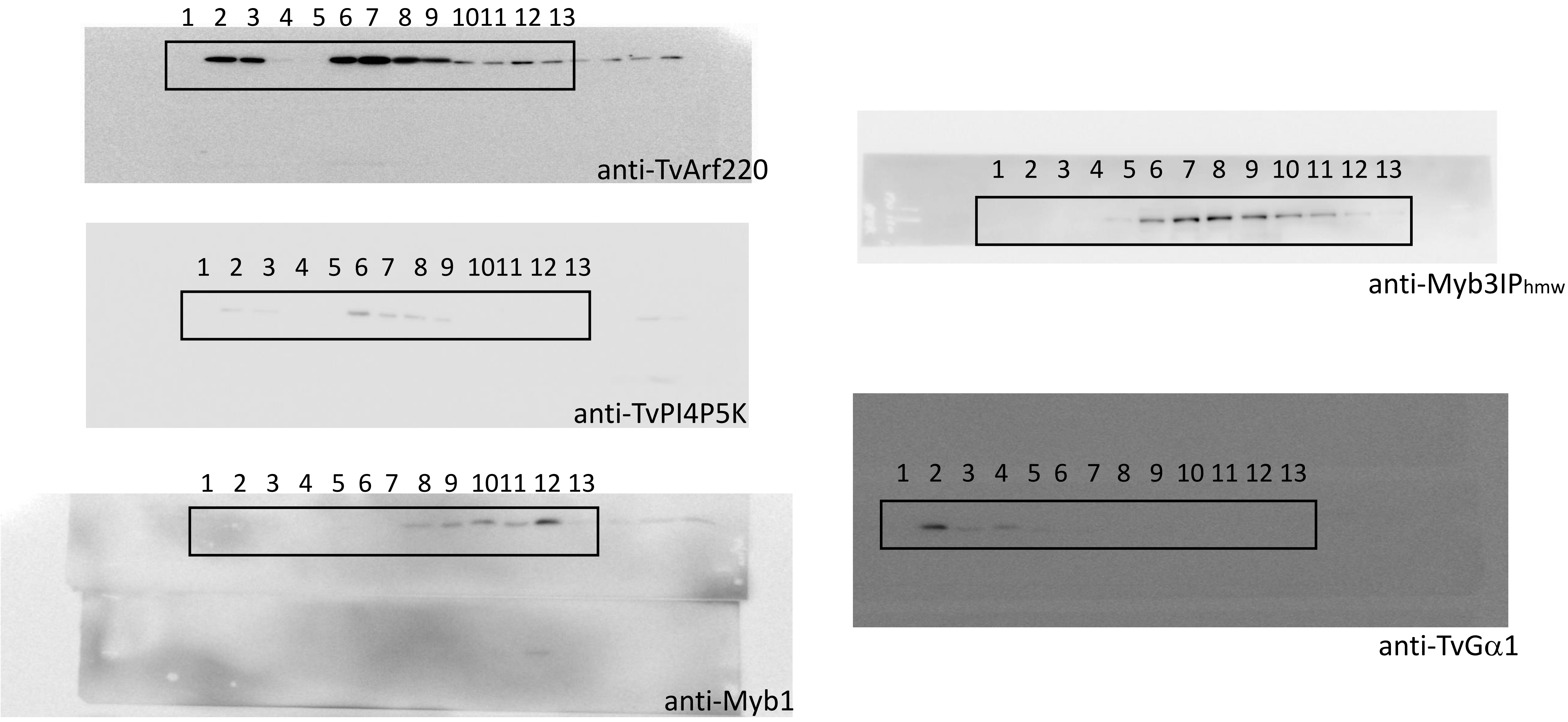

Fig S4C. The raw data of western blotting . The boxed regions were shown in this article.

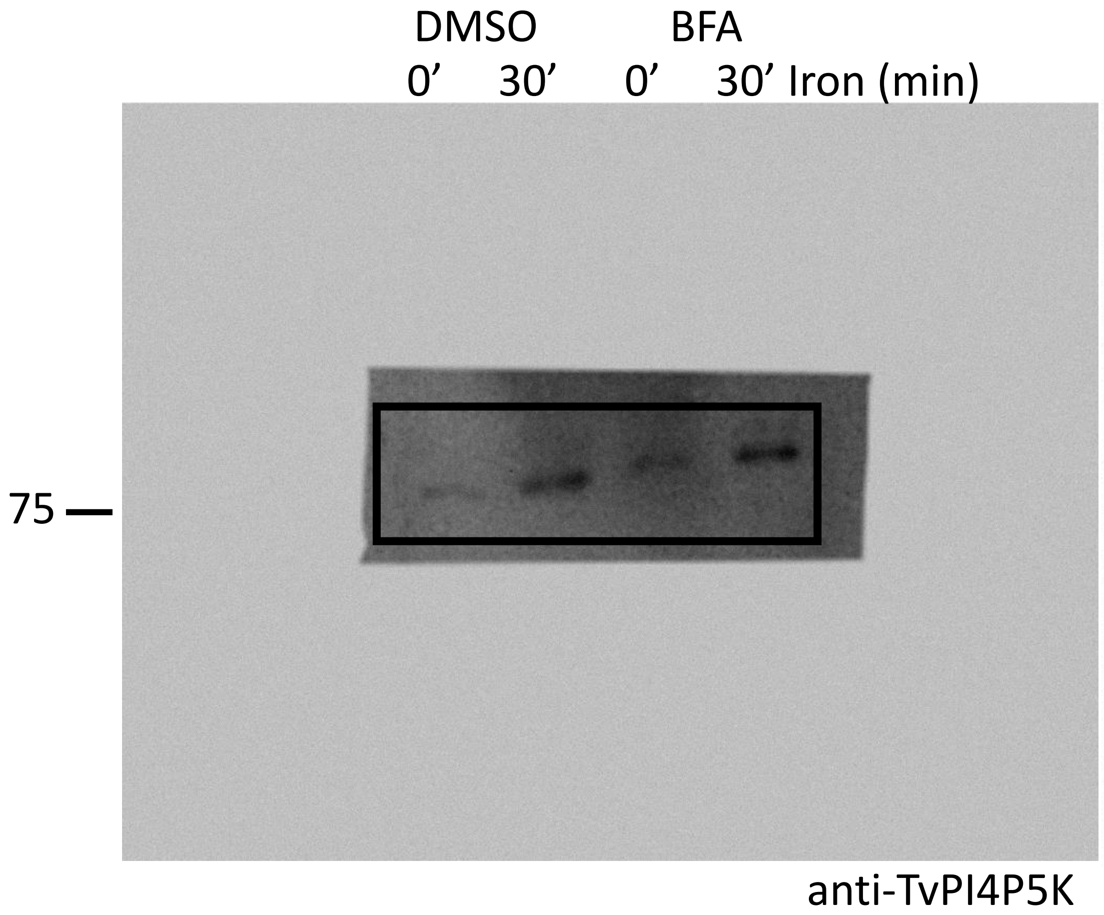

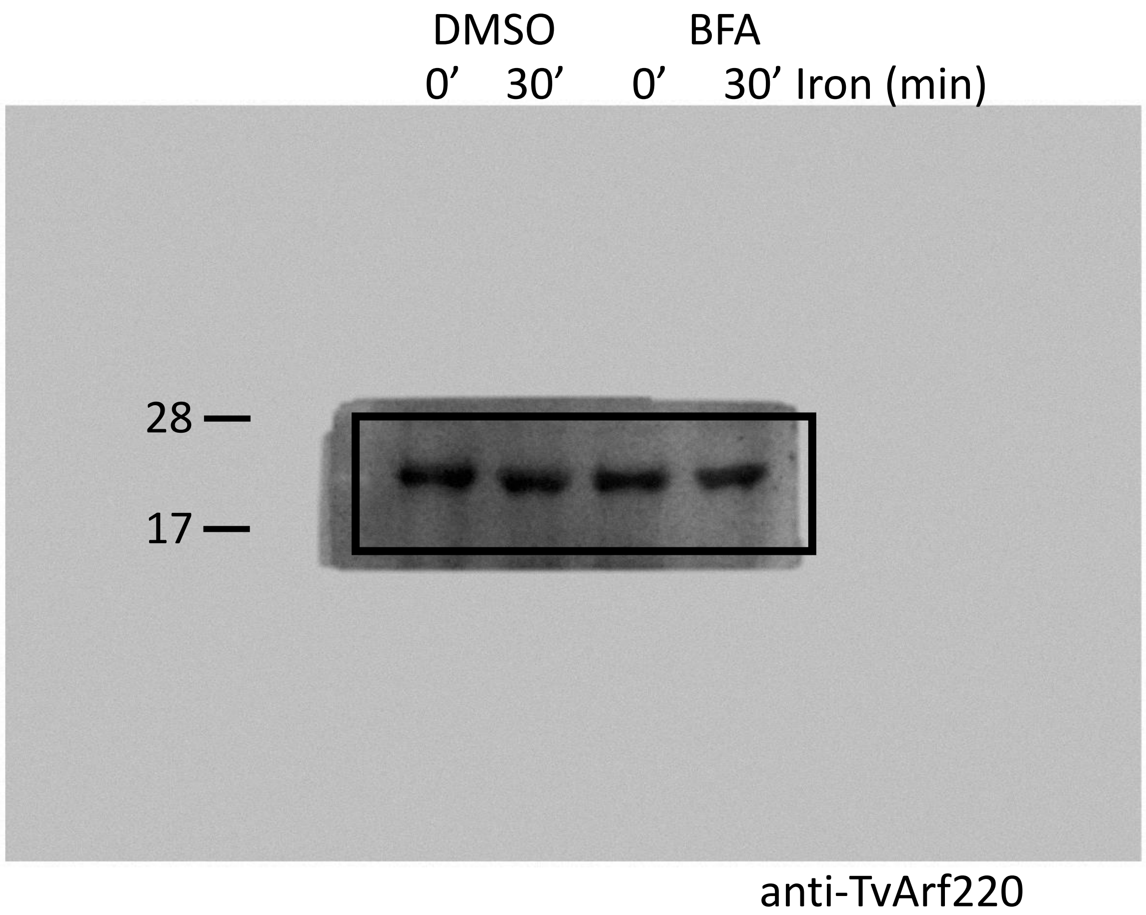

Fig S5D. The raw data of western blotting . The boxed regions were shown in this article.
