# Supplementary material for: Iron triggers TvPI4P5K proteostasis and Arf-mediated cell membrane trafficking to regulate PIP2 signaling crucial for multiple pathogenic activities of the parasitic protozoan Trichomonas vaginalis
Source: mBio. 2024 Dec 23;16(2):e01864-24. doi: 10.1128/mbio.01864-24 (PMC11796385; doi:10.1128/mbio.01864-24)
Supplement: Supplemental figures — Fig. S1 to S9. [file mbio.01864-24-s0004.docx]

**Supporting information**

**Iron triggers *Tv*PI4P5K proteostasis and Arf-mediated cell membrane trafficking to regulate PIP_2_ signaling crucial for multiple pathogenic activities of the parasitic protozoan *Trichomonas vaginalis***

Kuan-Yi Wu^1,#^, Yen-Ju Chen^1,#^, Shu-Fan Lin^1,#^, and Hong-Ming Hsu^1,*^

^1^ Department of Tropical Medicine and Parasitology, College of Medicine, National Taiwan University, Taipei, Taiwan 100233.

^#^ These authors contributed equally to this work.

*Address correspondence to:

Hong-Ming Hsu, Department of Tropical Medicine and Parasitology, College of Medicine, National Taiwan University, Taipei, Taiwan 100. Tel: 886-2-23123456 ext. 288260; Fax: 886-2-23915294; E-Mail: hsuhm@ntu.edu.tw

**Key words:** Iron, PIP_2_, lysosomal degradation, ADP ribosylation factor, plasma membrane trafficking, Actin cytoskeleton, Pathogenicity, *Trichomonas vaginalis.*

**Running title:** Iron-regulated PIP_2_ signaling modulates *T. vaginalis* pathogenicity.

**Fig. S1**


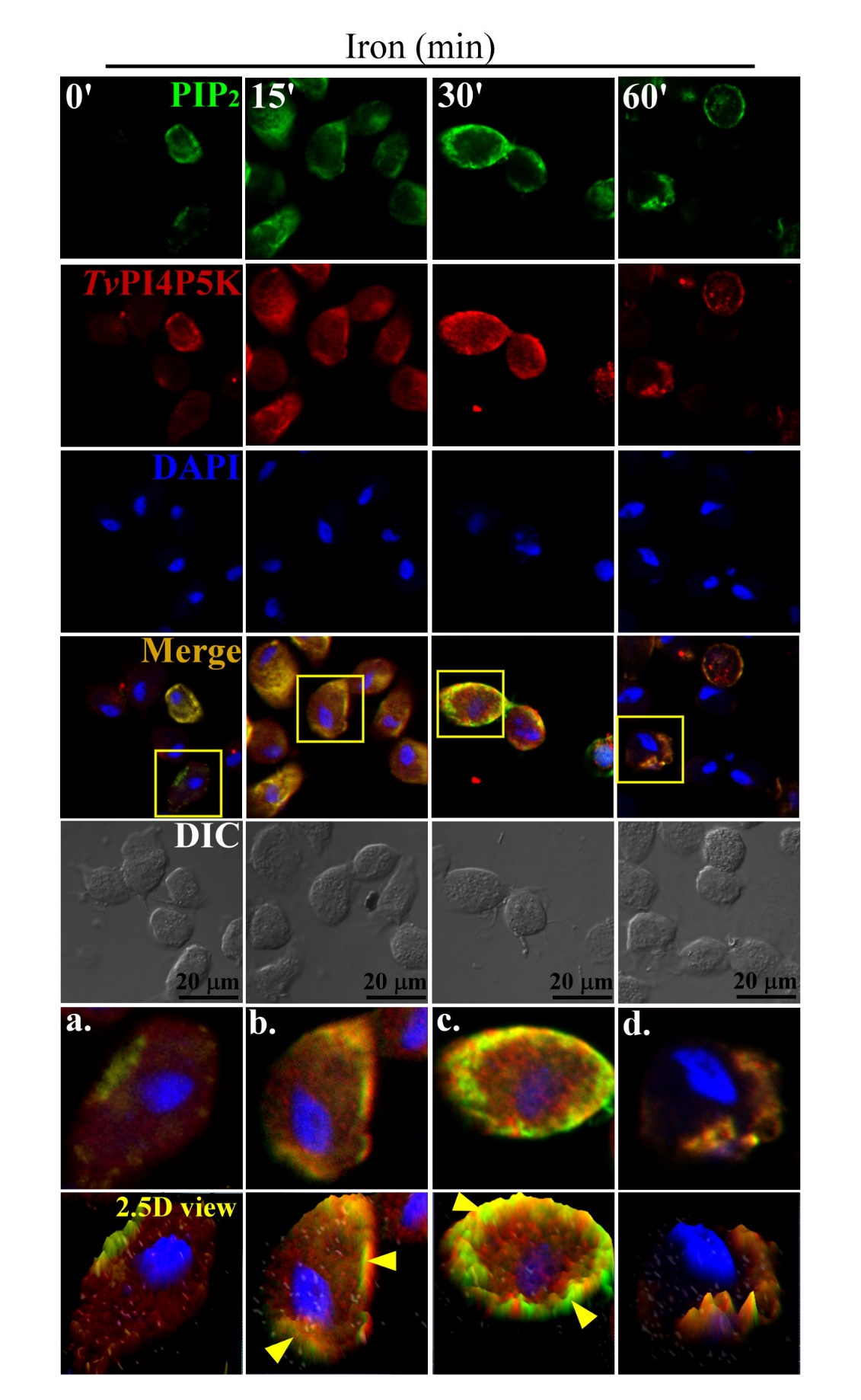


**Figure S1. Iron-inducible *Tv*PI4P5K and PIP_2_ signal colocalization in the *T. vaginalis* plasma membrane.** The uncropped confocal images of Fig. 1A included multiple trophozoites. The boxed regions were magnified (a.-d.) and converted to the 2.5D view images by Zen software to highlight the signals colocalized around the plasma membrane (yellow arrowheads).

**Fig. S2**


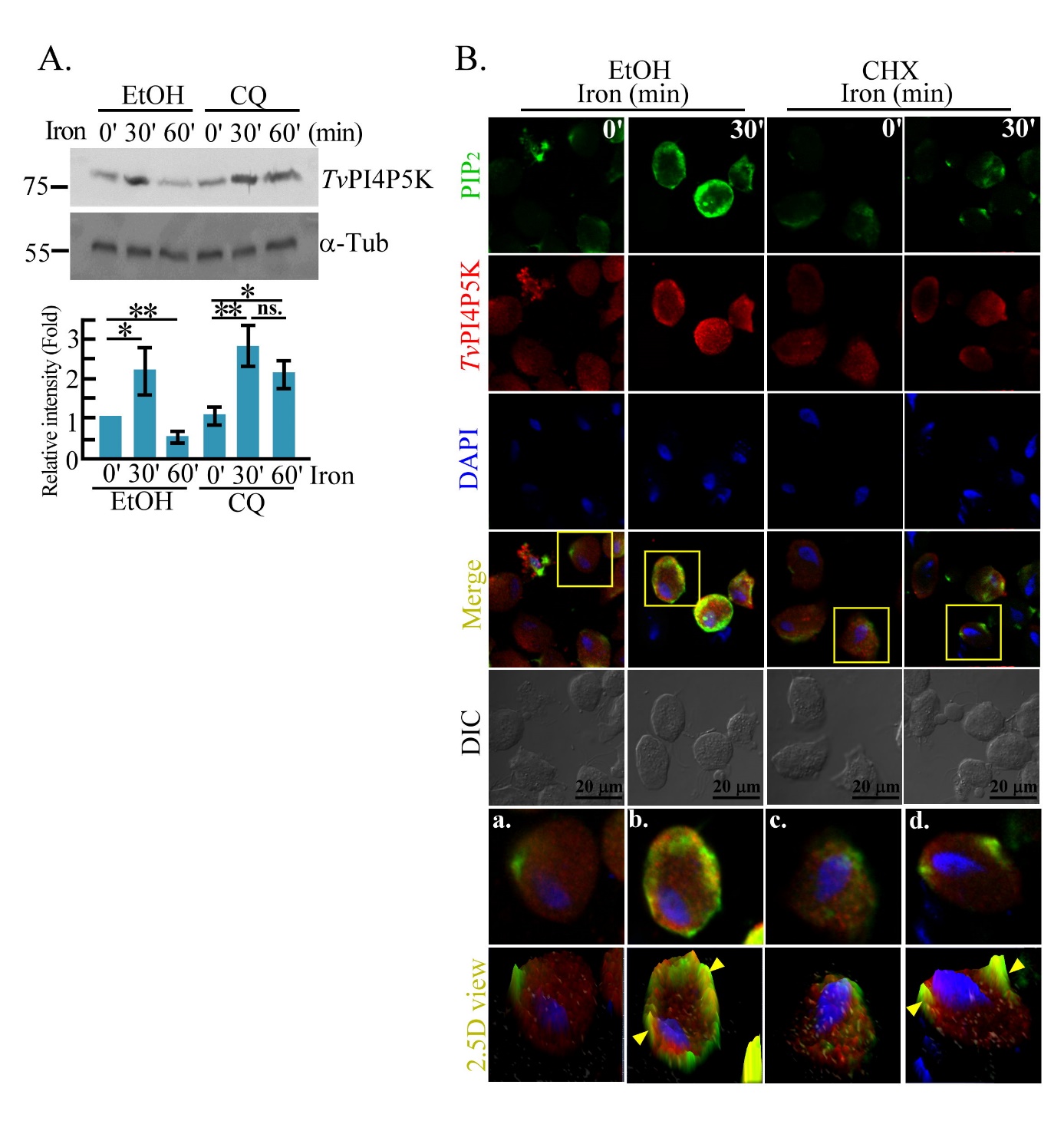


**Figure S2. Cycloheximide inhibits iron-inducible *Tv*PI4P5K expression in *T. vaginalis*.**

1. The iron-depleted parasites were pretreated with ethanol (EtOH) or chloroquine (CQ) and then replete with iron. The total lysates collected from the trophozoites at different time points were subjected to western blotting using anti-*Tv*PI4P5K or anti-α-tubulin antibodies. The relative signal intensities from three biological repeats were quantified as shown in the bar graphs (n=3, mean ± SD) and statistically analyzed by Student's t-tests, with *p<* 0.05(*) and *p<* 0.01(**), and ns, no significance. The uncropped confocal images of Fig. 2D were shown in (B.). The boxed regions were magnified (a.-d.) and converted to the 2.5D view images by Zen software to show the colocalized signals around the plasma membrane (yellow arrowheads).

**Fig. S3**


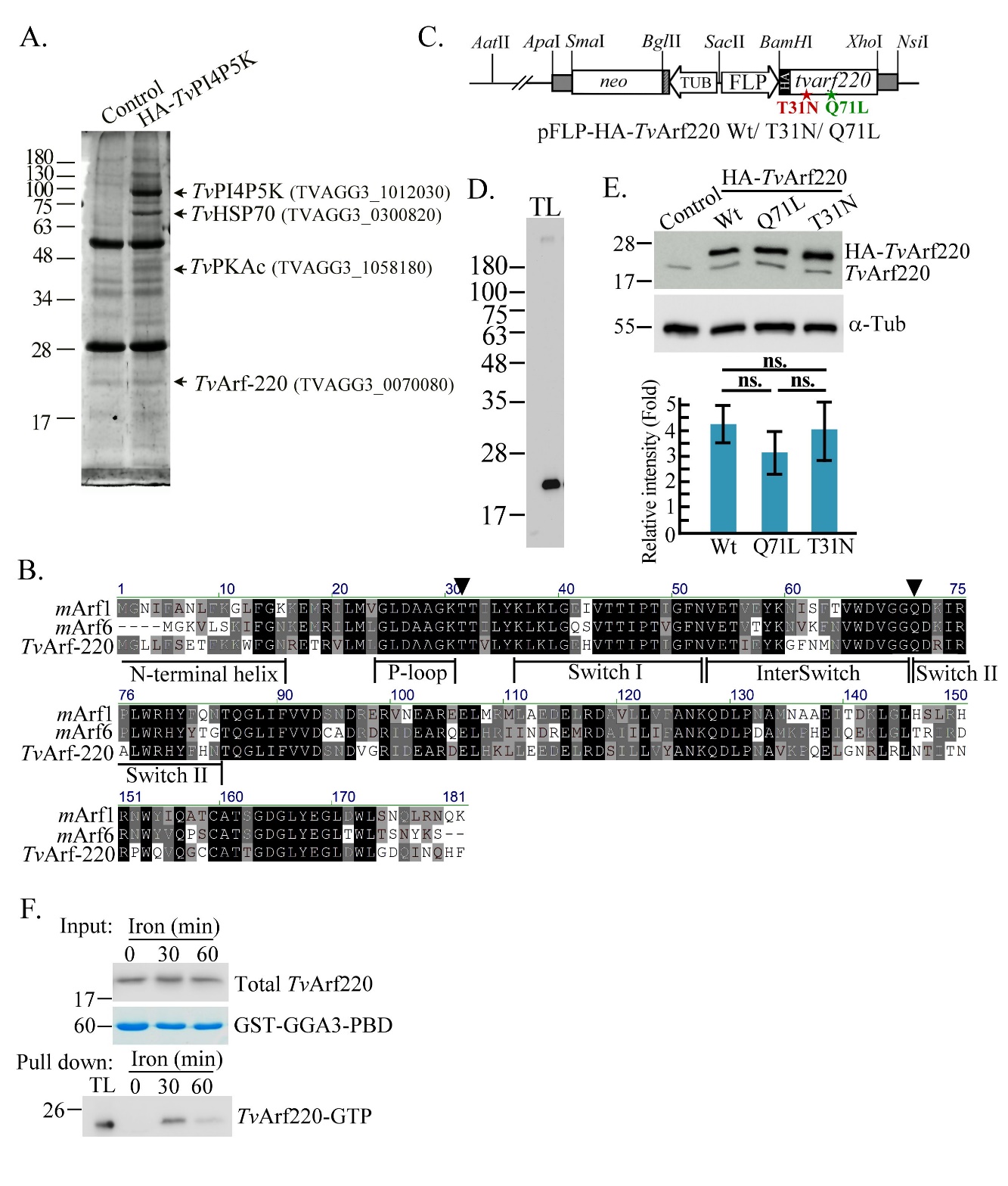


**Figure S3. Identification of HA-*Tv*PI4P5K interacting components.** (A.) The anti-HA antibody immunoprecipitants from the non-transgenic control and HA-*Tv*PI4P5K transfectant were separated in a 12% gel by SDS PAGE for SYPRO Ruby staining and gel-based mass spectrometry. The arrows indicate the bands corresponding to the protein of interest identified by LC-MS/MS with accession numbers in parentheses. (B.) The protein sequences of *T. vaginalis* *Tv*Arf220 (TVAGG3_0070080), mouse *m*Arf1 (P84078) and mouse *m*Arf6 (P62331) were aligned, and the consensus sequences are highlighted. The amino acid residues of Thr^27^ and Gln^67^ in *m*Arf6 conserved with Thr^31^ and Gln^71^ in *Tv*Arf-220, respectively, are indicated by the downward arrowheads. The conserved regions of the N-terminal, P-loop, Switch I, Switch II, and InterSwitch domains are marked at the bottom of the sequences. (C.) The vector with a target gene driven by the FLP promoter and a selective *neo* gene driven by the TUB promoter was constructed to overexpress the wildtype (Wt), T31N, and Q71L of HA-*Tv*Arf220. (D.) Total parasite lysates were subjected to western blotting using an anti-*Tv*Afr220 antibody. (E.) The total lysates from non-transfectant and various HA-*Tv*Arf220 transfectants were subjected to western blotting using anti-*Tv*Arf220 and anti-α-tubulin antibodies. The relative intensities of HA-*Tv*Arf220 versus endogenous *Tv*Arf220 signals were quantified from three biological repeats as shown in the bar graph (n=3, mean ± SD) and statistically analyzed by Student's t-tests (ns, no significance). (F.) The total parasite lysates before and after iron repletion for 30 or 60 min were incubated with the resin conjugated with GST-GGA3-PBD. The total lysates (input) and pull-down samples (pull down) were subjected to western blotting detection using an anti-*Tv*Arf220 antibody. GST-GGA3-PBD protein inputs were examined by Coomassie blue staining.

**Fig. S4**


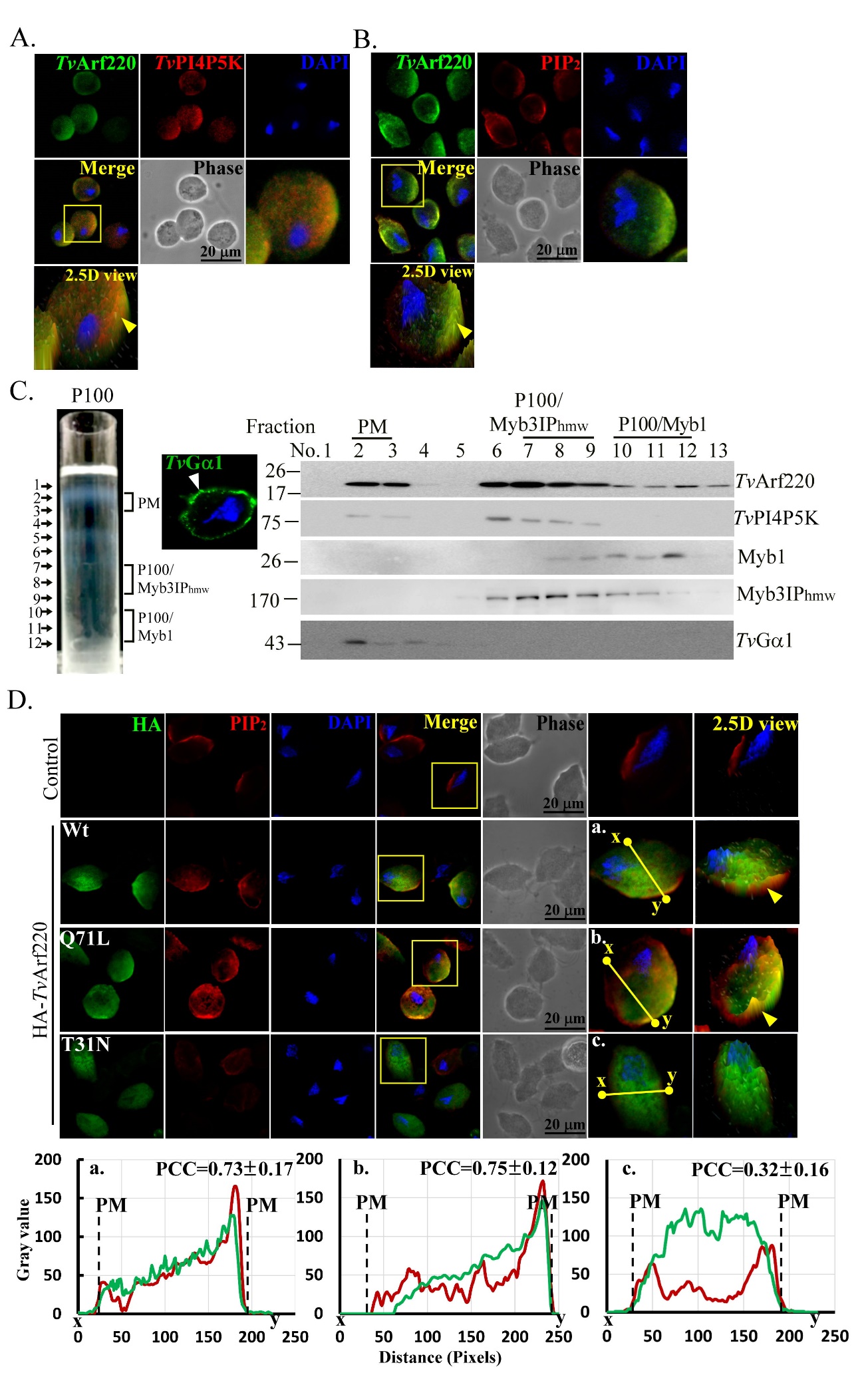


**Figure S4. *Tv*Arf220 regulates *Tv*PI4P5K plasma membrane trafficking in *T. vaginalis*.** The uncropped confocal images of Fig. 3D and Fig.3E were shown in (A.) and (B.), respectively. The boxed regions were magnified and converted to 2.5D view images by Zen software. (C.) P100 lysate fractionated by OptiPrep ultracentrifugation (left panel) were collected to 200-μl aliquots from the top gradient for western blotting detection (right panel). The inset IFA was stained with anti-*Tv*Gα1 antibody, and arrowhead indicates its plasma membrane localization (middle panel). The membrane compartments markers of P100/Myb1, P100/Myb3IP_hmw_, and the plasma membrane (PM) were detected using Myb1, Myb3IP_hmw_, and *Tv*Gα1, respectively, as reported in reference (27). (D.) The various HA-*Tv*Arf220 transfectants at normal medium were fixed for IFA double-staining with anti-*Tv*Arf220 and anti-PIP_2_ antibodies. The boxed regions were magnified (a.-c.) and converted to 2.5D view images by Zen software to highlight the colocalized signals peripheral to the plasma membrane (yellow arrowheads). The signal intensities between the x and y sites on the yellow lines of the representative images were assayed with ImageJ as shown in the corresponding plots (a.-c.). The PCC values measured from 30 trophozoites of three independent microscopic fields. The averaged PCC values from three biological repeats were shown in the plots (a.-c.) (n=3, mean ± SD). PM indicates the plasma membrane boundary.

**Fig. S5**


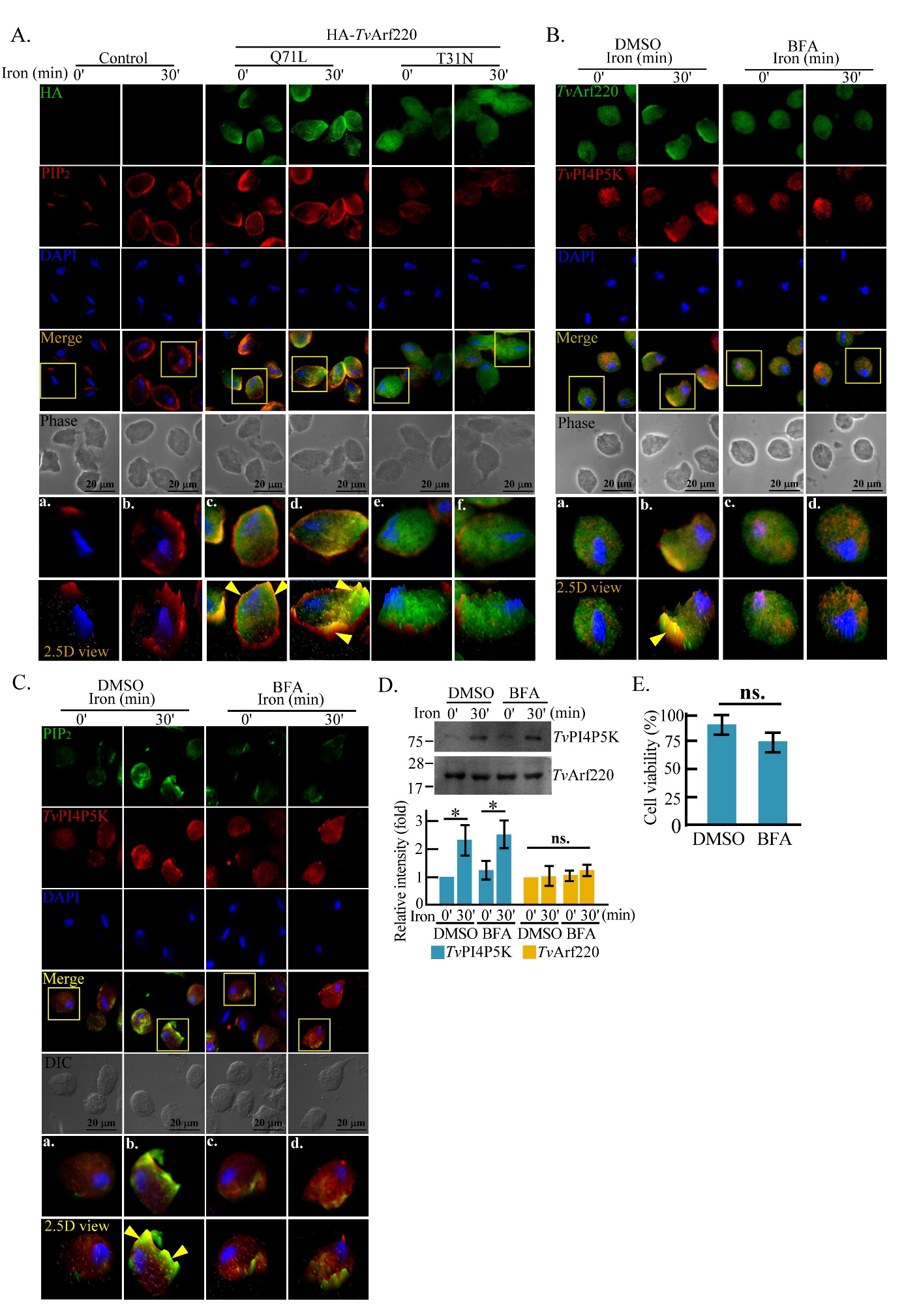


**Figure S5. *Tv*Arf220 functions in iron-inducible *Tv*PI4P5K plasma membrane trafficking and PIP_2_ production.** The uncropped confocal images of Figs. 4A, 4B, and 4C were shown in (A.), (B.), and (C.), respectively. The boxed regions were magnified and converted into the 2.5D view images by Zen software to highlight the signals colocalized around the plasma membrane (yellow arrowheads). (D.) The total lysates from the parasites pretreated without or with BFA before and after iron activation, were subjected to western blotting with anti-*Tv*PI4P5K or anti-*Tv*Arf220 antibodies. The relative intensity was quantified as shown in the bar graph. (E.) The viability of parasites pretreated with DMSO or BFA was assessed by the trypan blue exclusion assay. The assays were performed in triplicate (n=3, mean ± SD) and statistically analyzed by Student’s t-tests with *p<* 0.05(*), *p<* 0.01(**), and ns, no significant difference.

**Fig. S6**


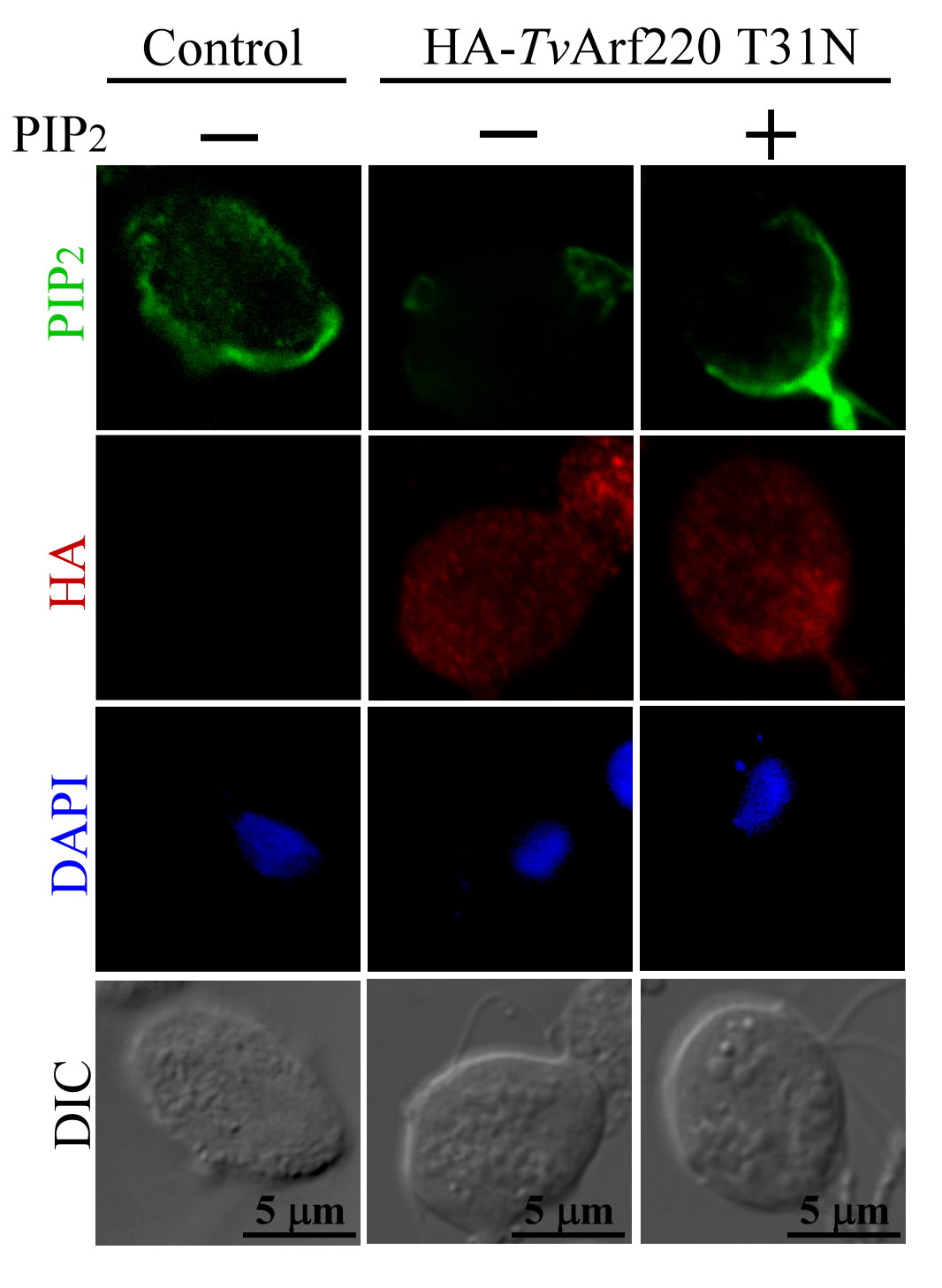


**Figure S6. PIP_2_ delivery into *T. vaginalis* trophozoites*.*** PIP_2_ was delivered into the trophozoites of the *T. vaginalis* non-transgenic control or HA-*Tv*Arf220 T31N transfectants, as described in the materials and methods. The parasites were fixed and double-stained with anti-PIP_2_ and anti-HA antibodies for IFA. The nuclei were stained with DAPI and the morphology was recorded by confocal microscopy in DIC mode. The scale bar represents 5 µm.

**Fig. S7**


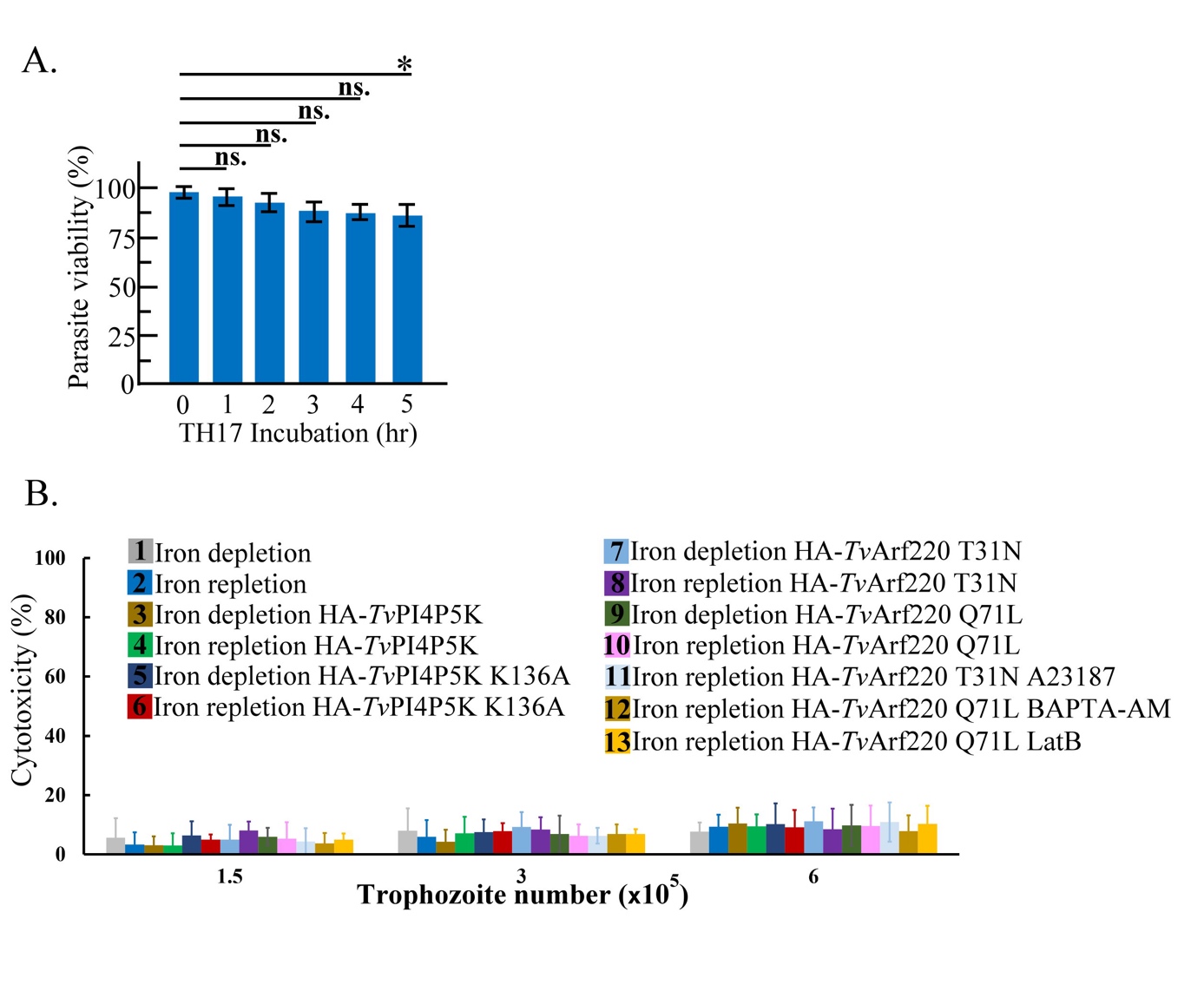


**Figure S7. Parasite viability in cytotoxicity assay.** The parasites were cultured in 96-well microplates at 37℃ under atmosphere with 5% CO_2_. (A.) The TH17 cultures were collected at indicated time points and cell viability was assessed using a trypan blue exclusion assay. The percentage of viable cells was measured from 300 trophozoites within five independent microscopic fields. Viability was averaged from three biological replicates as shown in the bar graph (n=3, mean ± SD) and statistically analyzed by Student’s t-tests with *p<* 0.05(*), *p<* 0.01(**), and ns, no significant difference. (B.) In the absence of *h*VECs, the parasites pretreated as described in Fig. 8A, were assessed for LDH cytotoxicity. The trophozoites treated with Lysis Solution was detected as 100% cytotoxicity, and the relative cytotoxicity averaged from three biological repeats was shown in the bar graph (n=3, mean ± SD).

**Fig. S8**


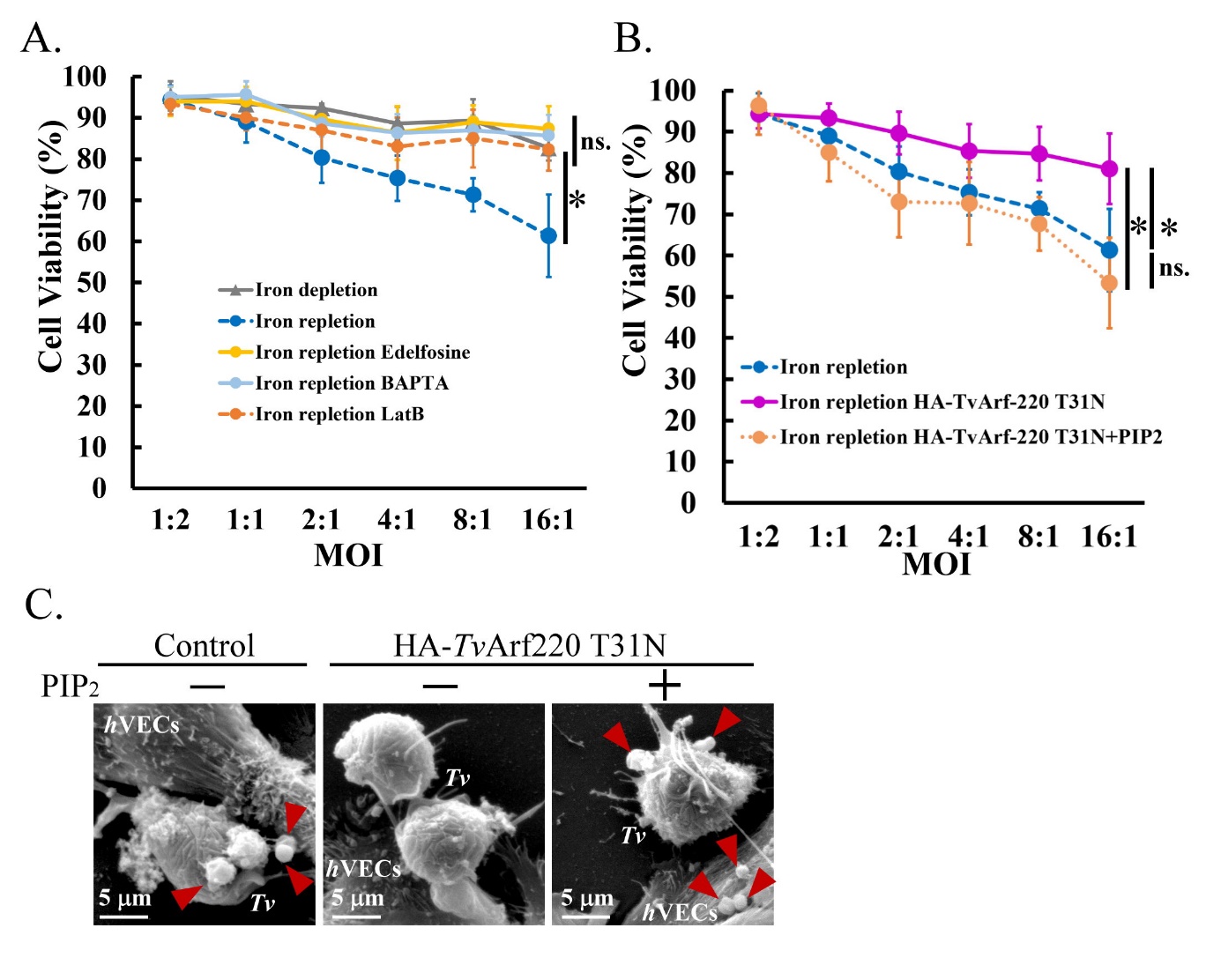


**Figure S8. The roles of the PIP_2_-calcium signaling cascade and actin cytoskeleton in the contact-independent cytotoxicity of *T. vaginalis*.** In a trans-well co-culture, the viabilities of *h*VECs inoculated with Edelfosine, BAPTA-AM, and LatB treated parasites (A.) or the HA-*Tv*Arf220 T31N transfectant with or without PIP_2_ replenishment (B.) at different MOIs in the presence or absence of iron were evaluated by the MTT assay. The assays were performed in triplicate (n=3, mean ± SD) and statistically analyzed by Student’s t-tests with *p<* 0.05(*), *p<* 0.01(**), and ns, no significant difference. (C.) The non-transgenic control parasite and the HA-*Tv*Arf220 T31N transfectant replenished with or without PIP_2_ were sampled for SEM to observe the extracellular vesicle-like particles (red arrowheads). *Tv*, *T. vaginalis*. *h*VECs, human vaginal epithelium cells. The scale bar represents 5 μm.

**Fig. S9**


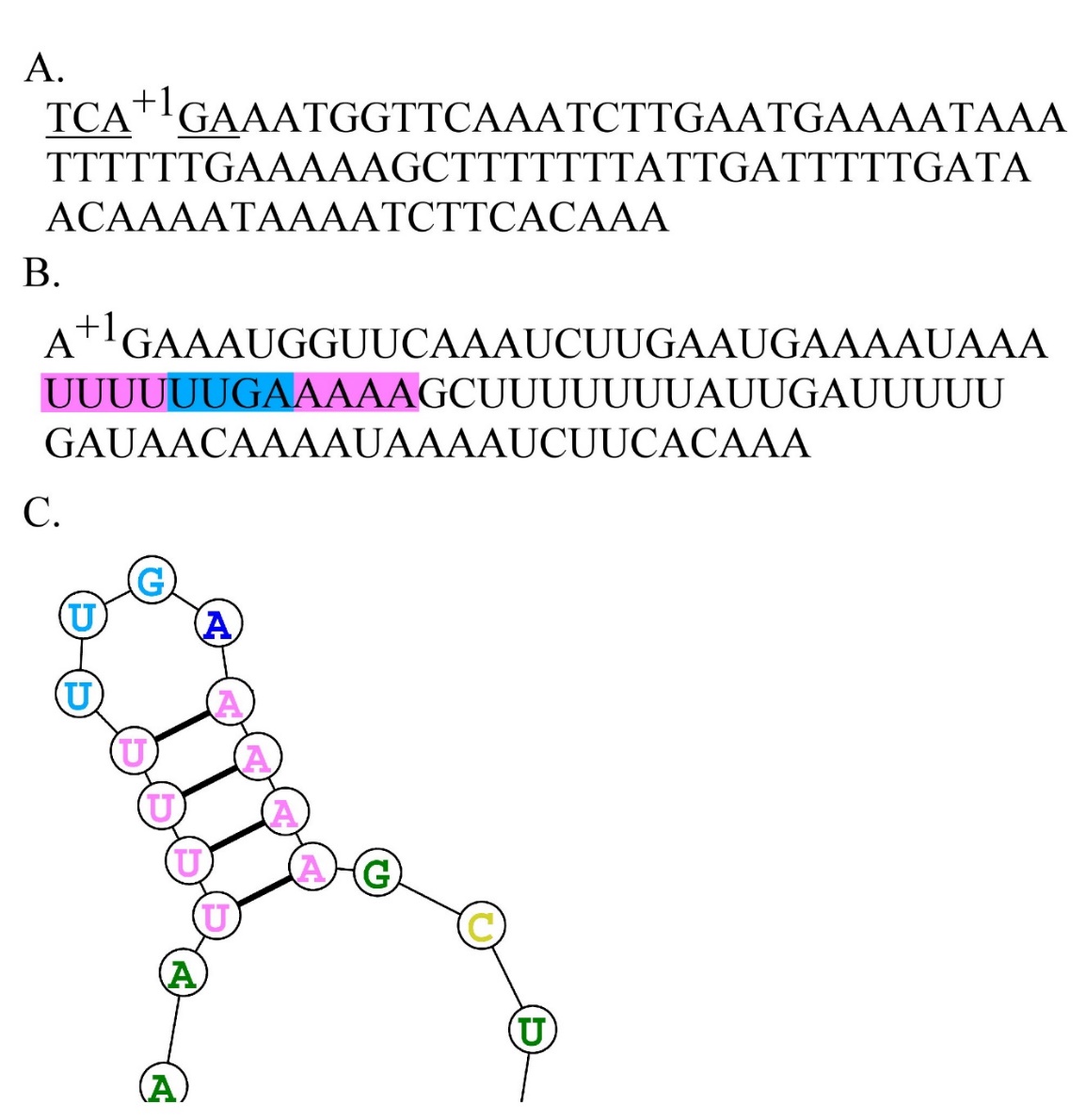


**Figure S9. Prediction of IRE in the 5′ UTR of the *tvpi4p5k* gene.** (A.) The DNA sequence shows the 5′UTR preceding the *tvpi4p5k* gene. The potential initiator is underlined, and the containing transcription start site is defined as (+1). (B.) The RNA sequence transcribed from (A.) was simulated by the RNA secondary structure prediction web server (<https://rna.urmc.rochester.edu/RNAstructureWeb/Servers/Predict1/Predict1.html>). The potential sequences forming stem and loop structures are highlighted in pink or blue, respectively, and the simulated structure was shown in (C.)
