# Supplementary material for: Iron triggers TvPI4P5K proteostasis and Arf-mediated cell membrane trafficking to regulate PIP2 signaling crucial for multiple pathogenic activities of the parasitic protozoan Trichomonas vaginalis
Source: mBio. 2024 Dec 23;16(2):e01864-24. doi: 10.1128/mbio.01864-24 (PMC11796385; doi:10.1128/mbio.01864-24)
Supplement: Supplemental captions — Captions for Data S1, S2, and S3. [file mbio.01864-24-s0005.docx]

**Supplemental legends for Data S1, S2, and S3**

**Data S1. FLP promoter sequence (-786 to +11).** This file contains the DNA sequence of the FLP promoter from positions -786 to +11, including the transcription initiator (Inr).

**Data S2. Source raw data-Statistical analysis.** This file includes all raw data for statistical analyses and quantifications presented in this study.

**Data S3. Source raw data-gels and blots.** Uncropped gel or western blotting images are provided in this file.
